# Cryo‐EM Structure Guided Engineering of Botulinum Neurotoxin A With Advanced Receptor Binding Affinity and Therapeutical Benefits

**DOI:** 10.1002/advs.202516713

**Published:** 2026-04-07

**Authors:** Wenrui Wang, Zhaxi Zerang, Linjin You, Ziye Liu, Rong Nie, Fuwei Qi, Fenfen Gao, Chengmu Zhao, Wantong Ma, Jinghan He, Xiaoru Wang, Shanquan Wu, Bo Liu, Xinyao Liu, Dongsheng Lei, Dejuan Zhi, Dongsheng Wang

**Affiliations:** ^1^ School of Pharmacy Joint Drug Development and Innovation Centre For Neurological Disorders of Lanzhou University‐China National Biotec Group‐Lanzhou Biotechnology Development Co. Lanzhou University Lanzhou Gansu P. R. China; ^2^ MOE Frontiers Science Center for Rare Isotopes Lanzhou University Lanzhou Gansu P. R. China; ^3^ School of Physical Science and Technology Electron Microscopy Center of Lanzhou University Lanzhou University Lanzhou Gansu P. R. China

**Keywords:** cryo‐EM, engineered botulinum toxin A, higher affinity, higher potency, less toxic

## Abstract

Botulinum neurotoxin type A (BoNT/A) has been extensively used in treating a wide range of neurological disorders and aesthetics. However, insufficient binding affinity between BoNT/A and its receptor SV2C could lead to mild to severe side effects. An open active conformation of BoNT/A cryo‐EM structure at a resolution of ∼2.85 Å was resolved. Guided by the BoNT/A cryo‐EM structure, saturation mutagenesis libraries were constructed and screened by BACTH system to identify mutants that can boost toxin‐SV2C affinity. The engineered toxin binding domain achieved up to approximately six‐fold improved affinity in SPR analysis, and the engineered toxins exhibited significantly improved binding capacity, and enhanced SNAP25 cleavage efficacy in cultured neurons. Preclinical animal studies, including MLB, DAS, sweat test, and PET/CT assays, demonstrated that the engineered BoNT/A VLTS has higher potency, lower diffusion, and significantly better safety profiles than the BoNT/A wt, which can reduce side effects and benefit future therapeutic applications.

## Graphical abstract

The butterfly unfolded wing in an open form structure of botulinum neurotoxin type A (BoNT/A) at physiological‐state was confirmed at 2.85 Å resolution by cryo‐electron microscopy (cryo‐EM). Structure‐guided protein engineering significantly enhanced the receptor‐binding affinity, therapeutic efficacy, and safety of the engineered toxin variants.

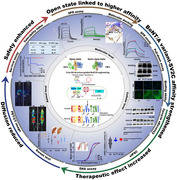

## 1 Introduction

Botulinum neurotoxins (BoNTs) are bacterial proteins produced by the anaerobic bacterium *Clostridium botulinum* and are considered the most potent toxins known to exist [1]. Eight distinct BoNT serotypes (A‐G, X) have been identified, with further subtype variations within each serotype, such as BoNT/A1‐/A8, BoNT/B1‐/B8, BoNT/E1‐/E12, BoNT/F1‐/F9, and BoNT/X [2, 3]. In 1989, the US Food and Drug Administration approved BoNT/A for the treatment of neurological disorders, including blepharospasm, strabismus, and cervical dystonia. Since then, BoNT/A has been expanded to therapeutic applications in musculoskeletal disorders, neurological injuries, chronic pain conditions, and aesthetic medicine [4, 5, 6, 7]. Treatment with BoNT/A is widely viewed to be safe and effective, while adverse reactions might occur in some cases, which could be caused by overdoses or systematic diffusion [8, 9, 10]. Based on French data from 1994–2020 for treating neurological disorders, the estimated adverse reaction incidence was 25–413 per 1 00 000 injection in France. Of which, botulism‐like severe systemic adverse reactions occurred at a proportion of 74%, exceeding the 26% rate of local adverse reactions [11]. Although short‐term BoNT/A treatment of less than 15 cycles, low‐dose cases reflected a low risk of neutralizing antibodies (NAbs) with an incidence of 0.5%, while 18.4% cervical dystonia patients that exposed to prolonged toxin treatment unavoidably developed NAbs, leading to decreased or lost therapeutic efficacy [12, 13]. In general, most local adverse reactions to BoNT/A are reported to be transient and manageable, and systemic complications are rare, but they can be potentially life‐threatening and thus require heightened vigilance [14]. These limitations and side effects have impact on the overall effectiveness of toxin therapy, and it is particularly pronounced when high‐dose toxin application is required [8], so reducing the treatment dose should be the preferred strategy to increase the benefit‐to‐risk ratio of BoNT/A.

Improving the binding affinity of BoNT/A with its receptor SV2 through genetic engineering is a practical approach to reduce the injection dose to address the toxin diffusion issue and improve therapeutic efficacy. For a less widely used toxin serotype BoNT/B, it has been reported that the screening of the heavy chain receptor binding domain (Hc/B) mutant library yields engineered BoNT/B with boosted binding affinity to its receptor h‐SytII by 11‐fold, leading to an increase in inhibition of neurotransmission and preclinical performances [15, 16]. Previous work has also shown that BoNT/A4 exhibits 1000‐fold lower potency than BoNT/A1 but without significant differences in SNAP25 cleavage capabilities of light chain catalytic activities. Mutating the different amino acids in the receptor‐binding domain of BoNT/A4 to the same amino acids with BoNT/A1 restores BoNT/A4 cleavage activity to the level comparable to BoNT/A1 [17]. Additionally, evidence suggests that BoNT/A2 displays more significant neuronal toxicity than BoNT/A1 due to more rapid and efficient cellular entry, independent of gangliosides binding [18]. These findings collectively suggest that the interaction between the heavy chain receptor binding domain of BoNT/A (Hc/A) and the receptor SV2 is crucial in determining BoNT/A potency.

In fact, the entry of BoNT/A into target cells begins with its binding to gangliosides on the cell surface, allowing the toxin to accumulate on the cell membrane before being recognized by protein receptors. Since widely distributed gangliosides are receptors of low affinity, protein receptors are considered the specific receptors for the toxin with high affinity [19]. The SV2, as the protein receptor for BoNT/A was first identified by Dong et al. in 2006 [20]. It exists in three isoforms, and the affinity of BoNT/A for the three isoforms follows the order SV2C >> SV2A > SV2B [21]. Glycosylation of specific receptor sites significantly enhances toxin‐receptor affinity, with glycosylation exerting a much greater impact on SV2A and SV2B binding compared to SV2C [20, 22, 23]. Considering the clinical relevance of BoNT/A's action primarily at the neuromuscular junction (NMJ), where SV2C predominates, we focused on SV2C in this study. Additionally, existing crystal structures of BoNT/A (PDB 3BTA) show a butterfly‐like open conformation, in which the Hc/A domains lie in the same plane as the LC and H_N_/A domains, maintaining an overall extended spatial arrangement. In contrast, a recent cryo‐electron microscopy (cryo‐EM) study (PDB 9F3C; PDB 9F2Y) proposes a semi‐closed conformation with folded wings, where the Hc/A domains fold over the H_N_/A domains to form a more compact structure [24, 25]. Therefore, we also determined BoNT/A structure at pH 7.4 with single‐particle cryo‐EM at a resolution of ∼2.85 Å to identify its confirmation, then the potential receptor binding interface and key interaction residues in Hc/A. Subsequently, a saturation mutagenesis libraries targeting the key residues were constructed and screened by the Bacterial Adenylate Cyclase Two‐Hybrid (BACTH) system to identify mutants that can boost BoNT/A affinity to its receptor SV2C. We examined whether the resultant mutants could substantially enhance SNAP25 cleavage capability in cultured cortical neuron. Furthermore, the engineered mutants were subjected to mouse lethality bioassay (MLB), digit abduction scoring (DAS), and toxin diffusion assays, to verify whether they exhibited higher activity, lower systemic toxicity, and increased safety in preclinical models. The engineered BoNT/A mutants could offer promising drug candidates to mitigate the side effects of wild‐type BoNT/A (BoNT/A wt) and provide valuable cues for engineering other therapeutic toxins.

## 2 Results

### 2.1 Cryo‐EM Structure of BoNT/A Determined at 2.85 Å Resolution

**FIGURE 1:**
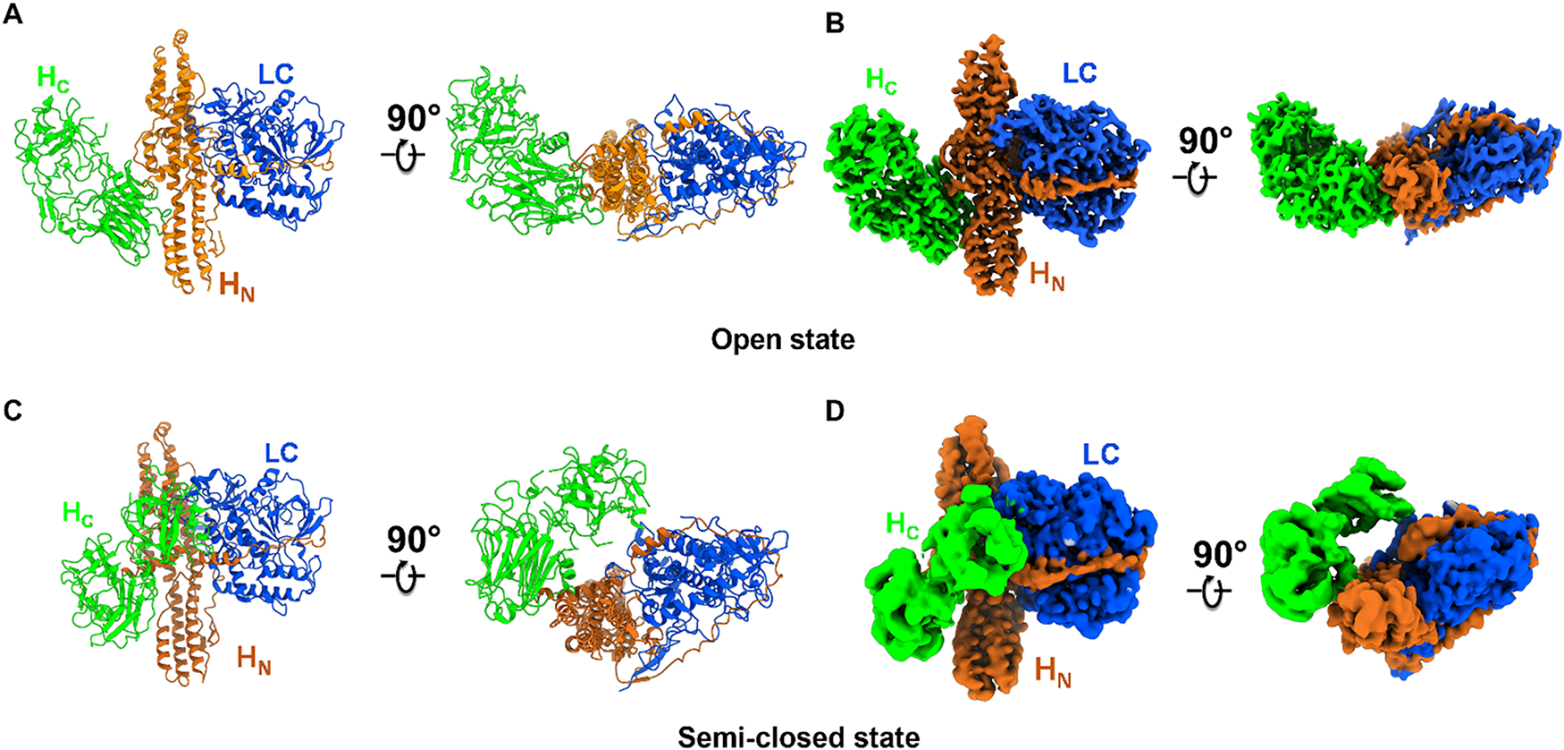
Cryo‐EM structure of BoNT/A holotoxin. (A, B) Cartoon representation and cryo‐EM density map of open‐state BoNT/A. (C, D) Cartoon representation and cryo‐EM density map of semi‐closed‐state BoNT/A. The light chain (LC) is coloured blue, the heavy‐chain H_N_ domain orange, and the heavy‐chain H_C_ domain green.

We determined the structure of full‐length, enzymatic active form of BoNT/A holotoxin at ∼2.85 Å resolution using cryo‐EM single‐particle reconstruction (Figure 1; Figures S1, S2 and Table S1). Consistent with the previous reported crystal structure of BoNT/A, the three domains of light chain (LC), N‐terminus of heavy chain (H_N_/A), and C‐terminus of heavy chain (Hc/A) were clearly identified from the cryo‐EM density map and present a linear arrangement as the crystal structure (Figure 1A,B, PDB 3BTA) [25]. The translocation domain H_N_/A was a central feature consisting of two long (∼100 Å) and several short α‐helices. It wrapped around LC and tightly interlocked the two domains together, thereby stabilizing their interaction and potentially acting as a protective chaperone for the holotoxin [26]. The binding domain Hc/A was primarily composed of two large β‐strands and several short α‐helices, connecting to the middle of the H_N_/A.

Although the cryo‐EM structure of BoNT/A showed nearly the same overall architecture as the previously reported crystal structure with a relatively low root mean square deviations of 1.1 Å, structural variations could be observed from the overall movement of three domains with respect to each other and from the changes in local secondary structures. Structural superposition of the LC domains showed that the heavy chain of cryo‐EM structure shifted ∼ 1.5 Å and rotated ∼3 degrees relative to those in the crystal structure, resulting in a more bent overall architecture (Figure 1A,B). Notably, the two long α‐helices of H_N_/A showed 2 Å shifts at their far ends, with residues T395‐N402 and M646‐D651 adopted significantly different orientations from those in the crystal structure.

**FIGURE 2:**
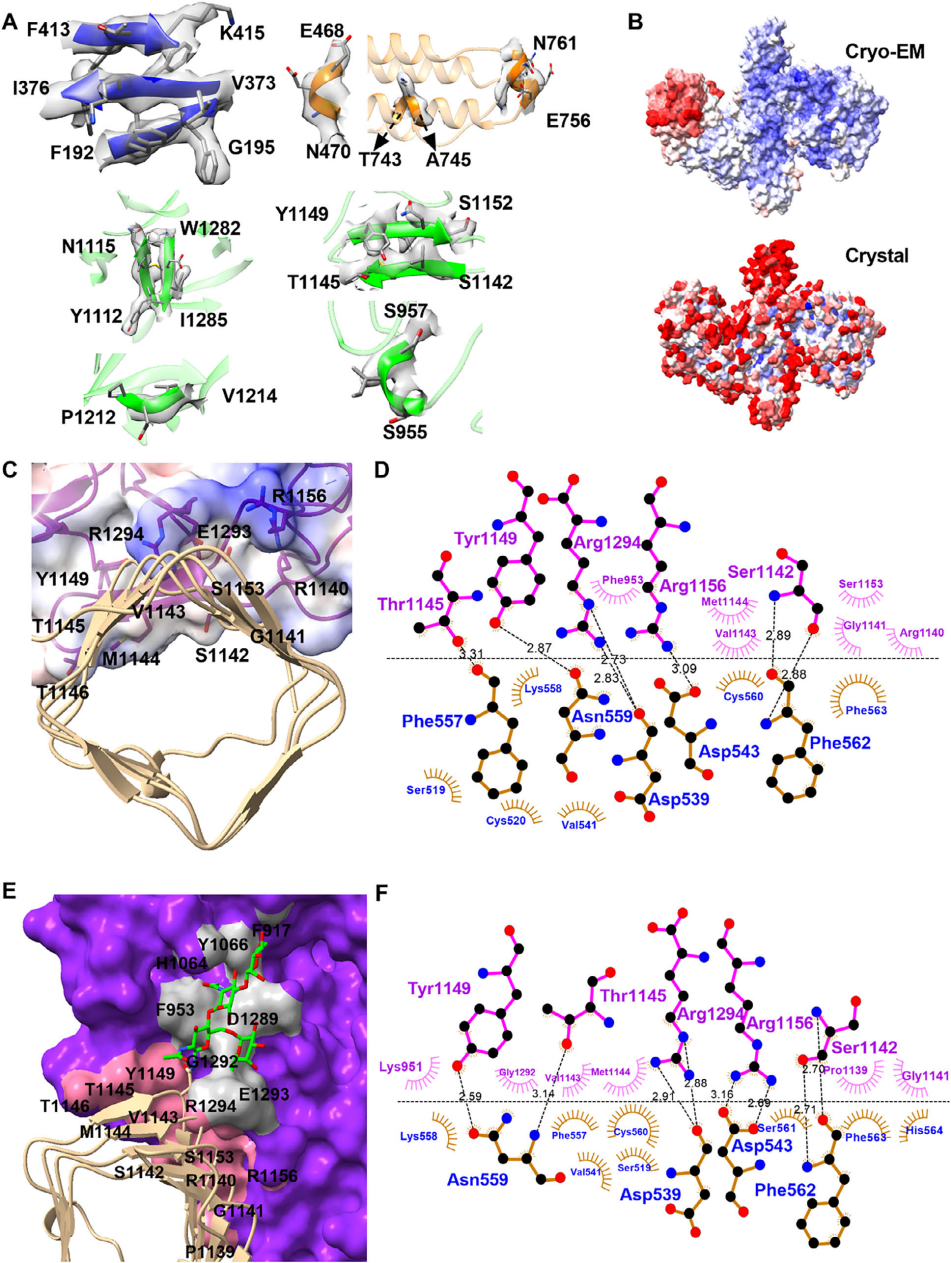
Interaction between BoNT/A and SV2C. (A) The regions identified different secondary structure in LC, H_N_/A, and Hc/A. (B) The cryo‐EM and crystal structures of BoNT/A are colored by using b‐factor, with the high b‐factor region in red and low b‐factor region in blue. (C) BoNT/A is shown in the surface model, with positively and negatively charged residues colored in blue and red, respectively. (D) BoNT/A and SV2C residues are labeled purple and yellow, respectively. The hydrogen bonds between BoNT/A and SV2C are shown as black dot line. Residues involved in hydrophobic interactions are represented by an arc with spokes radiating toward the binding partners they contact. (E) Close‐up views of the protein‐protein and protein‐glycan association interfaces between BoNT/A and glycosylated SV2C. BoNT/A is in surface representation (purple), whereas Hc/A residues that directly interact with the peptide moiety of glycosylated SV2C or the N559 glycan are colored hot pink and gray, respectively. The N‐linked glycan attached to glycosylated N559 of SV2C is rendered in green. (F) The interaction between glycosylated SV2C and BoNT/A. BoNT/A and glycosylated SV2C residues are labeled purple and yellow, the hydrogen bonds between BoNT/A and glycosylated SV2C are shown as black dot line. Residues involved in hydrophobic interactions are represented by an arc with spokes radiating toward the binding partners they contact. The plots were generated using LIGPLOT (Laskowski, R. A. and Swindells, M. B., J Chem Inf Model. 2011;51(10):2778‐86).

Multiple new secondary structures were observed in the cryo‐EM structure. In the LC domain, residues F192‐G195, V373‐I376, and F413‐K415 formed a β‐sheet, which were loops in the crystal structure (PDB 3BTA) (Figure 2A). In the H_N_/A domain, residues E468‐N470, T743‐A745, and E756‐N761 formed alpha‐helices, while they were loops in the crystal structure (Figure 2A). In the Hc/A domain, residues Y1112‐N1115 and W1282‐I1285 were in β‐sheets, and residues S1142‐T1145 and Y1149‐S1152 were in β‐sheets, while residues S955‐S957 and P1212‐V1214 were in alpha‐helices. These secondary structures have not been observed in the crystal structure, suggesting higher structural stability of the corresponding regions under physiological conditions (Figure 2A).

When compared to the previously reported cryo‐EM structure (PDB 9F2B), the open‐state conformation determined in this study exhibits an RMSD of 1.59 Å for the protein backbone, with notable differences observed in the relative positions of the three domains. Upon alignment via the light chain (LC), the heavy chain shows a positional shift of 1–2 Å. In particular, the spatial orientations of the two long ɑ‐helices within the H_N_/A domain differ substantially (Figure S3).

In addition to the open conformation, a semi‐closed conformation of BoNT/A was also resolved from the same cryo‐EM sample (Figure 1C,D; Figures S1, S2C,D and Table S1). Its overall structure is similar to the previously reported semi‐closed cryo‐EM structure (PDB 9F2Y; RMSD 1.2 Å), while showing observable differences in the spatial arrangement of the three domains. Notably, the receptor‐binding domain (RBD) undergoes a conformational shift beginning at residue I870 (Figure S3).

### 2.2 Key Residues of BoNT/A Interacting With SV2C Revealed by Structural Analyses

Previously reported crystal structure of BoNT/A‐SV2C complex (PDB 4JRA) shows that BoNT/A binds to SV2C with its Hc/A domain, which undergoes observable conformational change after SV2C binding [27]. This result highlights the local mobility of Hc/A domain. Consistently, the Hc/A domain showed the highest b‐factor in the cryo‐EM structure, demonstrating its high flexibility (Figure 2B). This phenomenon was not observed in the crystal structure, confirming that the cryo‐EM structure presented a more native structure of BoNT/A.

To gain insights into the binding of native BoNT/A with SV2C, we calculated the binding probability of each residue of BoNT/A with SV2C by ScanNet [28], and predicted the BoNT/A‐SV2C complex structure based on the resolved cryo‐EM structure of BoNT/A. The residues S1142, M1144, and T1146 of BoNT/A showed the highest binding probability to SV2C. The Hc/A domain of cryo‐EM structure was superposed onto the Hc/A‐SV2C crystal structure (PDB 4JRA) [27], and the resulting BoNT/A‐SV2C complex structure was further refined through energy minimization (Figure 2C). One prominent feature seen in the structure was the accumulation of positively charged surface residues at the Hc/A interface, such as residues R1156 and R1294 (Figure 2C). These residues likely contributed to the shape and charge complementarity to the negatively charged SV2C surface. The β‐strand edge of BoNT/A interacted with the open β‐strand of SV2C, with K951, F953, P1139, R1140, G1141, S1142, V1143, M1144, T1145, T1146, Y1149, S1153, R1156, G1292, E1293, and R1294 within 4 Å of SV2C (Figure 2C,E). Among those resides, S1142, T1145, Y1149, R1156 and R1294 of BoNT/A formed hydrogen bonds with SV2C, suggesting their critical roles in BoNT/A‐SV2C binding (Table S2 and Figure 2D). Critically, residue N559, which is the N‐glycan site at the conserved luminal domain of SV2C formed two hydrogen bonds with BoNT/A (residues T1145 and Y1149) (Figure 2F). A similar pattern of hydrogen bond involving the corresponding N‐glycosylation sites (N573 in SV2A, and N516 in SV2B) could be observed in our predicted BoNT/A‐SV2A and BoNT/A‐SV2B complex models (Table S3), with each complex exhibiting multiple hydrogen bonds. These results strongly supported the pivotal role of the N‐glycan at SV2C N559 in BoNT/A‐SV2C binding, and were consistent with prior studies highlighting the importance of conserved glycosylation at Asn residue across SV2 isoforms for BoNT/A interaction [20, 22, 23]. Compared to previous work [22], it is noteworthy that M1144 was not observed to form a hydrogen bond with SV2C glycosylated or not (Figure 2D,F), suggesting that the site is more modifiable.

### 2.3 BACTH Assay Screening Identified S1142, M1144 and T1146 as Flexible Sites Subjected to Change Toxin‐Receptor Binding Affinity

**FIGURE 3:**
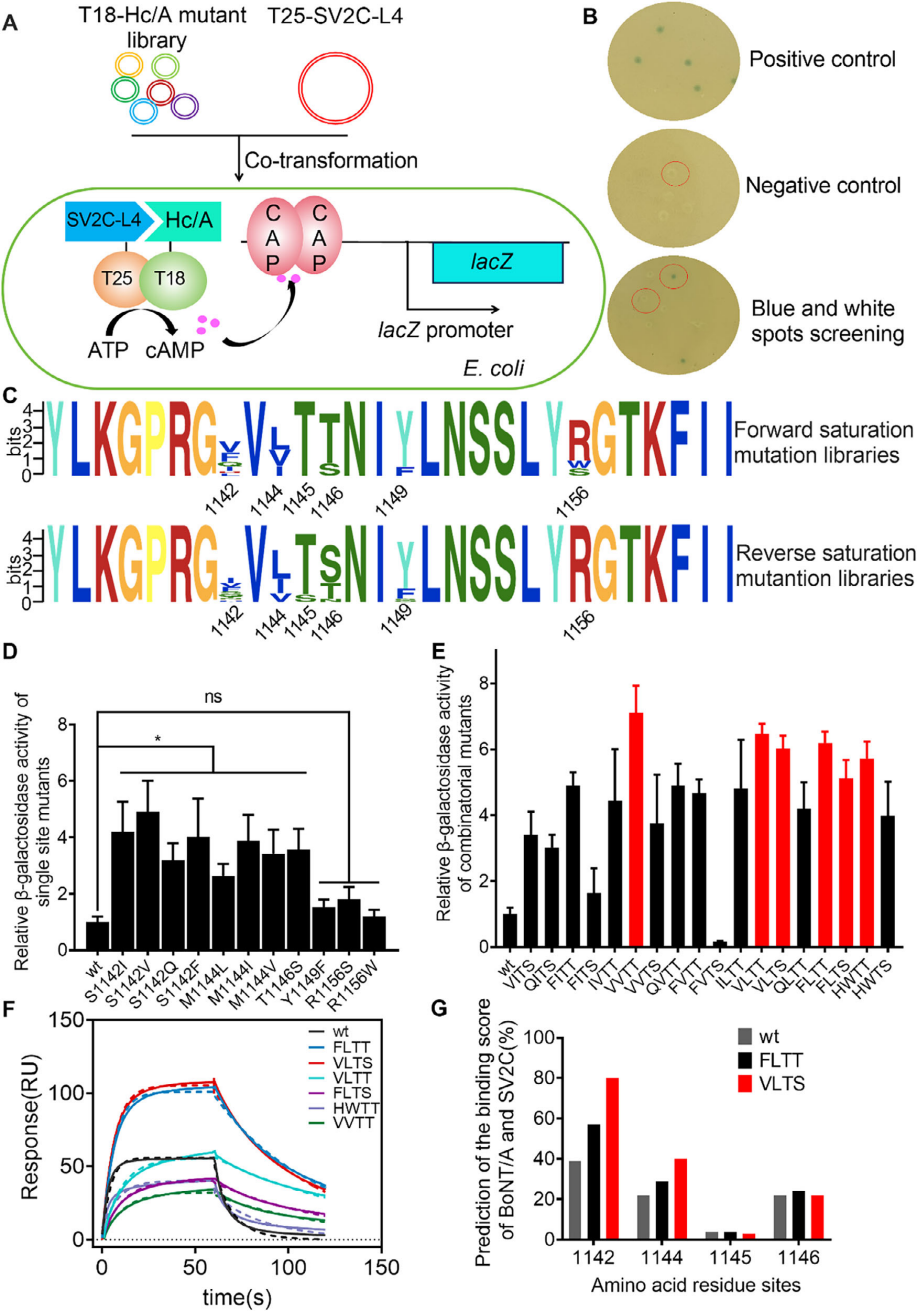
BACTH screen identified mutations that increase BoNT/A binding to SV2C. (A) A schematic illustration for the BACTH assay. (B) The blue and white colonies on the screening plates. In this case, all positive controls occurred as blue spots, all negative controls exhibited as white spots, and the experimental group contains both blue and white spots (*n* = 3). (C) Results of screening forward and reverse saturation mutant libraries by the BACTH assay. (D) Hc/A single point mutants identified by using the BACTH system were further analyzed by β‐galactosidase activity assay. *n* = 4, *p < 0.05, ns *p* > 0.05. Error bars represent SD. (E) Hc/A combination mutants identified by using the BACTH system were further analyzed by β‐galactosidase activity assay. Statistical significance was determined using ordinary one‐way ANOVA with Dunnett's post hoc test for comparison to the control group in (D) and (E). There were significant differences between Hc/A wt and all combined mutations at a level of *p *< 0.05 except FITS, *n* = 4. Error bars represent SD. The red bars corresponded to the mutants displaying the best interaction. (F) The binding affinities and kinetic parameters of Hc/A wt and combined mutations to GST‐SV2C‐L4 determined by SPR. Representative association and dissociation curves are shown for Hc/A wt and six Hc/A mutations. SPR sensorgrams of Hc/A binding to GST‐SV2C‐L4 (solid line) overlaid with a fit of 1:1 binding model (dashed line). (G) The binding probability of the four amino acid sites with SV2C for BoNT/A wt, FLTT and VLTS mutants predicted by ScanNet.

BoNT/A residues showing high binding probability or critical interactions with SV2C in the above ScanNet and docking analyses were selected for further mutagenesis. These residues included 1144, 1146 (high binding probability), 1145, 1149 (direct hydrogen bonds), 1142 (high binding probability and hydrogen bonds), and 1156 (shape and charge complementarity via electrostatic surface analysis). Our strategy was to replace each of these positions with all 20 possible amino acids to identify all single‐residue mutations that increased binding to SV2C by the BACTH method (Figure 3A). The Hc/A was subcloned into a vector with the T18 fragment of bacterial adenylate cyclase to construct T18‐Hc/A, and SV2C‐L4 was subcloned into a vector with the T25 fragment of bacterial adenylate cyclase to construct T25‐SV2C‐L4. To create a mutation library for further selection, T18‐Hc/A wt was amplified by PCR using primers with NNK to generate a pool of mutations encoding all 20 possible amino acids at the codons of interest residues. The mutation libraries were then co‐transformed into competent bacteria *Escherichia coli (E. coli)* BTH101 together with T25‐SV2C‐L4. The binding of Hc/A protein to SV2C‐L4 will restore the adenylate cyclase activity by combining T18 and T25, which complement each other to produce cAMP, thereby promoting the expression of *lac*Z gene and finally the formation of blue colonies on X‐Gal plates (Figure 3B). The BACTH system links the increased toxin‐receptor binding activity of Hc/A mutations to the increased β‐galactosidase activity with bluer colonies as readout. In the PCR reaction system for site‐directed mutagenesis, the template of T18‐Hc/A wt was designed to be subsequently cleaved by DpnI, which acts only when the adenine residues are methylated. However, it is possible that DpnI did not completely digest the template of T18‐Hc/A wt in the PCR system, resulting in false negative results. To exclude the interference of T18‐Hc/A wt that might not be completely excised by *DpnI* enzyme during the screening, we picked receptor‐binding inactivation mutants of white colonies from the plates validated by Sanger sequencing. The resultant T18‐Hc/A mutant was then used as a template for PCR with NNK primers to construct expected mutation pools. We named these pools as reverse saturation mutation libraries. In contrast, the early pools with T18‐Hc/A wt as template were named as forward saturation mutation libraries.

After screening the forward and reverse saturation mutation libraries, we identified that positions at S1142, M1144, and T1146 were more flexible sites for toxin‐SV2C‐L4 binding, with more amino acid substitutions at position S1142 dominated by V/F/Q/I, then M1144 dominated by L/V/I, and T1146 dominated by T/S. While T1145, Y1149, R1156, and R1294 sites almost remained as wild type across both forward and reverse saturation mutagenesis screens (Figure 3C).

The β‐galactosidase activities of the mutants S1142I/V/Q/F, M1144L/I/V, T1146S, Y1149F and R1156S/W were subjected to further analysis. The results demonstrated that the mutants S1142I/V/Q/F, M1144L/I/V, and T1146S exhibited enhanced β‐galactosidase activity compared to the wild‐type counterpart colony, suggesting that these mutations have an enhanced binding affinity to the receptor SV2C (Figure 3D).

### 2.4 Engineered BoNT/a Heavy Chain With Combinational Mutations Had a Boosted SV2C Binding Affinity

The residues S1142, M1144, and T1146 of Hc/A were selected for further combined mutation screen again by the BACTH method for systematically mutated into the 20 natural amino acids. Although the single‐site mutagenesis screening results showed that T1145 was recognized as unmodifiable, we still incorporated it as a screening control for the combined mutagenesis screening for further verification. Totally combined mutation libraries of S1142/M1144, T1145/T1146, and S1142/M1144/T1145/T1146 were constructed by PCR using primers with NNK or MNN, and then subjected to both the forward screen and the reverse screen. The combinational mutants FLTT, VLTS, IVTT, VVTT, QVTT, FITT, and HWTT were identified by sequencing many bluer colonies. As expected, all the mutants obtained through the combined screening again have a T at position 1145. Further, those combinational mutations and several other mutations were also selected for the β‐galactosidase activity assay. The results showed that almost all of the mutants had significantly increased β‐galactosidase activity, and VVTT, VLTT, VLTS, FLTT, FLTS, and HWTT mutants ranked among the top (Figure 3E).

**FIGURE 4:**
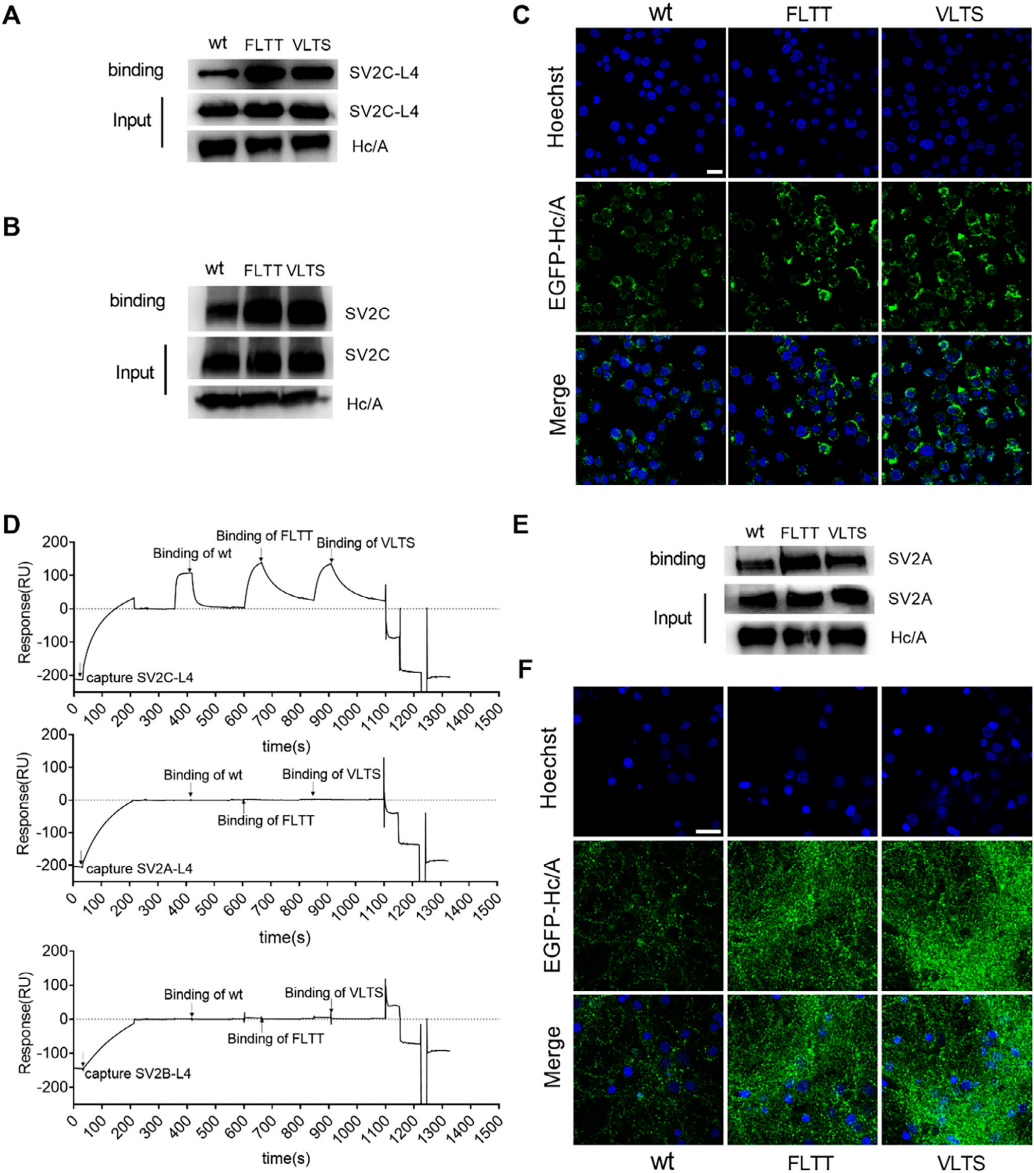
Hc/A mutants FLTT and VLTS showed higher binding ability with SV2s than Hc/A wt. (A) The binding of GST‐SV2C‐L4 to immobilized Hc/A wt, VLTS, and FLTT on Ni^2+^‐NTA beads by pull down assay. (B) The binding of glycosylated SV2C to immobilized Hc/A wt, VLTS, and FLTT on Ni^2+^‐NTA beads from rat brain lysate by pull‐down assay. (C) The binding of EGFP‐Hc/A wt, mutants of EGFP‐FLTT, and EGFP‐VLTS to the surface of Neuro‐2a cell lines, respectively. Scale bar 10 µm. (D) The binding affinity tests of Hc/A wt, VLTS, and FLTT to SV2s were performed by SPR assay (*n* = 3). (E)The binding of SV2A to immobilized Hc/A wt, VLTS and FLTT on Ni^2+^‐NTA beads from cultured neurons by pull‐down assay. (F) The binding of EGFP‐Hc/A wt and the variants (100 nmol) to the surface of rat primary cultured hippocampal neurons. Scale bar 10 µm.

The toxin‐receptor affinities of these six combinational mutations were examined by surface plasmon resonance (SPR). The Hc/A wt, combinational mutation Hc/A proteins, and GST‐SV2C‐L4 fusion protein were purified (Figure S4). The kinetic curves of these mutants and wt for SV2C‐L4 were shown in Figure 3F and Figure S5, the binding parameters were listed in Table S4. Briefly, GST‐tagged SV2C‐L4 proteins were coupled to the microarray, sequentially followed by flow through the chip surface at a flow rate of 30 µL/min with varying concentrations of Hc/A protein ligands as the binding phase, then by a washing step as the dissociation phase. The toxin‐SV2C‐L4 binding affinity (KD) of Hc/A wt served as a control at a level of 92.9 nmol, and the KD values of all combinational mutants ranged from 14.2 to 52.9 nmol. Among the combined mutants, FLTT and VLTS had higher toxin‐SV2C‐L4 binding affinities than wild type by 6.6‐fold and 6.0‐fold, respectively (Table S4). Consistently, the predicted binding probabilities of BoNT/A wt and mutants FLTT and VLTS with SV2C indicated that each residue in mutant VLTS nearly showed the highest binding probability to SV2C (Figure 3G). From a structural perspective, the combinational mutations FLTT and VLTS strengthen the hydrophilic and hydrophobic interactions at the interface between BoNT/A and SV2C, specifically between residues 1142, 1144, 1145, 1146 of BoNT/A and residues 557–563 of SV2C (Figure 3G). As expected, Hc/A combined mutants FLTT and VLTS as bait proteins could pull down more prey SV2C‐L4 than Hc/A wt, confirming the enhanced binding affinity indeed (Figure 4A). Considering the N‐glycan at the conserved Asn residue in luminal domain 4 of SV2C further strengthens the toxin's binding to the receptor itself [21, 23, 24]. We performed pull‐down using Hc/A as bait from rat brain lysates, which express highly glycosylated SV2 (Figure 4B). The result confirmed that FLTT and VLTS strengthened the binding affinity to N‐glycosylated SV2 at the tissue or organ level. The result confirmed FLTT and VLTS also strengthened the binding affinity to N‐glycosylated SV2C in eukaryotic cells. Further, EGFP‐Hc/A mutants exhibited stronger binding to the surface of Neuro‐2a cells, which expressed mostly SV2C [29], and were ranked VLTS > FLTT >wt by confocal microscopy (Figure 4C and Figure S6A). This is consistent with the results of Mahrhold et al., suggesting that the glycosylation modification of SV2A and SV2B is crucial for their binding with BoNT/A. EGFP‐Hc/A wt and mutants showed obvious binding to the cell surface of Neuro‐2a cells, and with further 3D imaging analysis, it was observed that they could also be internalized inside the cells (Figure S6B). The toxin receptor SV2 protein exists in three isoforms A‐C, and these SV2 isoforms are differently expressed in different neuronal systems and neuron types [30]. Of which, SV2A and SV2B play a significant role in BoNT/A binding and uptake [31]. In the present work, we recombined the SV2A (residues Y466‐V598) and SV2B (residues Y409‐M535), and performed a SPR assay on the binding affinities of SV2A and SV2B to Hc/A wt or mutants. The result showed minimal binding capacity between these two SV2 isoforms and Hc/A VLTS or FLTT (Figure 4D). This result was supported by a previous report that BoNT/A binding to non‐glycosylated SV2C‐LD4 expressed in *E. coli* was much stronger than to non‐glycosylated SV2A/B [32]. When functional potency assays were conducted in rat hippocampal neurons, where glycosylated SV2A/B demonstrates high expression, while SV2C shows negligible expression, the binding affinity of EGFP‐Hc/A mutants was markedly higher than that of wt by fluorescence microscopy and pull‐down assay (Figure 4E,F). It has been reported that SV2C is the dominant receptor isoform at motor neurons in the NMJ [33]. Therefore, we still focused on the most clinically relevant aspect of the binding capacity between SV2C and Hc/A mutants.

### 2.5 Full‐Length Engineered BoNT/A Mutants Showed Improved SNAP25 Endopeptidase Efficiency in Neurons and Advanced Muscle Paralysis Efficacy in Animals

**FIGURE 5:**
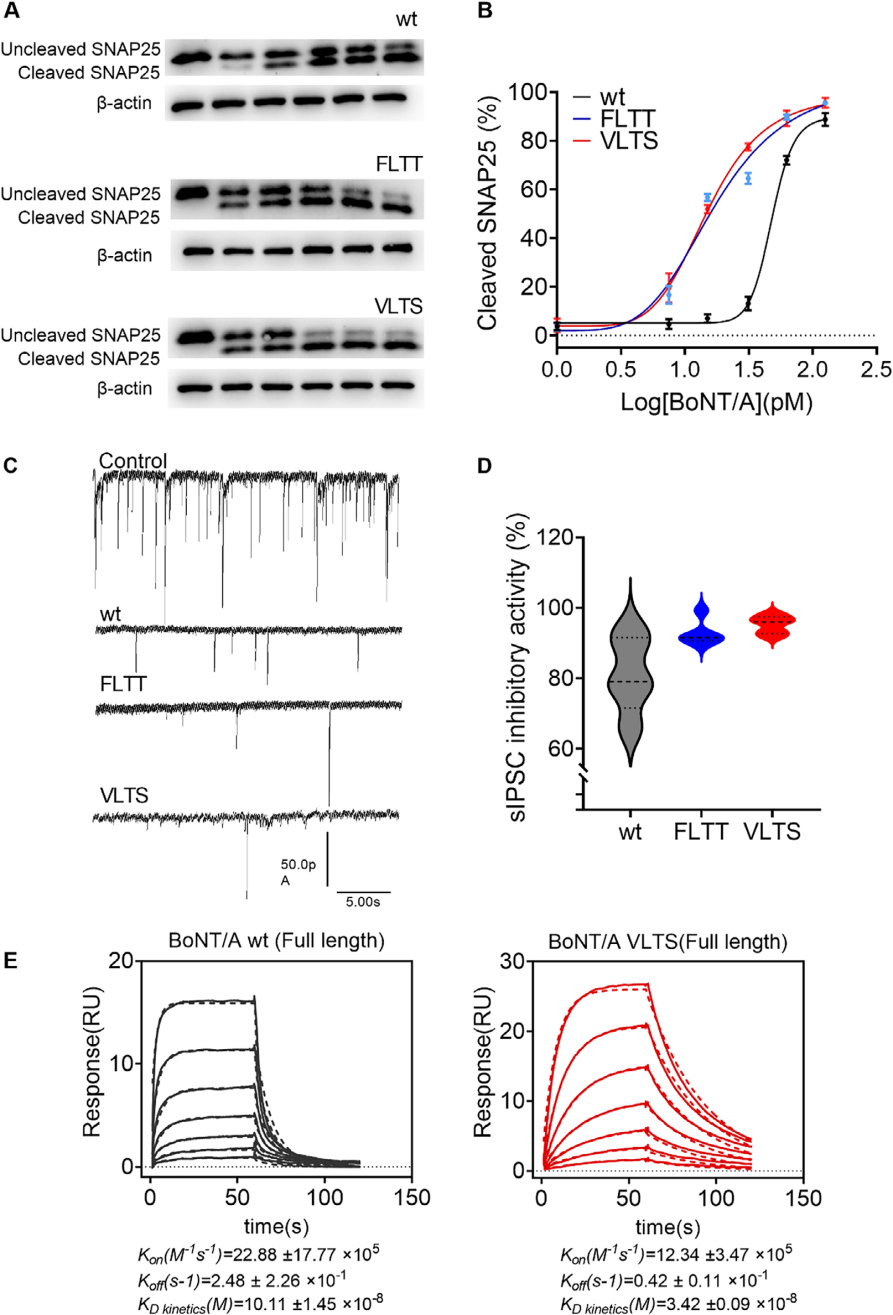
Hc/A mutants FLTT and VLTS showed higher binding ability with SV2s than Hc/A wt. (A) FLTT and VLTS had greater SNAP25 cleavage activity than BoNT/A wt in cultured rat cortical neurons. Neuron cells were exposed to full‐length BoNT/A wt, FLTT, or VLTS at a series of concentrations of 0, 7.5, 15, 31.25, 62.5, and 125 pmol for 48 h. Cell lysates were harvested and analyzed by immunoblotting. β‐actin was used as an internal control, and the black arrow from head to tail showed as toxin concentration increase. (B) Quantification results of (A) by Image J software. Error bars show standard deviation (*n* = 3). (C) Representative sIPSC recordings of neurons treated with BoNT/A wt and engineered BoNT/A FLTT and VLTS at 31.25 pmol, respectively. Cortical neurons without exposure to toxins were used as control. (D)The sIPSC inhibition capability of BoNT/A wt and engineered BoNT/A FLTT and VLTS at 31.25 pmol. The data was normalized to recordings that of without exposure to toxin. (E) Characterizing full length BoNT/A or mutant VLTS binding to SV2C‐L4 using the SPR assay. Microarrays containing immobilized GST‐SV2C‐L4 were exposed to the indicated concentrations of Hc/A, full length BoNT/A or VLTS to determine the binding kinetics. SPR sensorgrams of Hc/A binding to GST‐SV2C‐L4 (solid line) overlaid with a fit of 1:1 binding model (dashed line) (*n* = 3).

We tested whether Hc/A combinational mutants with increased binding affinity to SV2C‐L4 harbored higher functional potency. For this purpose, recombinant full‐length BoNT/A wt and BoNT/A mutants containing FLTT and VLTS in Hc/A were produced in *E. coli* BL21(DE3). The activated BoNT/A showed a heavy chain of ∼100 kDa and a light chain of ∼50 kDa separated in reduced form, while ∼150 kDa in the non‐reduced form (Figure S7). The binding affinity capacities and SNAP25 cleavage activities of BoNT/A mutants were then compared with that of BoNT/A wt in cultured rat cortical neurons. The BoNT/A mutation FLTT and VLTS cleaved SNAP25 more efficiently than BoNT/A wt (Figure 5A,B; the EC_50_ values of wt, FLTT, and VLTS were 48.14, 16.21, 14.77 pmol, respectively). Again, the BoNT/A mutation VLTS cleaved SNAP25 with a three‐fold higher efficiency compared to BoNT/A wt. Once SNARE complex is cleaved by BoNT/A, neurotransmitter release activity will be markedly suppressed, which could be reflected by the frequency of spontaneous inhibitory postsynaptic currents (sIPSC) [15]. Here, sIPSCs of the cortical neuronal cell population were recorded by whole‐cell patch‐clamp. The results showed that the sIPSC activities were more efficiently blocked by FLTT and VLTS mutants than BoNT/A wt (Figure 5C,D). We noticed that VLTS tended to have higher efficacy than FLTT, the receptor‐binding affinity of the full‐length mutant toxin was focused on. SPR assays showed that the KD value of BoNT/A mutation VLTS increased by about three‐fold than that of BoNT/A wt (Figure 5E). Notably, the thermal stability of the BoNT/A VLTS mutant and the wild‐type protein was assessed by differential scanning fluorimetry (DSF). Their melting temperatures (Tm) were nearly identical, with values of 55.2°C ± 0.05°C for the wild‐type and 55.4°C ± 0.05°C for the VLTS (Figure S8).

**FIGURE 6:**
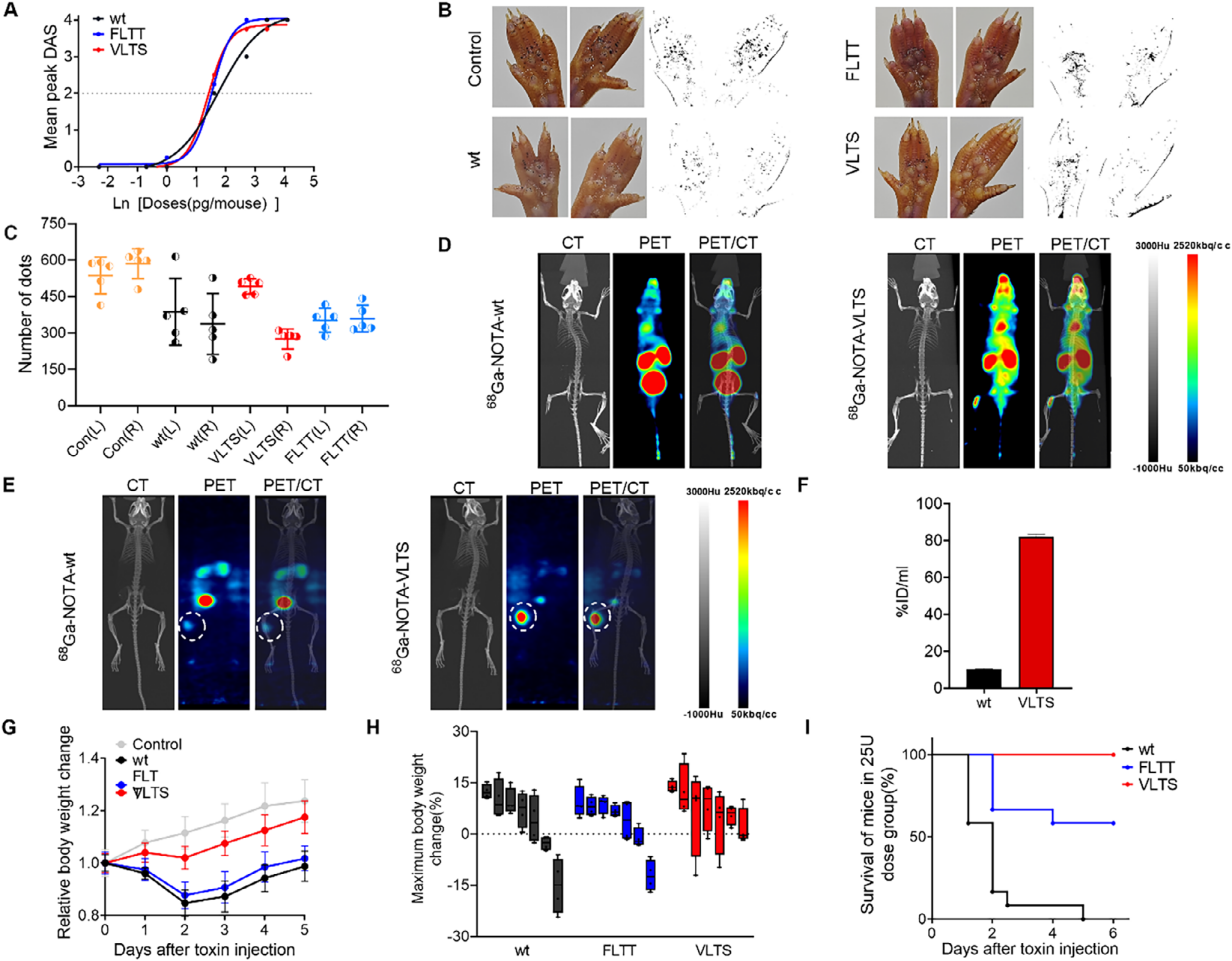
Engineered BoNT/A with FLTT and VLTS mutations exhibited lower diffusivity and lethality than BoNT/A wt. (A) Concentration response curve in DAS assay in mice. Points are peak mean DAS scores for each dose group, using four animals per dose (*n* = 4). (B) Starch‐iodine sweat test of full length BoNT/A wt and mutants. The right paws of mice were injected toxins, and the left paws were left untreated. The right paws of mice in control group were injected PBS containing 0.25% BSA. (C) Quantification results of (B) by Image J software. Error bars show standard deviation (*n* = 5). (D) Mice were intravenously injected with 200 µCi ^68^Ga‐NOTA‐Hc/A wt or VLTS (100 µg of NOTA‐Hc/A wt or VLTS). (E) Mice were intramuscularly injected with 6.5 µCi ^68^Ga ‐NOTA‐Hc/A wt or VLTS (2.5 µg of NOTA‐ Hc/A wt or VLTS). The white dotted circle indicates intramuscular injection site. (F) Region of interest (ROI) analysis of (E) (*n* = 3). (G) Relative body weight changes in mice were recorded for consecutive five days after injected with 60 pg full length toxins into the gastrocnemius muscle of the left hind limb. Relative body weight of animal individuals was calculated by the BW obtained from daily weighing divided by the BW at the first day of the experiment (*n* = 4). (H) Maximum body weight change (%) of mice in each dose group for three toxins. The injection doses of animals in each group were 0.25, 0.5, 1, 5, 15, 30, 60 pg. Each box represents the percentage change in body weight relative to pre‐injection weight of mice in each dose group of three toxins. Box plot was derived from data of the most severe weight loss percentage relative to pre‐injection weight of mice on the second day (*n* = 4). (I) Survival curves of mice after intramuscularly injected with 25 U toxins. The ED_50_ value of each toxin was defined as 1U (*n* = 4).

We further investigated whether Hc/A mutations improved the cleavage activities of SNAP25 in cultured cortical neuron can translate into increased functional potency in intact animals. The DAS test in mice is widely used to represent the biological activities of BoNT/A due to its clinical relevance [34]. We evaluated the functional potency of BoNT/A wt and mutations FLTT and VLTS in vivo by DAS assay. Injection of a sublethal concentration of the toxin into the gastrocnemius muscle of the left hind limb of mice induces muscle‐relaxant paralysis and prevents toe abduction. The degree of paralysis is set on a 5‐point scale from normal digital abduction responses (DAS 0) to full inhibition of digital abduction responses (DAS 4) [34]. Toxins induced DAS was recorded from the start of injection at 24 h intervals until mice toe abduction turned to complete recovery (Figure S9). The median effective dose (ED_50_) was calculated from the mean peak DAS score fitting curves against each dose on the natural logarithm scale, by which the toxin dose that induced a DAS value of 2, and then inverting to its linear value (Figure 6A). The ED_50_ showed that the BoNT/A mutants VLTS and FLTT were superior to the wild‐type counterpart (Table 1). The data suggested that engineered BoNT/A FLTT and VLTS resulted in a higher functional potency of muscle paralysis than wild type, with VLTS slightly more effective than FLTT.

**TABLE 1:** Parameters for evaluating the effectiveness and safety of engineered BoNT/A toxins in vivo. Abbreviations: BoNT, botulinum neurotoxin; %BW, the maximum dose of toxin that induces no changes in mouse body weight; ED_50_, median effective dose; IP LD_50_, Intraperitoneal median lethal dose; IM LD_50_, intramuscular median lethal dose. The toxin content was converted based on 1.63 A_278_ = 1 mg/mL to enable easier comparison of results from different studies (DasGupta BR and Sathyamoorthy, Toxicon. 1984;22(3):415‐424).

BoNT/A	ED_50_ (pg)	0%BW (pg)	0%BW/ED_50_	IP LD_50_	IM LD_50_
wt	3.53	14.97	4.24	12.88	65.77
FLTT	2.86	15.70	5.49	20.36	68.96
VLTS	2.48	40.39	16.25	20.36	103.50

### 2.6 The Engineered BoNT/A VLTS Exhibited Lower Toxicity and Better Safety Profiles in Preclinical Animal Models

A key challenge for BoNT/A clinical use is the spread to other regions beyond the injection site. So, we investigated whether engineered BoNT/A with higher binding affinity to its receptor SV2C could significantly remedy this problem. BoNT/A has been applied tremendously to treat hyperhidrosis clinically [35, 36]. A pharmacological relevant sweat test model was constructed to evaluate the local and systemic diffusion of BoNT/A wt, engineered BoNT/A mutants FLTT and VLTS in mice. The locally injected toxin might diffuse from the injection site in the right paw into the left paw, which could induce the inactivation of sweat glands in bilateral footpads. The result showed that the engineered toxin VLTS exhibited advanced efficacy and less diffusion than BoNT/A wt (Figure 6B,C). PET/CT imaging was performed to observe the distribution of ^68^Ga labeled Hc/A wt and VLTS after intravenous injection and intramuscular injection (Figure S10). Hc/A distributed on the head, neck, spine, heart, kidney, bladder, knee joints in mice after intravenous injection, it is due to the migrations of Hc/A with bloodstream, and retained in those tissues or organs where the SV2 receptors enriched (Figure 6D and Figure S11A). The result also showed VLTS bound much strongly to the receptors than Hc/A wt. To further evaluate the clinical relevance, local application was carried out and confirmed that VLTS remained significantly more concentrated at the injection site compared to Hc/A wt (Figure 6E,F and Figure S11B). This enhanced retention reduced systemic diffusion and potential side effects.

We hypothesized that the lower diffusivity of engineered mutants VLTS can give lower toxicity than BoNT/A wt. In the DAS test, different degrees of body weight (BW) loss were observed in mice injected with high doses of the three toxins, which was explained by systemic toxicity due to the diffusion of the toxin from the injection site [37]. The extent of the BW loss was positively correlated with the toxin dose. The mice at higher dose experienced a more pronounced reduction in BW gain than those at the lower dose, and the most severe BW loss was observed in BoNT/A wt, followed by the FLTT, and to a marked lesser extent, the VLTS (Figure 6G,H). The tolerability index of 0% BW/ED_50_ equals to the maximum dose of toxin that induces no changes in body weight (%BW) divided by ED_50_, representing a safety margin that the administrated toxin is most likely restricted to the injection site without spreading systemically [38]. Here, although the ED_50_ values for FLTT and VLTS were slightly lower than for BoNT/A wt, the tolerability index for VLTS showed much larger than both FLTT and BoNT/A wt (Table 1).

The lethality and diffusivity of the engineered toxins were further assessed by the MLB assay. The lethality of the three toxins was evaluated by using indices of median intramuscular lethal dose (IM LD_50_) and median intraperitoneal lethal dose (IP LD_50_). IM LD_50_ value for VLTS was much higher than BoNT/A wt and FLTT, while IP LD_50_ values for FLTT and VLTS were both significantly higher that BoNT/A wt (Table 1). These data, consistent with the sweat test findings, confirmed that engineered BoNT/A VLTS diffused significantly less than both wt and FLTT. This reduced diffusion results in its lower toxicity and lethality. The ED_50_ value is defined as one unit of toxin, and 25 U of BoNT/A wt and engineered toxins were injected intramuscularly in mice. Indeed, the survival analysis showed that engineered BoNT/A VLTS ranked first, then FLTT, and followed by BoNT/A wt on the respect of safety (Figure 6I).

In our present work, the engineered BoNT/A VLTS and FLTT with higher affinity of SV2C receptor binding demonstrated higher locally efficacy, as evidenced by the lower ED_50_ for intramuscular injection. However, higher efficacy locally does not necessarily cause higher toxicity measured by lethality, instead, as shown by the results, the values of IM LD_50_ and IP LD_50_ increased. This phenomenon was similarly observed in BoNT/A6, which has a leucine at site 1144 and also shows an increased IM LD_50_ [39]. It can be attributed to the local efficacy and systemic toxicity of BoNT/A. The toxin impairs nerve function and causes muscle paralysis. Once it enters the bloodstream and affects the respiratory muscles, respiratory failure is considered the usual cause of death [40]. We introduced mutations at modifiable amino acid residues within the toxin's interaction interface with its receptor. Compared to wild‐type BoNT/A, this engineered variant adopts a more tightly binding form between BoNT/A and SV2C. So the binding affinity of BoNT/A binding domain with SV2C increased by up to approximately six‐fold, thereby strengthening the toxin's binding to the targeted cell surface. As for why VLTS and FLTT displayed similar behaviors in vitro but differed in vivo, the underlying reason requires further experimental investigation. Intraperitoneal injection of toxins with a higher affinity makes them more likely to be locally captured by the SV2 receptors once the toxin reaches the site. Localized respiratory muscle dysfunction is compensated by other respiratory muscles. Therefore, a higher dose of toxin is required to cause death in mice through distant diffusion. Consequently, the IP LD_50_ of the mutant toxins with higher binding affinity to SV2C is increased, and their intraperitoneal toxicity is reduced.

## 3 Discussion

The BoNT/A structure provides tremendous information for the investigation of receptor binding [27], substrate specificity [39, 41], and enzymatic catalytic activity [25]. In the present work, we determined BoNT/A structure by single particle cryo‐EM at a resolution of ∼2.85 Å, which was more closely to its native physiological status at pH7.4 (Figure 1). Overall, superposition with crystallographic structure resulted in a nearly perfect overlap of the translocation domains, but the heavy chain shifted by ∼1.5 Å and rotated by ∼3 degrees. We identified a new unique β‐sheet structure within the light chain domain, containing residues 373–376 and 413–415 (Figure 2A). These sets of residues formed β‐strand conformation each, and their function might be relevant to substrate recognition and proteolytic activity, remained to be resolved.

It is to be noticed that the detailed cryo‐EM structure of BoNT/A reported by Khanppnavar et al. shows that BoNT/A is in a unique semi‐closed conformation in a nearly physiological neutral condition, at pH of 8.0 [24]. After binding to the receptor, it transfers into an open conformation. And then upon uptake by the target cell, it returns to the original semi‐closed conformation under the acidic conditions of the endosome, where the pH is 5.5. This conformation allows its light chain to approach the membrane, facilitating the translocation of the light chain into the cytoplasm to exert its effect. In contrast, the present study on our cryo‐electron microscopy conformation of BoNT/A showed that semi‐closed conformation and open conformation co‐existed in our sample. According to the open conformation as defined by Khanppnavar et al. [24], we believe that BoNT/A should be in this open conformation but not the semi‐closed confirmation under physiological pH conditions for the following reasons: First, we induced BoNT/A to adopt a semi‐closed conformation at pH 5.5 as Khanppnavar et al. reported, and employed GST‐SV2C‐L4 as the bait in SPR biosensor assay. The results showed that the binding capacity of the ligand and the receptor reduced to a disassociation constant (KD) of 538.6 nmol at pH 5.5 (Figure S5 and Table S4). In comparison, the expected strong binding did occur at pH 7.4, with a KD of 92.9 nmol. This is consistent with the results of Weisemann et al. and Benoit et al., [21, 27], suggesting that BoNT/A‐SV2C complex tends to dissociate under acidic pH, and the conformation of BoNT/A is in pH‐dependent manner. Therefore, the stronger receptor binding activity closely linked to the open form of the toxin at physiological neutral pH. Second, Chellappan et al. [42] showed that the melting temperature (Tm) of BoNT/A is measured by circular dichroism spectroscopy and calculated using the Van't Hoff equation, it is 55.6°C at pH 7.2, up to 71.0°C at pH 4.5. It is indicated that BoNT/A semi‐close conformation is more stable at an acidic pH. The SV2C rigid conformation for binding to the toxin is pre‐existing quadrilateral β sheet helix [21]. The binding to its ligand is more akin to a key‐and‐lock mechanism. If BoNT/A adopts the semi‐close conformation under physiological condition, then how does it overcome the energy barrier to change into an open state when binding with its receptor? Third, the majority of toxin particles exhibited an open conformation as showed, there were only 18% particles in our cryo‐EM reconstruction to be identified to underwent a rotation of more than 100 degrees in H_N_/A domain (Figures S1C, S3). Those particles can be recognized as the semi‐closed toxin proteins resulting from lactic acid accumulation under conditions of insufficient oxygen supply, which generate an acidic microenvironment in host *E. coli* cells.

In the present work, we aimed to engineer BoNT/A to improve its receptor binding affinity, so we focused further on the analysis of Hc/A region of the cryo‐EM structure. The Hc/A region is highly flexible, with the b factor highest in all regions. The highly dynamic structure could benefit from the confirmation changes upon receptor binding. A new β‐sheet structure containing 1112–1115, 1142–1145, 1149–1152, and 1282–1285 residues might form a binding interface for its receptor SV2C (Figure 2A). Based on the cryo‐EM structure docking with SV2C, and the co‐crystal structure of Hc/A and SV2C‐L4 [27], potential interaction residues were revealed. The cryo‐EM method provides more structural information that guides us for screening of BoNT/A mutations with higher receptor binding affinity and more effective therapeutic efficacy.

The BACTH‐based approach enabled the screen covering all possible 20 amino acid substitutions at each interest residue and their combination. We found that S1142, M1144, and T1146 might be located at modifiable positions responsible for the binding capacity of BoNT/A to SV2C. In contrast, T1145, Y1149, R1156, and R1294 are more likely located at fixed positions, as mutations introduced into those four residues will not increase but rather decrease BoNT/A binding activity. Our results are in agreement with a previous study, which found that the T1145A/T1146A mutation in Hc/A completely abolished and R1156E/R1294A mutation significantly reduced the binding to SV2C‐L4 [27]. Other G1292R/W1266A mutation also resulted much lower toxin's entry activity indeed due to the two amino acids locate at relative fixed position [43]. Our results identified that the mutations S1142I/V/Q/F and M1144L/V/I enhanced the binding to SV2C‐L4 (Figure 3D), with T1146S in addition, which may be associated with reduced steric hindrance to serine.

The amino acid residues of BoNT/A isoforms at 1142 and 1144 loci are polymorphic. For the 1142 site, it may be S in botulinum neurotoxin subtype A1, A2, A3, A7, A8, N in subtype A4, or T in subtype A6. Whereas for the 1144 site, it may be M in subtype A1, A3, A4, V in subtype A2, A5, A8, L in subtype A6, or T in subtype A7. The residue variations may alter the binding affinity to SV2C [44]. Previous work demonstrated that the substitutions of D at site 1141, N at site 1142, and R at site 1292 explain 1000‐fold lower potency of BoNT/A4 than BoNT/A1 [17]. Those three amino acid residues are distributed along the SV2 binding interface. The natural substitutions found in BoNT/A isoforms experienced evolution for a long history, also clustered along toxin‐receptor interaction interface, which contributes to the differences in the potency of toxin subtypes. It also suggested combinational mutation strategy on the residues suchlike can be subject to engineer.

The binding probability analyses by ScanNet show that each amino acid residue in VLTS nearly ranked the highest binding affinity to SV2C than those in BoNT/A wt and FLTT (Figure 3G). It may be explained by the synergistic effect of the interactions among the amino acid side chain groups of those modifiable positions in toxin with the relative counterpart groups in SV2C receptor. Though VLTS showed a slightly higher KD value than that of FLTT in the SPR assay, its capabilities for the cell membrane binding and SNAP25 cleavage, slightly superior if not the same, are comparable to those obtained in cultured cortical neurons (Figure 5B,C and Figure S5). These results may reflect the complexity of physiological conditions in which could have other factors that affect the cellular binding. Anyhow, the two engineered BoNT/A always exhibited higher activity of the SNAP25 cleavage and sIPSC activity blockage than BoNT/A wt (Figure 5D,E). Previous work has shown that the efficient cellular entry of BoNT/A is not only attributed to high binding affinity to the protein receptor SV2 but also the co‐receptor gangliosides, even tripartite of gangliosides‐synaptotagmin1‐SV2 nanocluster on the surfaces of neurons [45]. Besides, SV2 is not the only BoNT/A1 receptor, and FGFR3 in motor neurons can also be bound by BoNT/A [29]. Anyway, our preclinical studies in mice confirmed that engineered BoNT/A VLTS and FLTT showed higher functional efficacy than BoNT/A wt indeed (Figure 6A–C; Figure S9 and Table 1). Although the binding of BoNT/A to SV2 is relatively strong compared to BoNT/B with its receptor, and even that the binding is further promoted by the glycosylation of SV2 and the co‐receptor of PSG, improvement still could be made to make the overall cellular binding more efficient by protein engineering.

Given that commercially available BoNT/A has already balanced benefits and risks, if it could be modified to further optimize the risk‐benefit ratio, it would not only be more favorable to patients but also expand its potential applications across diverse medical fields. In the present work, compared to BoNT/A wt, engineered BoNT/A VLTS with improved receptor binding capacity exhibited higher functional efficacy, lower toxicity, and lower diffusivity in DAS, sweat test, MLB, and PET/CT imaging assays. Our findings from the atomic, molecular, and cellular levels to the animal level supported that enhanced Hc/A binding to the SV2 receptor contributes to the overall activity and therapeutic benefit of BoNT/A. The main limitation of BoNT/A in clinical use is the unwanted side effects and high occurrence of neutralizing antibodies in patients due to toxin diffusion from the injection site, thus reducing or even eliminating the effectiveness of toxin therapy [12, 13, 41]. Preclinical local toxin intramuscular application imaged by PET/CT, clinical relevance DAS efficacy test, and the starch‐iodine sweat test showed engineered BoNT/A VLTS significantly restricted to the injection site (Figure 6A‐C, E‐F). Finally, its tolerability index can be increased to much greater extent, nearly four‐fold compared to BoNT/A wt (Table 1). Future clinical studies will expect lower doses need in aesthetic and medical applications, with significantly better safety profiles.

## 4 Materials and Methods

### 4.1 Materials and Reagents

The SNAP25 antibody (GTX113839), β‐actin antibody (YM3028), SV2A antibody (HPA007863), and SV2C antibody (ab324234) were purchased from GeneTex, Immunoway, MilliporeSigma, and Abcam, respectively. Neurobasal Medium, B‐27 Supplement, and GlutaMAX were purchased from Thermo Fisher Scientific.

### 4.2 Cryo‐EM Data Collection

The full‐length BoNT/A wt was stored in 20 mmol sodium phosphate buffer containing 150 mmol NaCl, pH7.4, and the cryo‐EM study was conducted under identical buffer conditions using the sample at a concentration about 500 µg/mL on glow‐discharged Quantifoil Au R1.2/1.3 200 mesh grids. The grid was blotted and then plunge‐frozen in liquid ethane using Vitrobot Mark IV (Thermo Fisher Scientific). The temperature and humidity of Vitrobot chamber were set at 4 °C and 100%, respectively. The prepared cryo‐EM grids were submitted to data collection using a Titan Krios G3 cryo‐TEM (Thermo Fisher Scientific) operated at 300 kV and equipped with a Bioquantum K3 direct electron detector (Gatan). A total of 33,051 movies of the BoNT/A were captured using EPU software (Thermo Fisher Scientific) with a total electron dose of ∼60 e‐/Å^2^, 40 frames, and an exposure time of 3 s. The detailed parameters for data collection are shown in Table S1.

### 4.3 Image Processing and 3D Reconstruction

All cryo‐EM data were processed using cryoSPARC v4 [46]. The Patch Motion Correction was used to correct the beam‐induced movement, and Patch CTF was used to estimate contrast transfer function parameters for each movie. We manually picked ∼500 particles to create 2D templates, then used the template to automatically pick 1.4 M particles with a box size of 384 × 384 pixels. After several rounds of reference‐free 2D classification, the ab initio reconstruction, heterogeneous refinement, and non‐uniform refinement were performed in sequence [47]. The final reconstructions of open state of BoNT/A were obtained with 336 k particles at 2.85 Å resolution, semi‐closed state of BoNT/A was obtained with 230 k particles at 2.80 Å resolution, which was then sharpened using EMReady (Figure S1) [48].

### 4.4 Model Building and Refinement

CryoNet predicted structure of BoNT/A was used as the initial model for model building the open‐state structure, and the final open‐state structure was used as the initial model for the semi‐closed state [49]. Each domain of the initial model was rigid‐body docked into the cryo‐EM map using Chimera [50], and the whole model was then submitted to real‐space refinement using PHENIX [51]. The refined model was further manually checked and adjusted using Coot [52]. The quality of the models was assessed using MolProbity. Statistical details are shown in Table S1. Energy minimization of all complex models was further performed using Rosetta [53].

### 4.5 BACTH Assay

The BACTH technique was initially proposed by Daniel Ladant lab [54]. Here, the BACTH assay was conducted according to the instruction provided by the manufacturer (Euromedex, France). Two plasmids of pUT18C and pKT25 were used for the screen assay. The unique extracellular luminal domain L4 of SV2C fragment containing the toxin‐binding site amino acid residues 454–579 was inserted into pKT25 to obtain T25‐SV2C‐L4. The Hc/A was inserted into pUT18 to construct T18‐Hc/A. Hc/A mutant libraries were produced using primers containing random nucleotide triplets (NNK) at the indicated positions. Each Hc/A mutant library was co‐transformed with the T25‐SV2C‐L4 plasmid into the *E. coli* reporter strain BTH101 by chemical transformation, then spread and screened on 25 cm LB plates containing 100 µg/mL ampicillin, 50 µg/mL kanamycin, 0.5 mmol IPTG, and 40 µg/mL X‐Gal. After all the experimental plates were placed in an incubator at 30°C for 36 h, plasmids were extracted from conspicuous blue colonies by SanPrep Column Plasmid Extraction Kit (Sangon Biotech, Shanghai, China) for Sanger sequencing the interest gene, especially the mutation sites. The probability of covering all 20 amino acids at the selected mutation site was calculated using the Clark‐Carbon equation p = 1‐(1‐f) N, where f = 1/20, the possible frequency of 20 different amino acids. N is the total number of colonies on the experimental plates. In our experiment, the probability of covering all 20 amino acids at each mutation site is more significant than 99.9%, with almost about 400 colonies in total.

### 4.6 β‐Galactosidase Activity Assay

The assay was carried out as Griffith and Wolf reported previously [38, 55]. The bacteria were seeded in a 96‐deep well culture plate, and 0.6 mL of LB medium containing 100 µg/mL ampicillin, 50 µg/mL kanamycin, and 0.5 mmol IPTG was added in each well in advance. The plate was sealed with a sterile film and cultured in an incubator shaken at 250 rpm and 30°C for overnight. 50 µL culture was then transferred one by one into a 96‐well plate containing 150 µL LB in each well for detecting OD value at 600 nm. 200 µL culture was transferred in parallel into a 2 mL Eppendorf tube containing 800 µL of Z buffer (8 g Na_2_HPO_4_·12H_2_O, 3.125 g NaH_2_PO_4_·H_2_O, 0.375 g KCl, 0.123 g MgSO_4_·7H_2_O, and 1.35 mL β‐mercaptoethanol dissolved in 500 mL distilled water, pH 7.0), 50 µL 0.01% SDS and 100 µL chloroform were sequentially added, then vortexed for 10 sec. Stood for 2 min, 50 µL up‐layer aliquots were then transferred into a 96‐well plate containing 150 µL of Z‐buffer, 40 µL 0.4% ONPG, and mixed gently, incubated at 28°C for 15–20 min. After that, OD values were measured at 420 nm every 2 min on a microplate reader. The relative β‐galactosidase activity was calculated by an equation: ((OD_420 nm_ at time t2 – OD_420 nm_ at time t1)/t2‐t1 (min))/OD_600 nm_, where t2 and t1 time points were chosen from the linear part of the kinetic curve.

### 4.7 Protein Preparation

The BoNT/A wt gene was fully synthesized according to the amino acid sequence on the NCBI Reference Sequence (accession number: WP_011948511.1) after optimization for codon bias of *E. coli*. It was inserted into pET28a+ in frame of His6‐tag at 5’ terminal by NdeI and EcoRI. Another His6‐tag was fused to the 3’ terminal for easier purification by Ni^2+^ affinity chromatography at a high imidazole of 100 mmol in binding buffer. FLTT and VLTS gene were cloned by site‐directed mutagenesis technology on pET28a+‐ BoNT/A wt. All the obtained proteins were dialyzed against 20 mmol sodium phosphate buffer containing 150 mmol NaCl, pH7.4. The resultant protein was concentrated with a 50 kDa cut‐off Ultrafiltration Centrifugal Tube (Millipore) and subjected to SEC using an Ezload 10/60 chromdex 200PG equilibrated in 25 mmol Tris‐HCl pH 8.0 supplemented with 150 mmol NaCl. The fractions of full‐length BoNT/A wt or mutants were collected, and used for animal experiments and cell experiments. The desired protein purity was assessed using 4–20% SDS‐PAGE and visualized by standard Coomassie brilliant blue staining. Finally, the proteins were quickly frozen in liquid nitrogen and stored at ‐80°C before use. The concentrations of neurotoxins were measured using BCA protein assay kit (Solarbio Lifesciences, Beijing, China, PC0020).

The Hc/A gene was amplified from pET28a+‐ BoNT/A wt using the primers Hc/A‐F: GGGAATTCCATATGATTATCAACACCAGC and Hc/A‐R: CCGGAATTCTTACAGCGGGCGTTCGCC. It was inserted into the NdeI/EcoRI sites to obtain pET28a+‐His6‐Hc/A wt. Subsequently, all the pET28a+‐His6‐Hc/A mutants were constructed by site‐directed mutagenesis. Hc/A and the mutants were routinely expressed in *E. coli* BL21 (DE3) by IPTG induction and purified by Ni^2+^ affinity chromatography.

The SV2‐L4 genes were fully synthesized based on amino acid sequences from the NCBI Reference Sequences, following codon bias optimization for *E. coli*. The synthesis used the following accession number: NM_001278719, range (1396–1794) for SV2A‐L4, NG051558 (range:1225–1605) for SV2B‐L4, and BC100824.1 (range:1495‐1873) for SV2C‐L4. The target genes were cloned into pGEX4T‐1, routinely expressed in *E. coli* DH5α by IPTG induction, and purified by glutathione affinity chromatography. The protein concentrations of Hc/A and SV2‐L4 were all measured also using BCA protein assay kit (Solarbio Lifesciences, Beijing, China, PC0020).

### 4.8 Thermal Stability Analysis of BoNT/A by Differential Scanning Fluorimetry

The thermal stability of recombinant proteins was assessed by nano‐differential scanning fluorimetry (nanoDSF) using a Prometheus NT.48 instrument (NanoTemper Technologies). Purified protein samples were diluted to a final concentration of 0.2–0.5 mg/mL in the corresponding buffer to a total volume of approximately 20–50 µL. Each sample was loaded into high‐sensitivity capillaries (NanoTemper Technologies), and a temperature ramp was applied from 20°C to 95°C at a heating rate of 1.0°C/min. The ratio of fluorescence intensities at 350 nm and 330 nm (F350/F330) was plotted as a function of temperature. The melting temperature (Tm) was defined as the inflection point of the unfolding transition, corresponding to the minimum of the first derivative of the ratio curve (d(F350/F330)/dT). Data from nanoDSF were exported from PR. ThermControl and PR.

### 4.9 Surface Plasmon Resonance (SPR) Assay

The binding affinity between Hc/A mutants and SV2C‐L4 was measured by SPR assay on a Biacore system (T200, GE). Briefly, 5 ng/mL GST‐SV2C‐L4 was immobilized on a CM5 chip and activated with 0.4 m EDC and 0.1 m NHS (1:1). The chip was exposed to a series of different concentrations of Hc/A, followed by regeneration with glycine‐HCl buffer, pH 2.0 to remove Hc/A from GST‐SV2C‐L4. According to the manufacturer's instruction, the binding affinity constant (KD) was calculated using the Biacore system software.

### 4.10 Pull Down Assay

GST‐SV2C‐L4 (300 µg) was incubated with 60 µg Hc/A immobilized on Ni^2+^‐ NTA affinity chromatography beads at 4°C for 1 h, rat brain lysate or rat hippocampal neuron cell lysates were incubated with 5 µg Hc/A immobilized on Ni^2+^‐ NTA affinity chromatography beads at 4°C for 1 h. Washed with binding buffer containing 20 mmol PB, pH7.4, 100 mmol NaCl, 20 mmol imidazole, and 0.5% TritonX‐100 for three times, then eluted with elution buffer containing 20 mmol PB, 100 mmol NaCl, 500 mmol imidazole, and 1% TritonX‐100 at pH7.4. Samples were routinely analyzed by SDS‐PAGE.

### 4.11 Neuron Culture, Toxin Cleavage Activity Assay by Western Blot, and Toxin Binding Assay by Confocal Laser Scanning Microscope

Cultured cortical neuron were prepared from SD rat foetuses at 18 ± 1 days of pregnancy and cultured in a Neurobasal medium supplemented with B27 and Glutamax for 12–18 days as previously described [33]. The full‐length BoNT/A (BoNT/A wt) and the mutant toxins were diluted with Neurobasal medium before the experiment, and added to the primary neuron cultures, incubated at 5% CO_2_, 37°C for 48 h. The neurons were lysed by RIPA, with 1 mmol PMSF, then the cell lysate samples were subjected to SDS‐PAGE. Proteins were transferred onto a nitrocellulose membrane (Millipore). The membrane was blocked with 5% skimmed milk and incubated with primary anti‐SNAP25 polyclonal antibodies to detect the intact and cleaved SNAP25 at 4°C overnight. After washed three times with PBS‐T buffer, the membrane was incubated with anti‐rabbit HRP‐secondary antibodies at room temperature for 2 h, washed another three times with PBS‐T buffer, added ECL substrate. Bands were visualized on a Tanon gel imaging system (4600SF, Tanon, Shanghai, China). Cultured cortical neurons with no toxin treated were used as a negative control.

For the toxin binding assay, Neuro‐2a cells (3 × 10^5^ cells/well) and rat hippocampal neurons (1 × 10^5^ cells/well) were seeded in 12‐well plates. The cells were then incubated with 100 nmol EGFP‐Hc/A for 2 h (Neuro‐2a cells) or 15 min (hippocampal neurons) at 37°C. The toxin binding was observed, and images were acquired by confocal microscopy in stellaris mode (Leica). All experiments were repeated three times independently.

### 4.12 sIPSC Recordings of Toxin Treated Cortical Neurons by Whole‐Cell Patch‐Clamp

Cultured cortical neurons (DIV 12–21) were treated with 31.25 pmol toxins for 48 h. Whole‐cell recordings were performed using a pipette with a resistance of 4–7 MΩ. The pipette was filled with the following solution: 153 mmol CsCl, 1 mmol MgCl_2_, 10 mmol HEPES, 4 mmol Mg ATP (pH 7.2, adjusted with CsOH). Synaptic current was monitored using EPC‐10/2 amplifier (HEKA) at a holding potential of ‐70 mV. The bath solution contains: 125 mmol NaCl, 4.5 mmol KCl, 5 mmol CaCl_2_, 1 mmol MgCl_2_, 10 mmol HEPES, 10 mmol glucose, 20 mmol TEA Cl (pH 7.3, adjusted with NaOH). The data was analyzed using Mini Analysis software (Synaptosoft) and Igor (Wavemetrics).

### 4.13 Mouse Lethality Bioassay

BoNT/A wt and its mutants were serially diluted by two‐fold with PBS containing 0.25% BSA. The mice were injected intraperitoneally or intramuscularly into gastrocnemius muscle with the indicated toxin at an appropriate dose. For IP injection, mice were administrated toxin doses of 9.375, 18.75, 37.5, 75, 150 pg in 100 µL per animal. For IM injection, mice were administrated toxin doses of 18.75, 37.5, 75, 150, 300 pg in 10 µL per animal. Mice in the control group received equal treatment with PBS containing 0.25% BSA alone. Animal survival was recorded every 12 h for 4 days. The LD_50_ was calculated using probit analysis by fitting a weighted linear regression of mortality against log dose. The LD_50_ was derived as the antilog of the dose at a probit of 5.0.

### 4.14 Digit Abduction Score (DAS) Assay

DAS assay is based on the typical startle response of mice when their tails are simply caught and suspended upside down [34, 56], the mice show a hind limb extension and toe abduction. The mice were injected intramuscularly into the gastrocnemius muscle of the left hind limb with 10 µl of toxin at a dose of 1, 5, 15, 30, 60 pg/animal. BoNT/A wt and its mutants were serially diluted with PBS containing 0.25% BSA, and the control group mice were only treated with the vehicle. 31‐gauge needle attached to a 50 µl syringe was used, and by fixing the needle to expose a fixed 3 mm length using a truncated pipette tip to control the injection depth. DAS tests were performed every 2 h for the first 12 h after injection, and then every 12 h afterward. Two observers blind to the treatment, carry out scoring independently. At the same time, the mice were weighted for observation of the changes of their body weight. For dose‐response data analysis, each dose (pg/mouse) was converted to its natural logarithm as the *x*‐axis, and the highest average DAS score per dose group was plotted as the *y*‐axis. The data were fitted to a four‐parameter logistic equation, with the lower asymptote constrained to 0 and the upper asymptote constrained to 4. The dose value corresponding to half‐maximal DAS (DAS 2) obtained from the equation was then converted to the ED_50_ by inverting its natural logarithm back to the linear scale.

### 4.15 Body Weight Assay

Mice were injected intraperitoneally with 100 µL of toxin at a dose of 0.25, 0.5, 1, 5, 15, 30, 60 pg/animal. Animal body weight was recorded daily over the 5‐day experimental period, and the percentage change from day 0 (%BW) was calculated for each dose. A linear regression model was applied to estimate the theoretical dose at which no weight loss (0% change from day 0) would occur. The tolerability index for BoNT/A wt and mutants was then determined as the ratio of this theoretical no‐weight‐loss dose to the ED_50_ (0%BW/ED_50_).

### 4.16 Starch‐Iodine Sweat Test

The starch‐iodine sweat test was used to detect perspiration [57]. Kunming female mice (20 g) received BoNT/A wt or its mutants exclusively on the right paws to evaluate the local therapeutic efficacy of the toxin, while the left paws were left untreated to detect the systemic diffusion of the toxin. 5 U of toxins in 16 µL were subcutaneously injected with a 31‐gauge needle attached to a 20 µL microsyringe for 6 h, with PBS as a negative control. After mice were anesthetized, pilocarpine hydrochloride at a dose of 5 mg/kg body weight was also subcutaneously injected to induce hyperhidrosis. The two footpads of the animal were spread with 50% (w/v) starch suspension in castor oil and then painted with 2% (w/v) iodine solution in ethanol. When starch dissolves in sweat, it turns black upon contact with iodine. The black spots are identified as active sweat gland sites. The mice's paws were imaged and the black spots were counted using ImageJ software.

### 4.17 Synthesis of ^68^Ga‐NOTA‐HC/A

For ^68^Ga‐labeling, the Hc/A wt was first conjugated with a bifunctional chelator 2‐S‐(4‐Isothiocyanatobenzyl)‐1,4,7‐triazacyclononane‐1,4,7‐triacetic acid (p‐SCN‐Bn‐NOTA; Macrocyclics), adjusted pH to 8.7 with 0.1 mol Na_2_CO_3_ buffer, and kept chelating at RT for 2 h. Then, 0.1 mol ammonium acetate was used as dialysate to remove the free chelating agent. Finally, 200 µl of the peak fraction of the ^68^Ge/^68^Ga generator eluate (280 MBq) was added to NOTA‐ Hc/A wt, adjusted pH to 5 with 0.1 M NaOH, and incubated at RT for 15 min to obtain ^68^Ga‐ NOTA‐ Hc/A wt. The synthesis of ^68^Ga‐NOTA‐VLTS was the same as above.

### 4.18 PET/CT Nuclear Imaging

Imaging was performed on a small animal PET/CT system (Madic Technology Co., Ltd., China). Mice were received either intravenous injection of 100 µg NOTA‐Hc/A wt or VLTS labeled with 200 µCi of radioactivity, or locally administration of 2.5 µg of NOTA‐Hc/A wt or VLTS labeled with 6.5 µCi of radioactivity into the left gastrocnemius muscle. For tail intravenous administration, a 29‐gauge insulin syringe (0.33 mm × 13 mm, BD Ultra‐Fine) was used, whereas a 31‐gauge needle attached to a 10 µl microsyringe was employed for gastrocnemius muscle administration. After complete drug delivery, the needle was retained for 5 s before withdrawal to prevent backflow, with the injection performed slowly. Animals were anesthetized using 2%–3% isoflurane in oxygen and placed prone in the scanner gantry for PET/CT imaging. Each group of mice was imaged at 2 h after injection.

All imaging examinations were performed using PET/CT (MadicLab PSA094, Shandong Medyinghua Technology Co., Ltd., China), and PET/CT images were collected 120 min after injection. The entire PET/CT scan was performed for 30 min, and the PET 3D image reconstruction algorithm based on OSEM was used for reconstruction processing. CT scans were performed using parameters of 80 kV and 70 mAs for attenuation correction and image fusion localization, with a final resolution of 0.63 mm *×* 0.63 mm *×* 0.63 mm. All data were obtained and analyzed by using PMOD software (version 4.4). PMOD Technology Co., Ltd.

### 4.19 Statistical Analysis

Each experiment was repeated three times (*n*≥3), and all data were expressed as mean ± SD or mean ± SEM as indicated. Statistical analyses were performed using one‐way ANOVA followed by post hoc Dunnett's test for multiple comparisons. *P*‐values below 0.05 were considered to be statistically significant. GraphPad Prism 9.5 software was used to carry out statistical analyses.

## Author Contributions

Dongsheng Wang, Dejuan Zhi, and Dongsheng Lei conceived the work, designed experiments, analyzed the data, and revised the manuscript. Wenrui Wang, Linjin You, Fenfen Gao, and Rong Nie contributed to identify the key residues for improving toxin‐SV2C binding affinity and detect the biological activities of toxins in vitro. Zhaxi Zerang, Shanquan Wu, and Xinyao Liu contributed to BoNT/A cryo‐EM structure. Linjin You, Ziye Liu, Wenrui Wang, Xiaoru Wang, Jinghan He, Xiaoru Wang, and Fuwei Qi prepared toxin proteins and completed the comparison of the potency of the toxins and the preclinical animal studies. Wenrui Wang, Chengmu Zhao, Wantong Ma, and Bo Liu drafted the manuscript. All authors have given approval to the final version of the manuscript.

## Conflicts of Interest

Mutations in BoNT/A reported in this manuscript have been included in patent applications filed by Lanzhou University, with D.W., D.Z., W.W., and Z.L. as inventors. The remaining authors declare no competing financial interests.

## Ethics Statement

All experimental procedures were approved by the Institutional Animal Care and Use Committee of Lanzhou University. The kunming mice, C57BL/6J mice, and pregnant SD rats were purchased from the Experimental Animal Center of Lanzhou University (approval No: SCXK(GAN)‐2023‐0003)). Adult Kunming mice were used in preclinical experiments except that C57 mice were used in PET/CT imaging. The mice were cage‐housed in an environment that was maintained at temperature from 20°C to 26°C and humidity from 40% to 60% in a 12 h light and dark cycle. All mice were allowed to move around freely and had access to food and water.

Humane endpoints were rigorously defined and enforced. When animal experiments end or during which, animals meet any predefined endpoint criterion, exhibit severe paralysis, demonstrate severely compromised respiration, or losing the ability to access food and water independently, were euthanized promptly via CO_2_ inhalation followed by a secondary physical method of cervical dislocation to ensure death.

## Supporting information




**Supporting File 1**: advs75164‐sup‐0001‐Data.docx.

**Supporting File 2**: advs75164‐sup‐0002‐SuppMat.docx.

## Data Availability

The cryo‐EM map and the atomic model of BoNT/A have been deposited into the Protein Data Bank under the accession codes EMD‐51433 and PDB 9GKQ. The cryo‐EM map and the atomic model of semi‐closed state of BoNT/A have been deposited into the Protein Data Bank under the accession codes EMD‐64966 and PDB 9VCS. Structures used for comparative analysis in this manuscript can be found with the following PDB accession codes: PDB 3BTA (crystal structure of BoNT/A1 holotoxin); PDB 9F3C (SV2B‐BoNT/A1 (pH 5.5); PDB 9F2Y (SV2B‐LD‐BoNT/A1 (pH 5.5); PDB 9F2B (SV2B‐LD–BoNT/A1); PDB 4JRA (crystal structure of the SV2C‐LD Hc/A1 complex).
