# Supplementary material for: Cryo‐EM Structure Guided Engineering of Botulinum Neurotoxin A With Advanced Receptor Binding Affinity and Therapeutical Benefits
Source: Adv Sci (Weinh). 2026 Apr 7;13(33):e16713. doi: 10.1002/advs.202516713 (PMC13271637; doi:10.1002/advs.202516713)

Supplementary information for

**• Cryo-EM structure guided engineering of botulinum neurotoxin A with advanced receptor binding affinity and therapeutical benefits**

Wenrui Wang^1,2^, Zhaxi Zerang^2,3^, Linjin You^1,2^, Ziye Liu^1,2^, Rong Nie^1,2^, Fuwei Qi^1,2^, Fenfen Gao^1,2^, Chengmu Zhao^1,2^, Wantong Ma^1,2^, Jinghan He^1,2^, Xiaoru Wang^1,2^, Shanquan Wu^2,3^, Bo Liu^1,2^, Xinyao Liu^2,3^, Dongsheng Lei^2,3^*, Dejuan Zhi^1,2^*, Dongsheng Wang^1,2^*

Corresponding author: *Dongsheng Wang: dswang@lzu.edu.cn

*Dejuan Zhi: zhidej@lzu.edu.cn

*Dongsheng Lei: leids@lzu.edu.cn

**Uncropped Gel Images.**

Uncropped gel image of Figure 4A


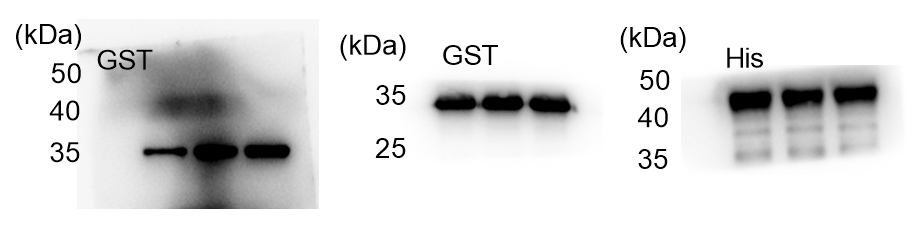


Uncropped gel image of Figure 4B


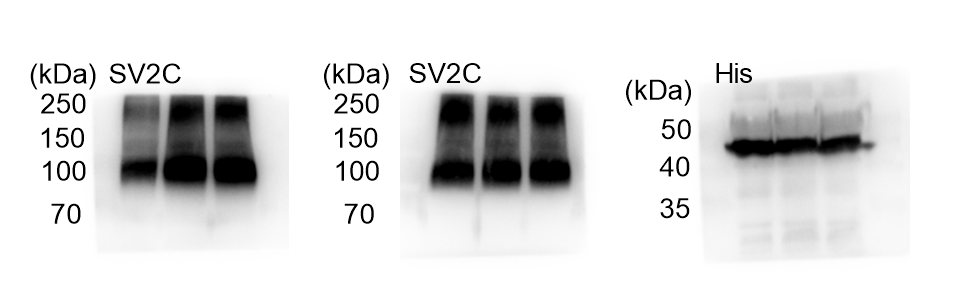


Uncropped gel image of Figure 4E


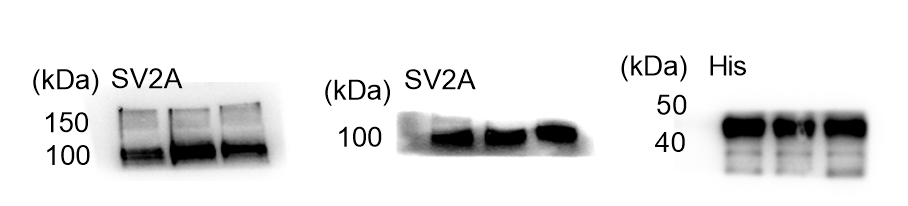


Uncropped gel image of Figure 5A


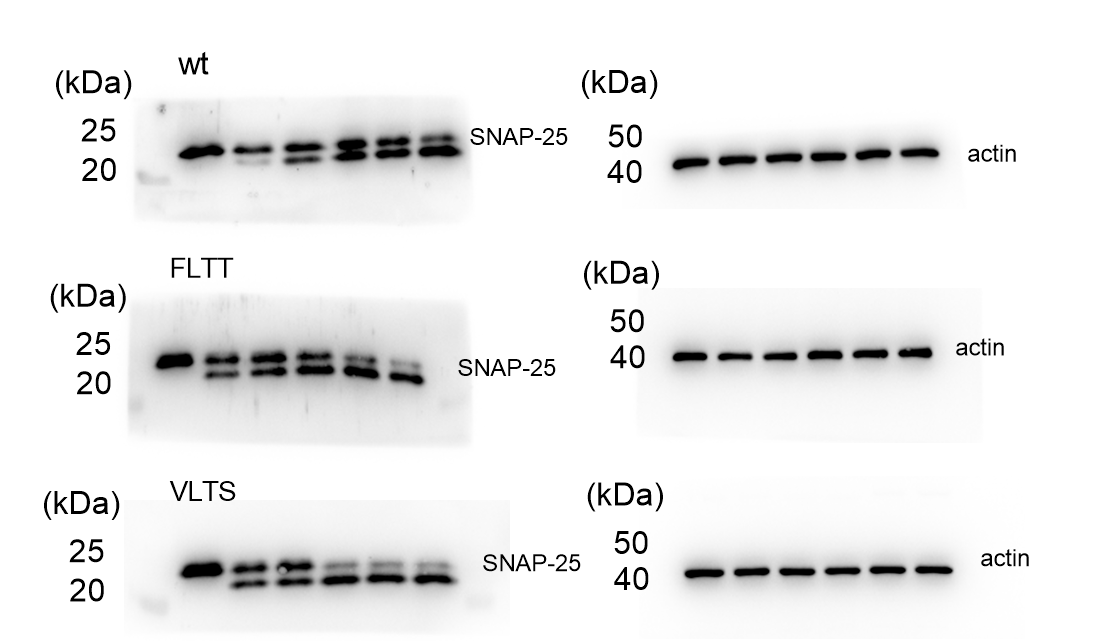


Uncropped gel image of Supplementary Figure 4


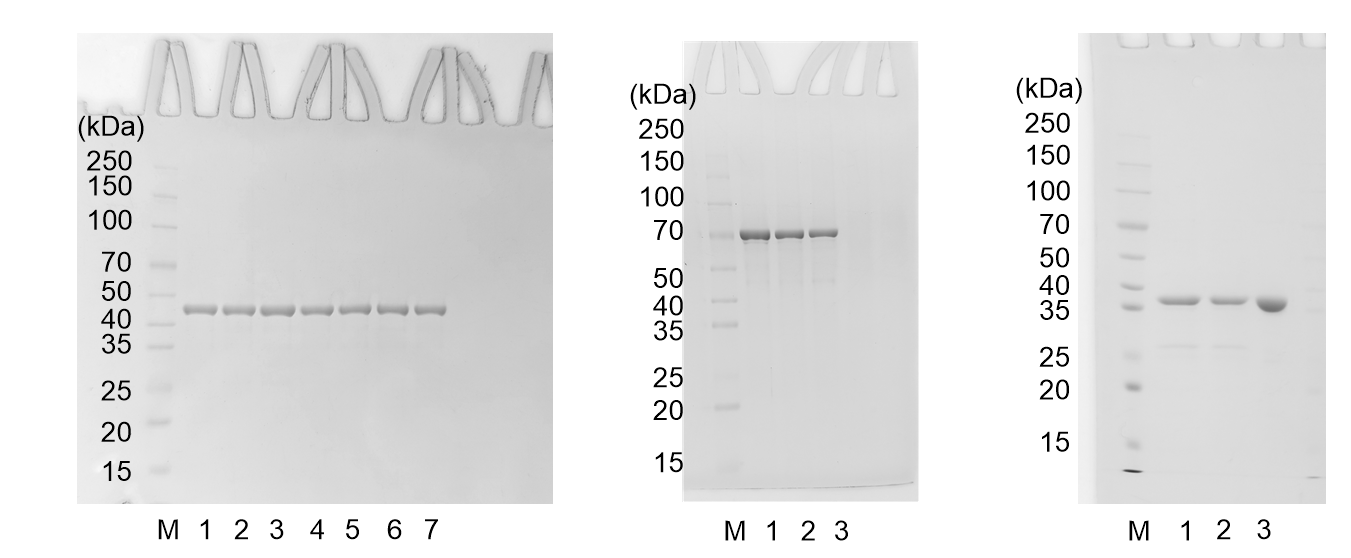


Uncropped gel image of Supplementary Figure 4


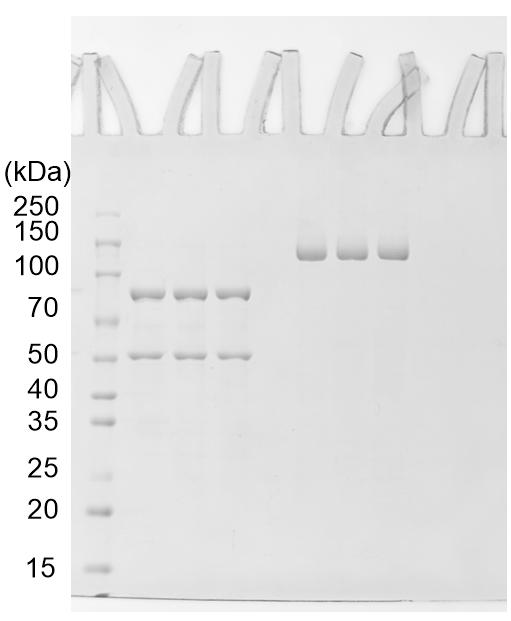


The original image of Figure 4C


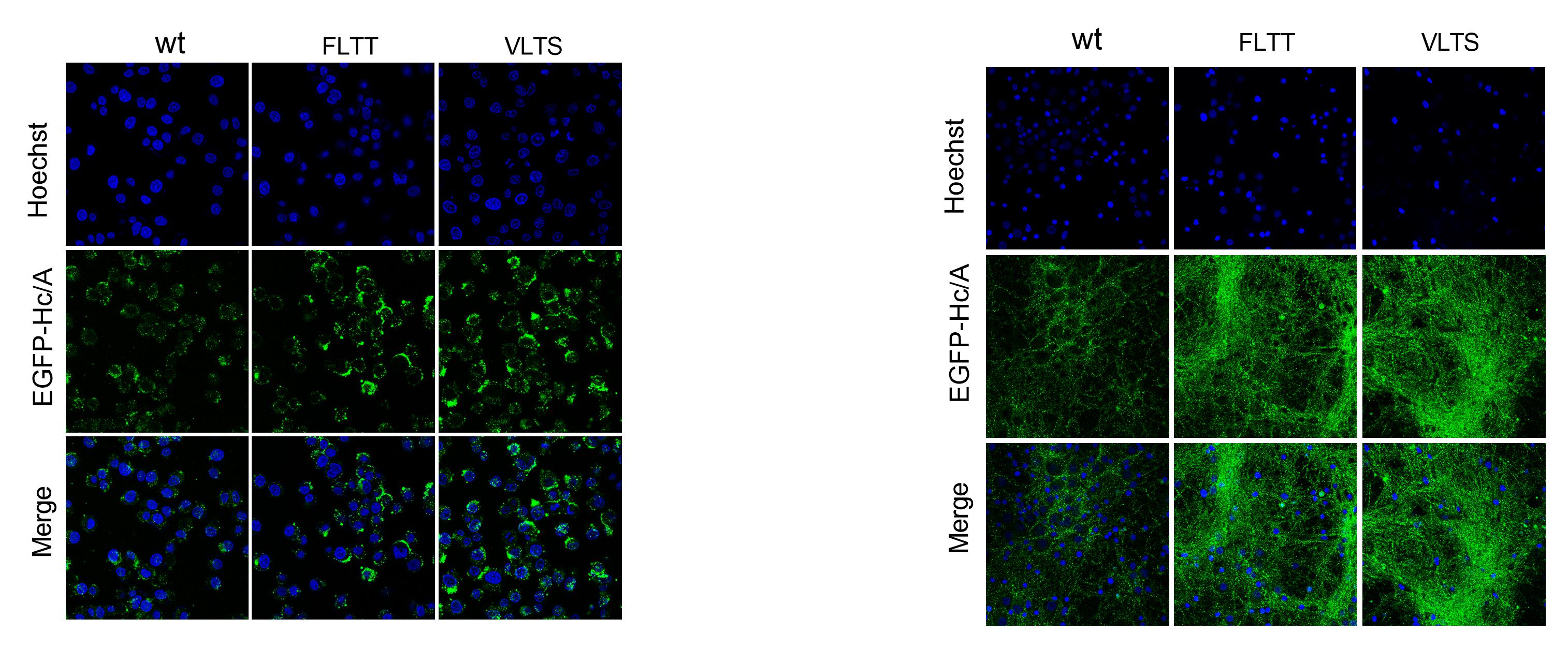


The original image of Figure 4F


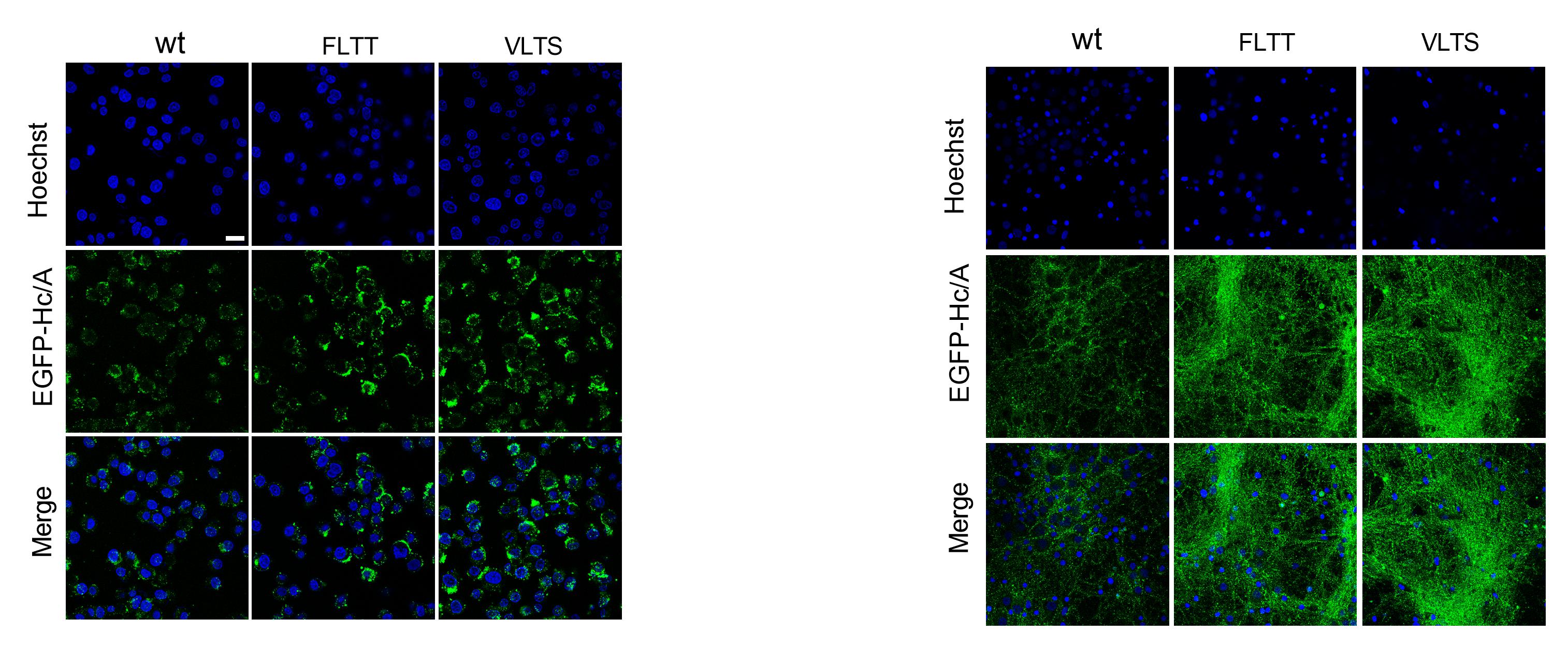


The original image of supplementary Figure 6


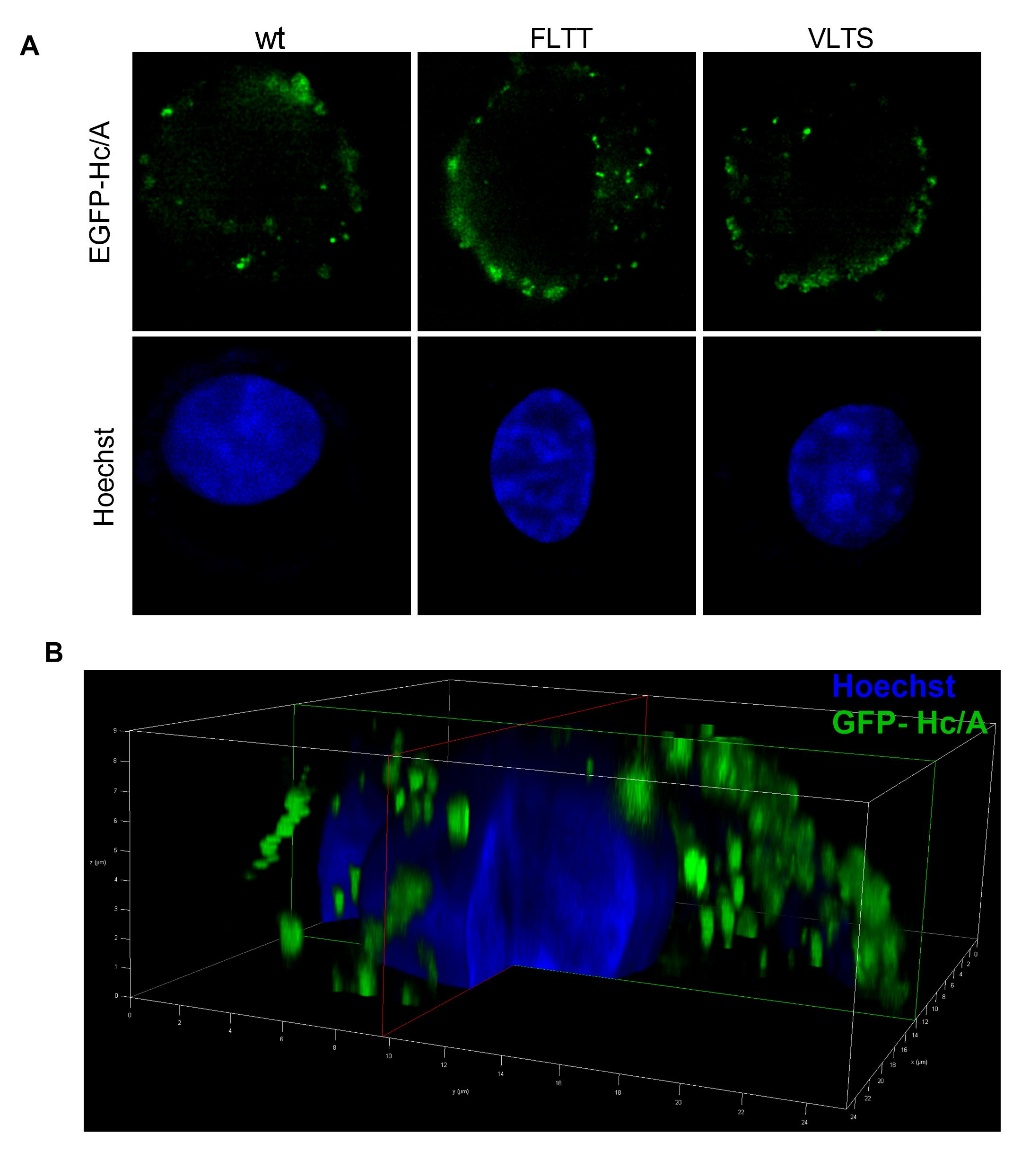

Supplement: Supplementary file 1 — Supporting File 1: advs75164‐sup‐0001‐Data.docx. [file ADVS-13-e16713-s002.docx]
