# Supplementary material for: Cryo‐EM Structure Guided Engineering of Botulinum Neurotoxin A With Advanced Receptor Binding Affinity and Therapeutical Benefits
Source: Adv Sci (Weinh). 2026 Apr 7;13(33):e16713. doi: 10.1002/advs.202516713 (PMC13271637; doi:10.1002/advs.202516713)
Supplement: Supplementary file 2 — Supporting File 2: advs75164‐sup‐0002‐SuppMat.docx. [file ADVS-13-e16713-s001.docx]

Supplementary Materials for

**• Cryo-EM structure guided engineering of botulinum neurotoxin A with advanced receptor binding affinity and therapeutical benefits**

*Wenrui Wang^1,2^, Zhaxi Zerang^2,3^, Linjin You^1,2^, Ziye Liu^1,2^, Rong Nie^1,2^, Fuwei Qi^1,2^, Fenfen Gao^1,2^, Chengmu Zhao^1,2^, Wantong Ma^1,2^, Jinghan He^1,2^, Xiaoru Wang^1,2^, Shanquan Wu^2,3^, Bo Liu^1,2^, Xinyao Liu^2,3^, Dongsheng Lei^2,3^*, Dejuan Zhi^1,2^*, Dongsheng Wang^1,2^**

Corresponding author: *Dongsheng Wang: dswang@lzu.edu.cn

*Dejuan Zhi: zhidej@lzu.edu.cn

*Dongsheng Lei: leids@lzu.edu.cn

**This PDF file includes:**

Figs. S1 to S11

Tables S1 to S4

**
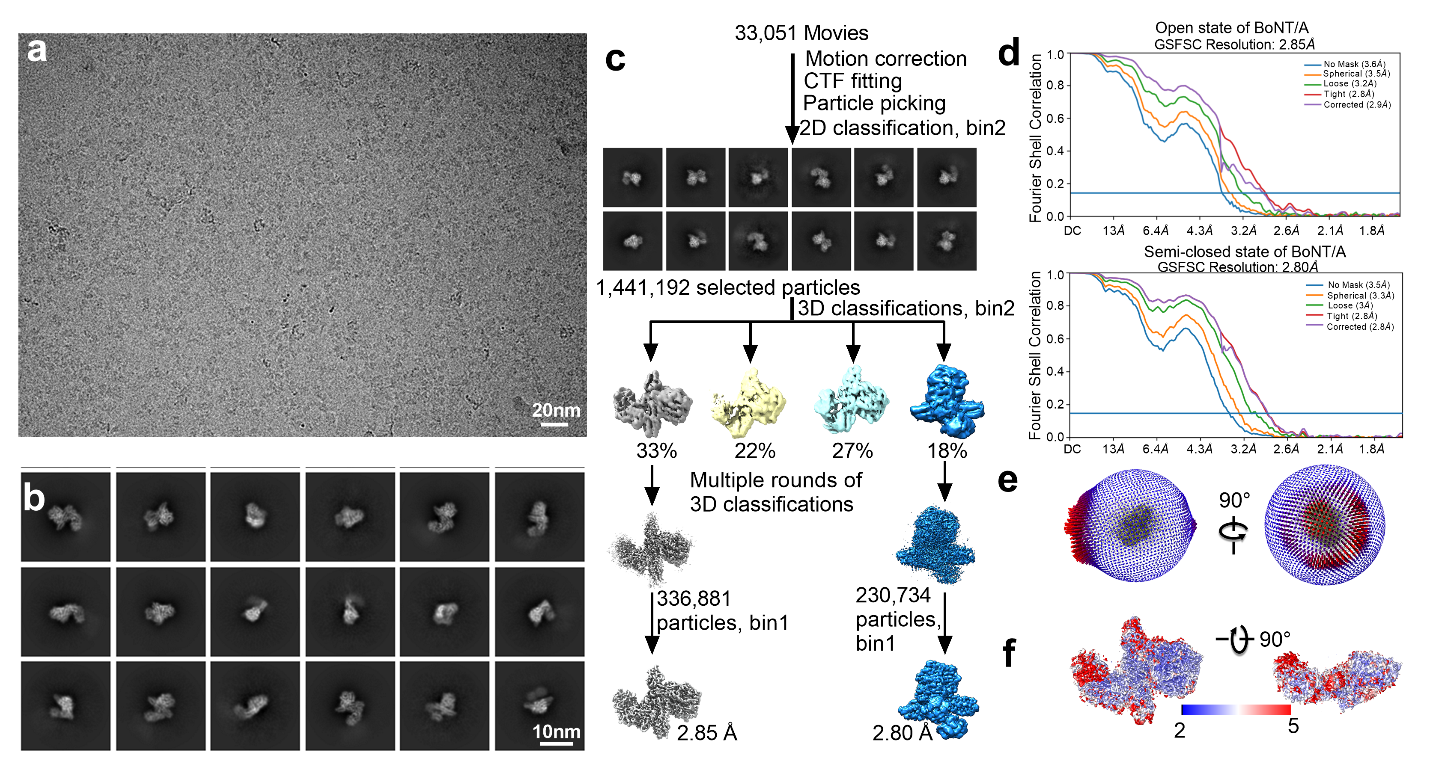
Figure S1. Cryo-EM reconstruction of BoNT/A.** (A) Representative cryo-EM micrograph of BoNT/A. (B) Representative 2D class averages. (C) Image processing pipeline for BoNT/A. (D) Fourier shell correlation curves. (E) Corresponding angular distribution map indicates open state of BoNT/A has a strong preferred orientation. (F) Cryo-EM map of open state colored by local resolution in Å.


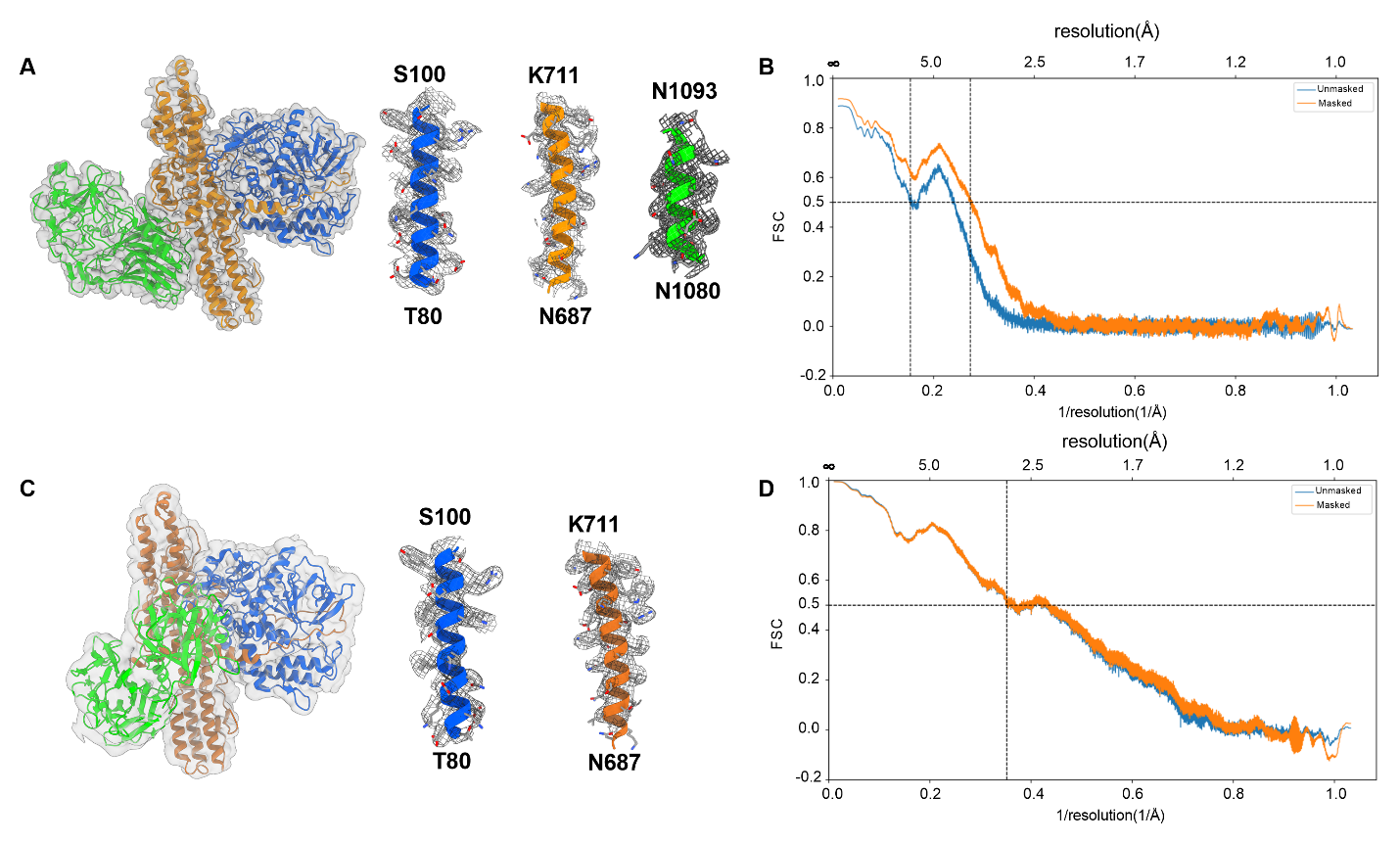


**Figure S2. Cryo-EM structure determination.** (A) Fit of the open-state BoNT/A atomic model into its cryo-EM density map. Detailed views show representative secondary structure elements within each domain. (B) Fourier Shell Correlation (FSC) curve between the open-state BoNT/A structure and its corresponding density map. (C) Fit of the semi-closed-state BoNT/A atomic model into its corresponding cryo-EM density map. Detailed views of secondary structure elements in each domain are also displayed. (D) FSC curve between the semi-close-state BoNT/A structure and its corresponding density map.


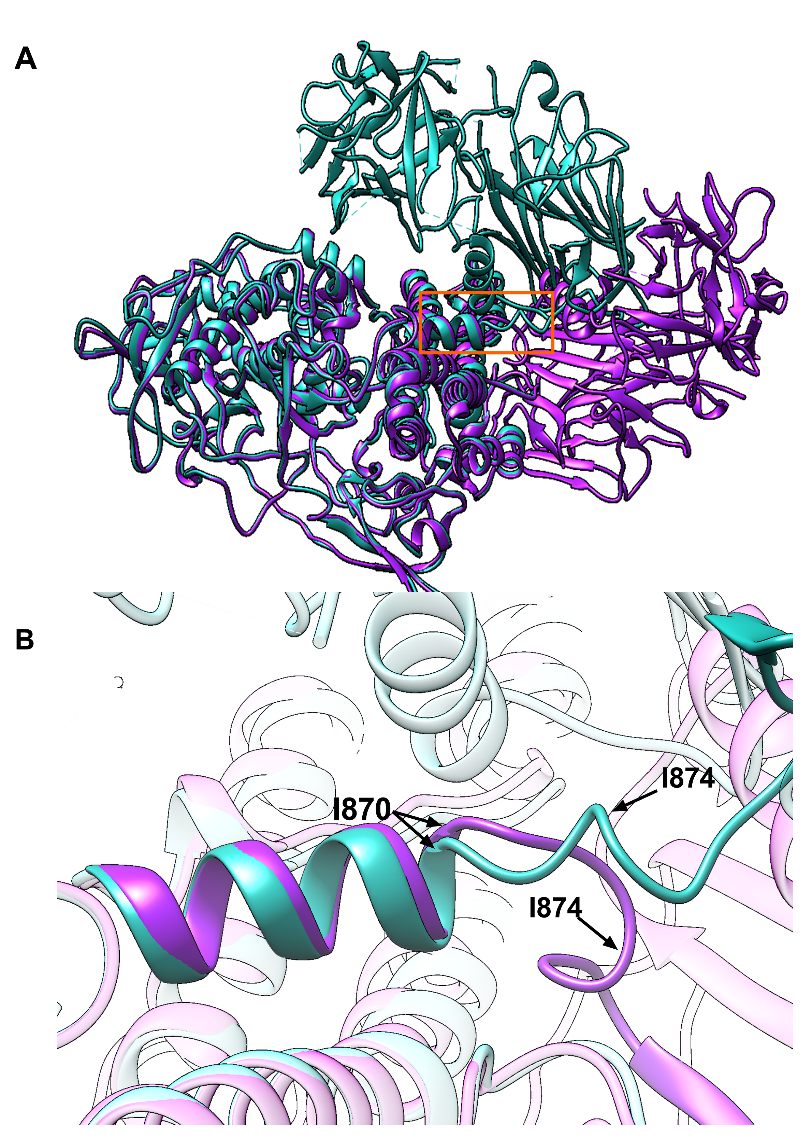


**Figure S3. Comparison between different conformations of BoNT/A.** (A) The open conformation BoNT/A colored in purple, and the semi-closed conformation BoNT/A colored in light sea green. (B) Enlarged projection view of the detailed region highlighted with a dashed box, illustrating that the overall conformational rearrangement initiates at residue I870.
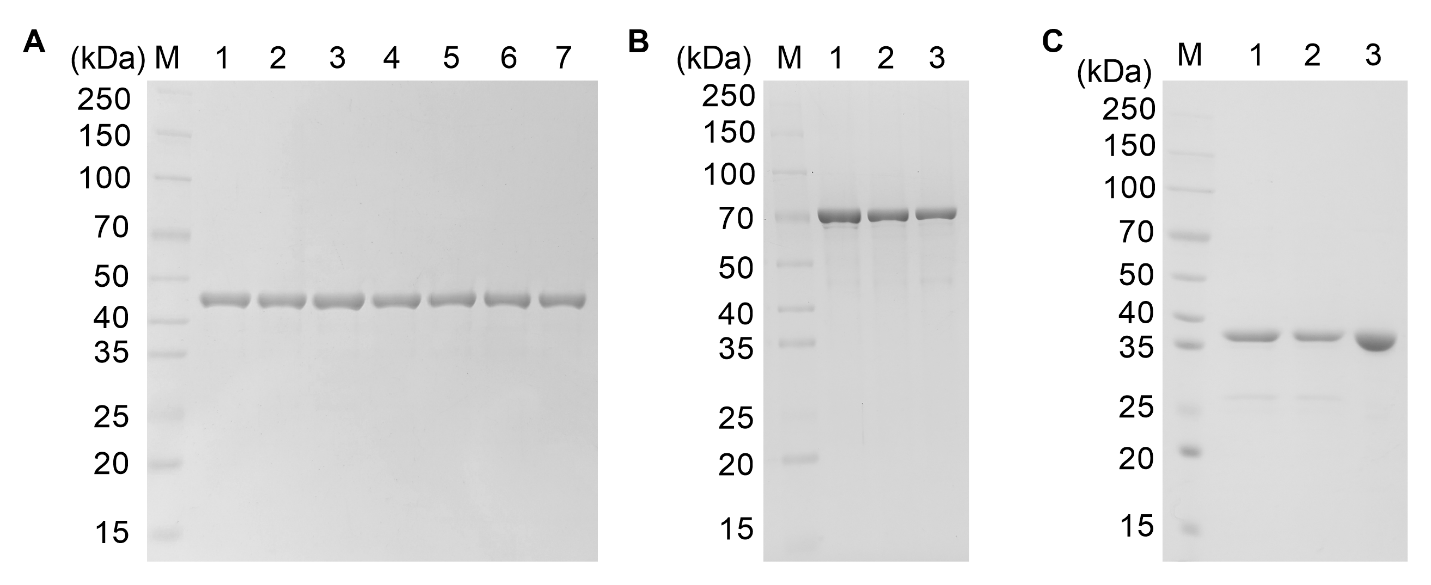


Figure S4. Purification of Hc/A mutants and GST-SV2C-L4. (A) SDS-PAGE gel electrophoresis results of the Hc/A mutant purified proteins. Lanes 1-7 represent the following strains: Hc/A wt, mutants FLTT, FLTS, VLTT, VVTT, VLTS and HWTT. (B) SDS-PAGE gel electrophoresis results of the EGFP- Hc/A mutant purified proteins. Lanes 1-3 represent EGFP- Hc/A wt, EGFP- FLTT and EGFP- VLTS. (C) SDS-PAGE gel electrophoresis results of GST-SV2s-L4. Lanes 1-3 were GST-SV2A-L4, GST-SV2B-L4 and GST-SV2C-L4, respectively.


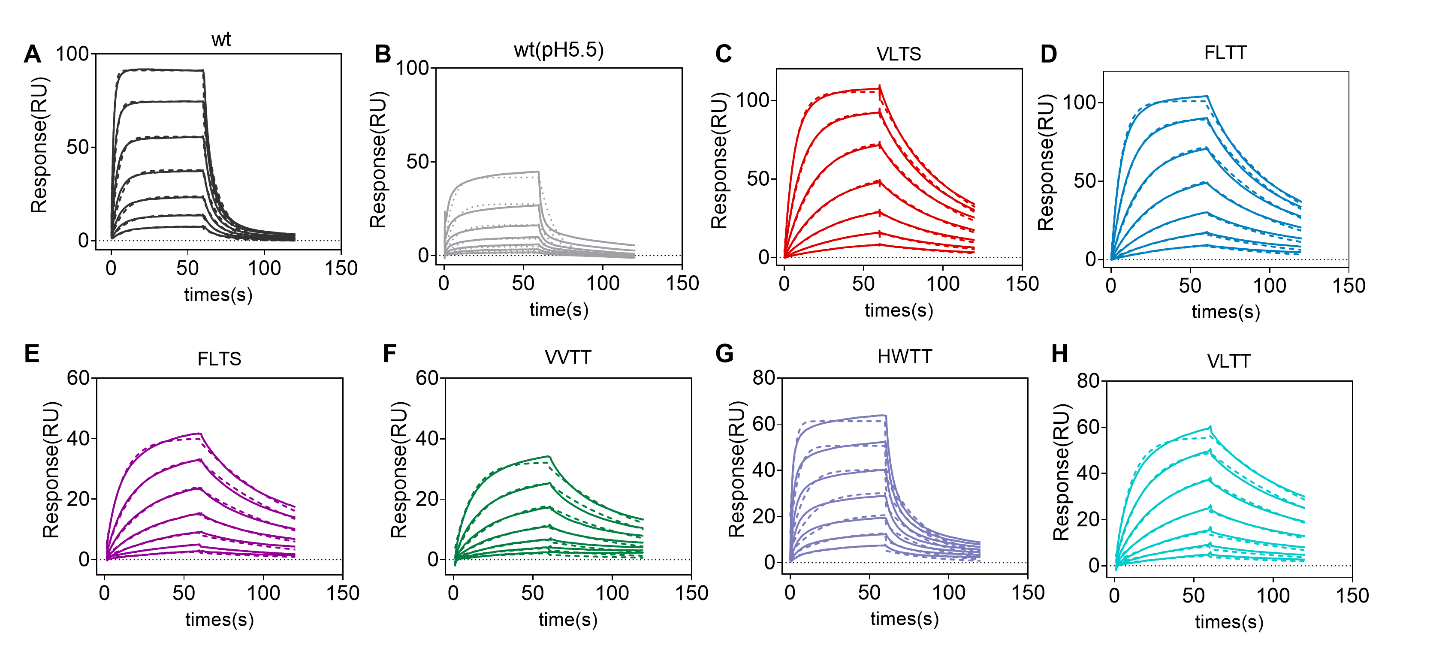


Figure S5. Characterizing Hc/A or BoNT/A binding to SV2C-L4 using the SPR assay. Characterizing Hc/As binding to SV2C-L4 using the SPR assay. Surface plasmon resonance sensorgrams of ligand binding to immobilized SV2C-L4 (solid line) are overlaid with a fit to a 1:1 binding model (dashed line). Representative binding and dissociation curves are shown here.


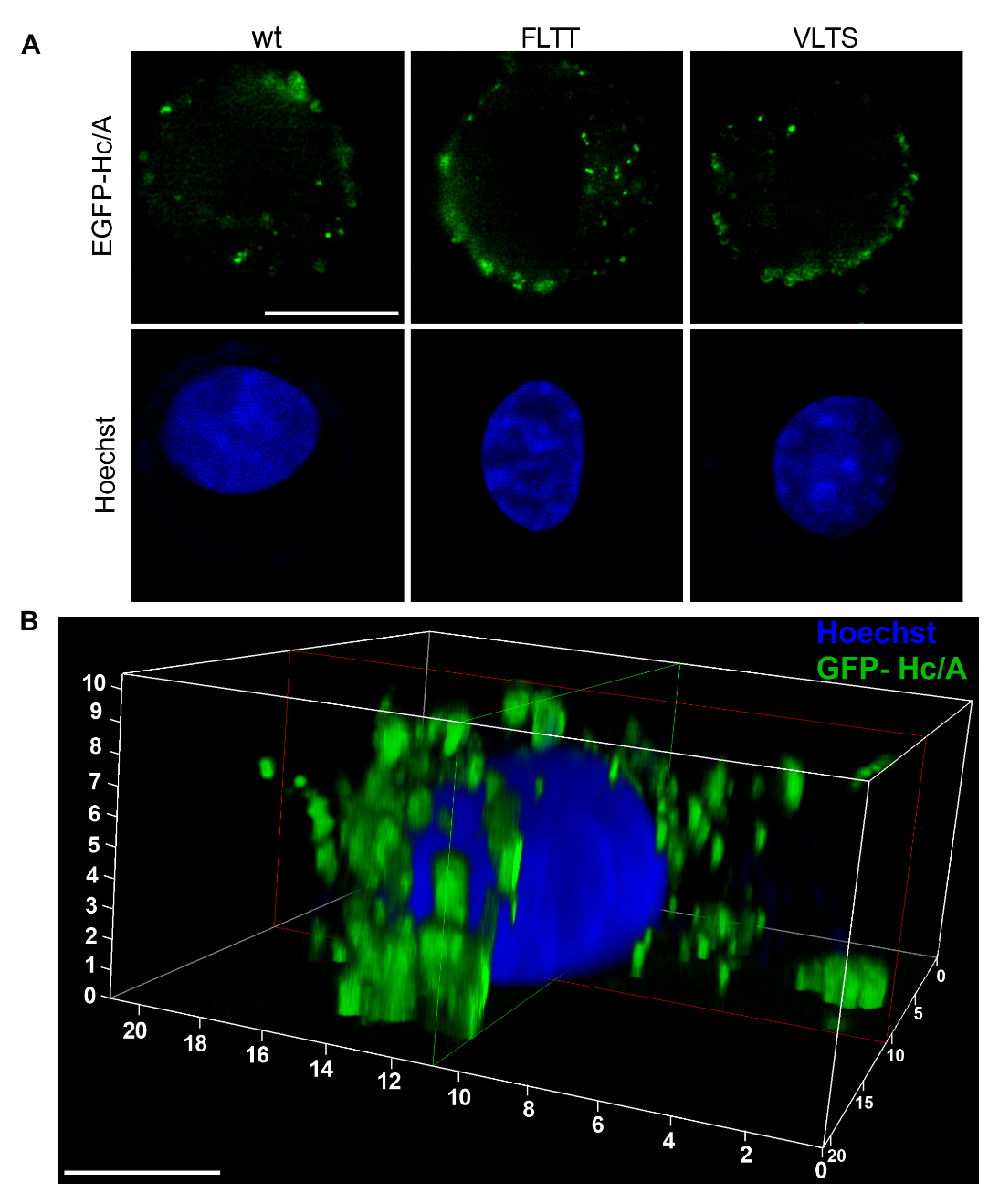


Figure S6. Toxin binding assay of EGFP - Hc/A wt, mutants of EGFP - FLTT and EGFP - VLTS in Neuro-2a cells. (A) Images were acquired by confocal microscopy in lighting mode (Leica). Scale bar 10 μm. (B) The 3D image was visualized using the confocal imaging software LAS × 3D analysis tool. Scale bar 5 μm.


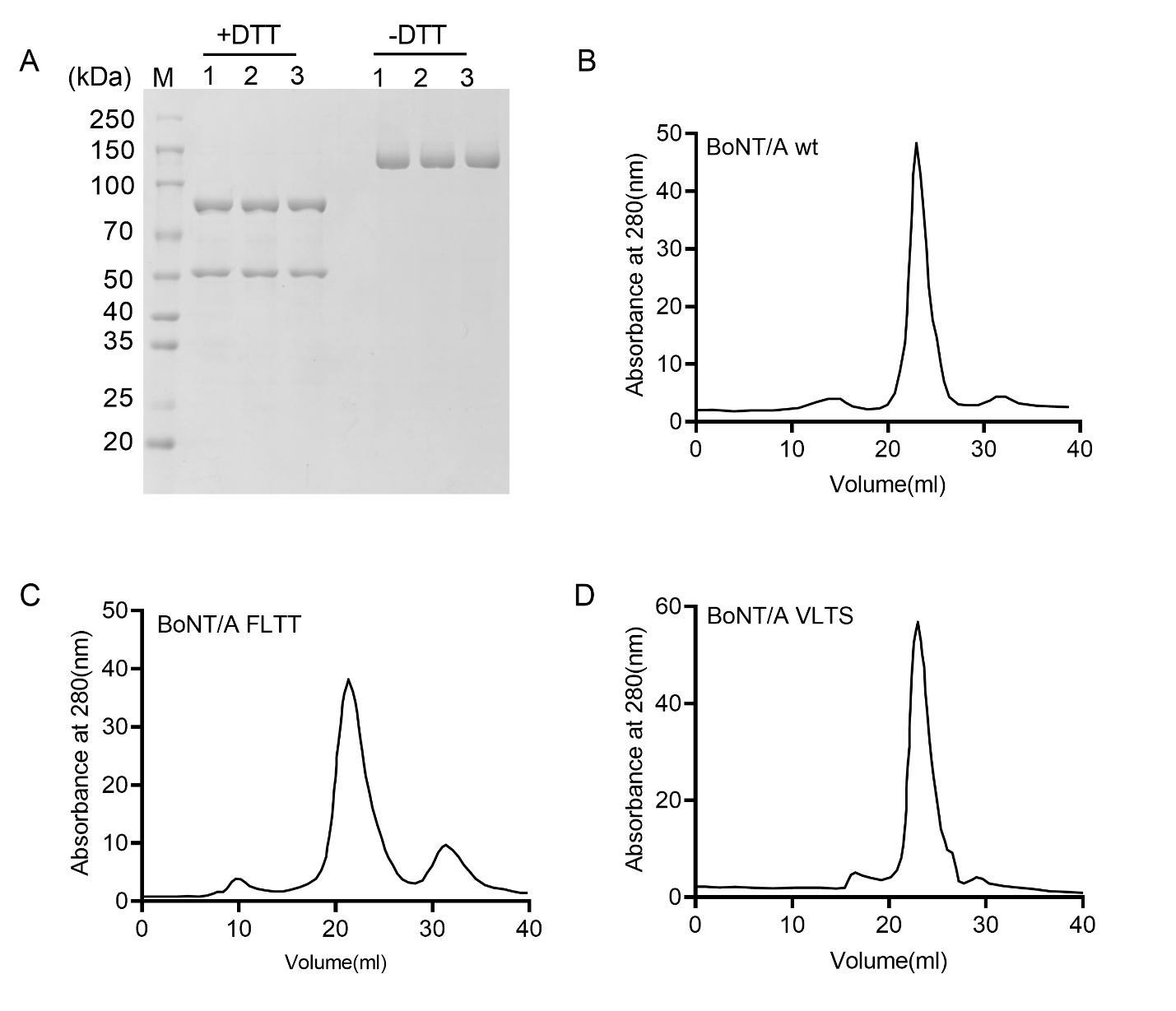


Figure S7. The SDS-PAGE analysis and size-exclusion chromatography (SEC) curves of purified full-length protein BoNT/A wt, FLTT and VLTS. (A) SDS-PAGE gel electrophoresis results of BoNT/A wt and mutants. 1: BoNT/A wt; 2: FLTT; 3: VLTS. (B-D) The SEC chromatograms of BoNT/A wt and mutants.


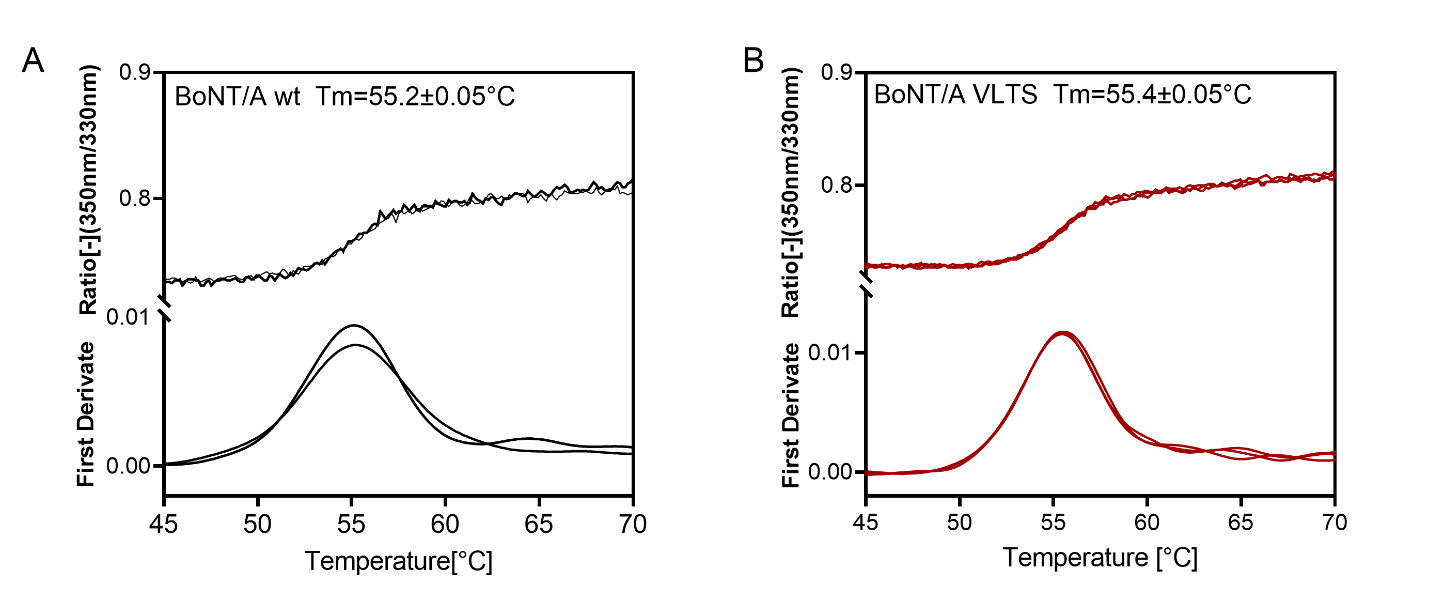
Figure S8. Thermal stability analysis of BoNT/A by differential scanning fluorimetry. (A) The thermal stability analysis of BoNT/A wt. (B) The thermal stability analysis of BoNT/A VLTS. Data are presented as mean ± SD (n≥2).


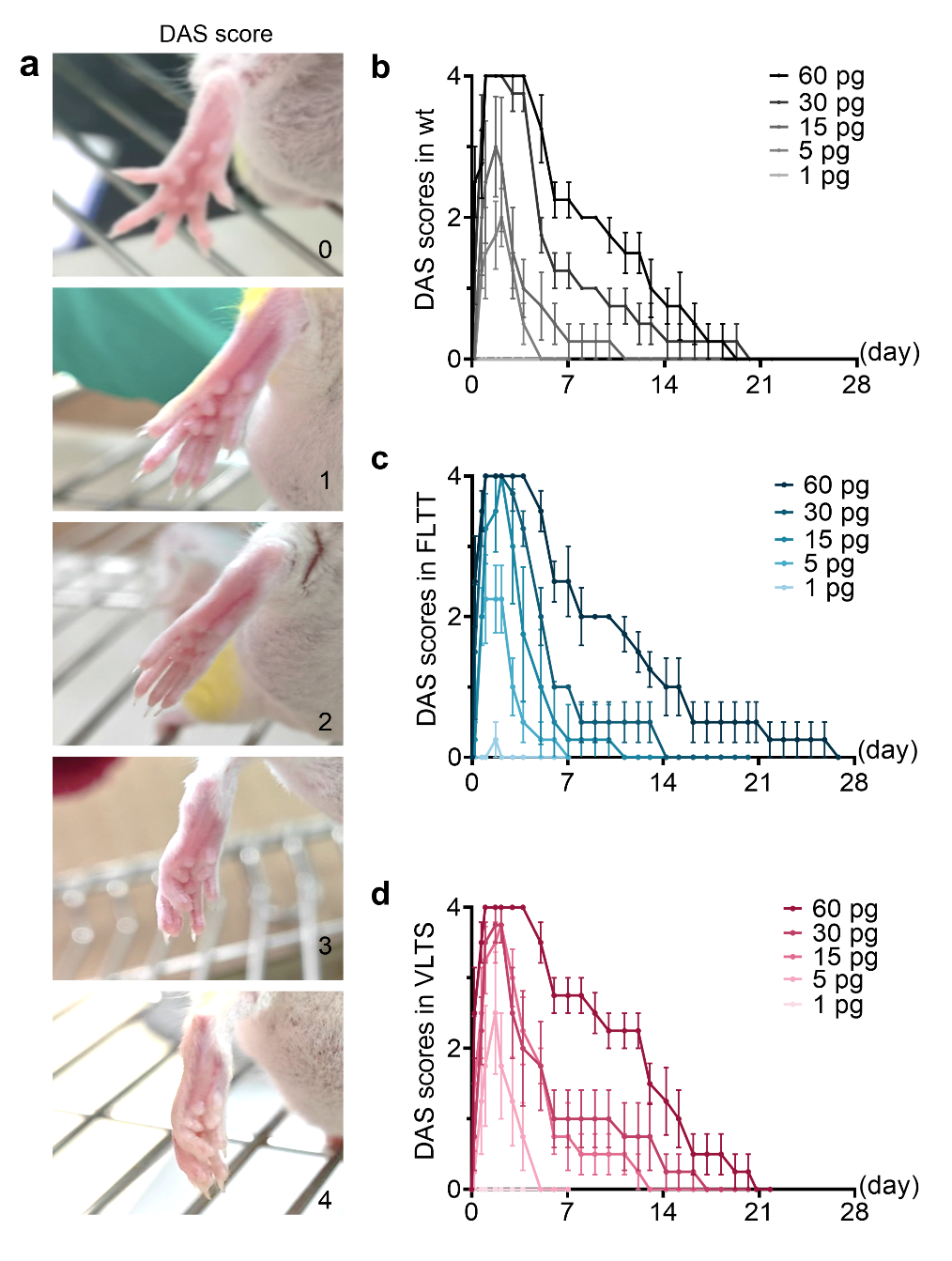


Figure S9. Toxin injection into mouse gastrocnemius muscle of the left hind limb showed a dose-dependent response on the DAS kinetics. (A) A comparison of DAS response patterns after botulinum toxin injection in mice. Corresponding DAS values are indicated in the lower right corner. Mice injected in the gastrocnemius muscle show a typical DAS response, with 0 indicating normal finger abduction and 4 indicating maximally reduced finger abduction. (B) BoNT/A wt (C) Engineered BoNT/A FLTT. (D) Engineered BoNT/A VLTS. All data are mean ± SEM of n=4.


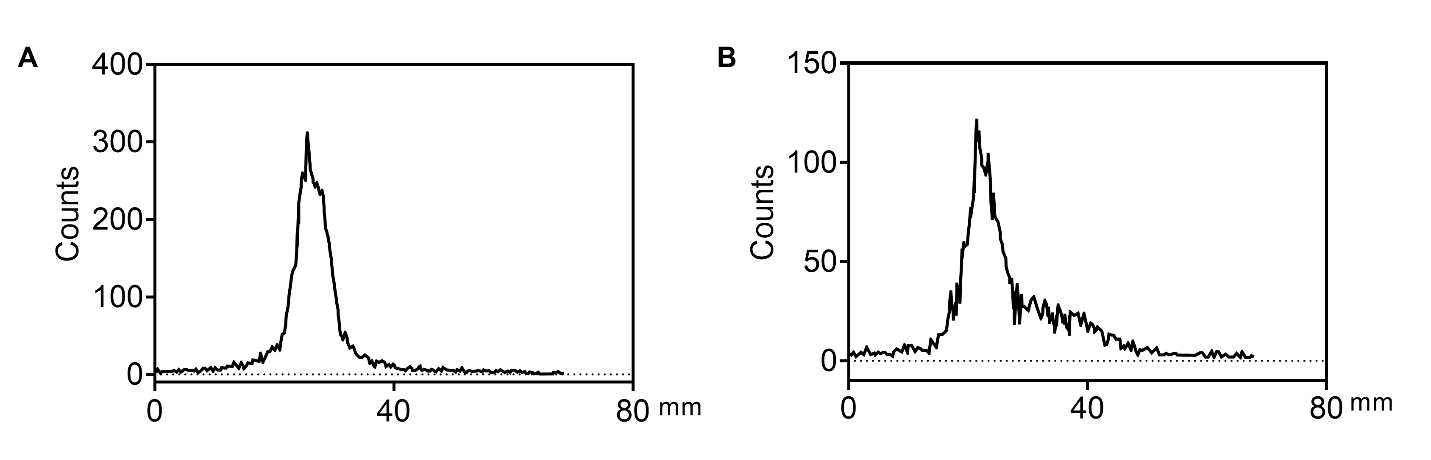
Figure S10. TLC analysis of ^68^Ga-NOTA-Hc/A. (A) TLC analysis of ^68^Ga-NOTA-Hc/A wt. The peak shows the ^68^Ga labeled Hc/A wt, (B) TLC analysis of ^68^Ga-NOTA-VLTS. The peak shows the ^68^Ga labeled VLTS.


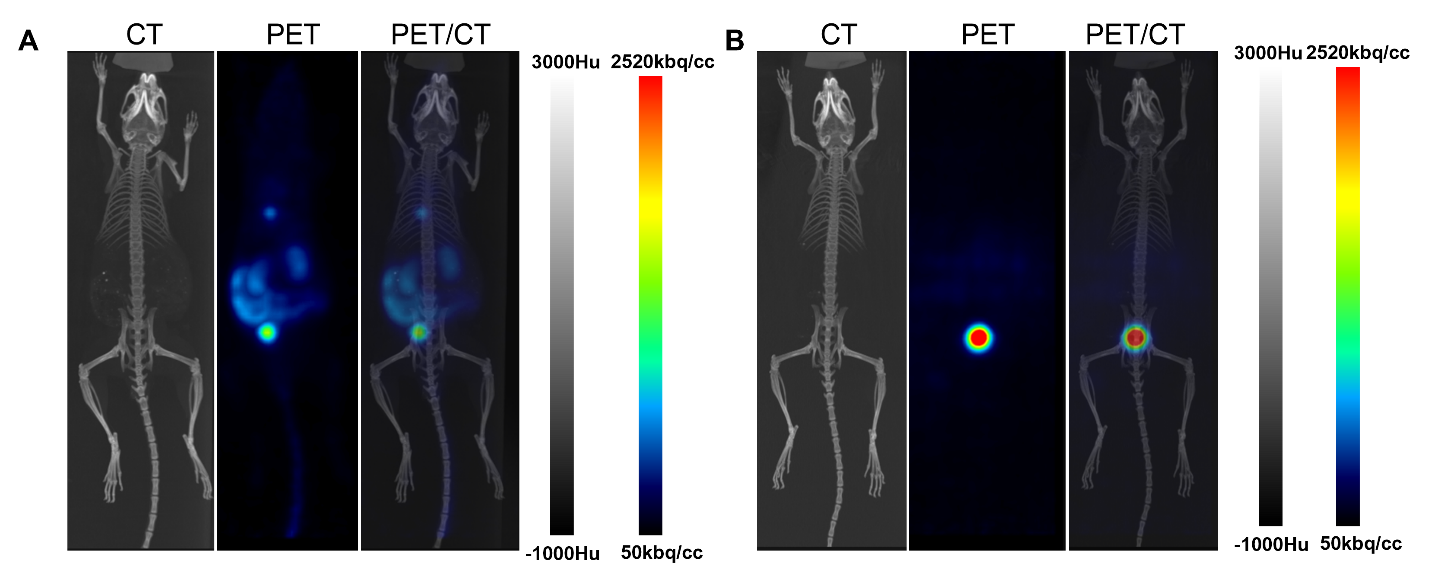


Figure S11. Mice were intravenously injected and intramuscularly injected free ^68^Ga. (A) Intravenous injection of ^68^Ga in mice. (B) Gastrocnemius muscle injection of ^68^Ga in mice. Free ^68^Ga rapidly completely diffused from the injection site, and was excreted *via* the kidneys.

| **Data collection and processing** | **open state of BoNT/A** | **semi-closed state of BoNT/A** |
| --- | --- | --- |
| Nominal magnification | 105000  300  60  -0.8~-1.8  0.831  33051  C1  1,441,792 | |
| Voltage(kV) |  |  |
| Electronexposure(e/Å2) |  |  |
| Defocus range(µ$m$) |  |  |
| Pixel size(Å) |  |  |
| Number of images |  |  |
| Symmetry imposed |  |  |
| Particles picked |  |  |
| Particles refined | 336,881 | 230734 |
| Map resolution(Å) | 2.85 | 2.80 |
| FSC threshold | 0.143 | 0.143 |
| Map sharpening B factor | -91.8 | -91.8 |
| R.m.s.d. Bondlengths(Å)  R.m.s.d. Bondangles( °) | 0.003  0.518 | 0.004  0.522 |
|  |  |  |
| **Validation** |  |  |
| MolProbity score | 1.93 | 1.94 |
| Clashscore | 12.12 | 12.43 |
| Poor rotamers(%) | 0 | 3 |
| Favored rotamers(%) | 99.65 | 99.38 |
| Favoured(%) | 95.18 | 95.11 |
| Outliers(%) | 0 | 0 |

Table S1. Cryo-EM data collection and refinement.

| BoNT/A | Distance (Å) | SV2A | Distance (Å) | SV2B | Distance (Å) | SV2C | Distance (Å) | | Glycosylate d SV2C |
| --- | --- | --- | --- | --- | --- | --- | --- | --- | --- |
| S 1142 N |  |  | 2.95 | F 519 O | 2.89 | F 562 O | 2.70 | F 562 O | |
| S 1142 O |  |  | 3.37 | T 518 OG1 | 2.88 | F 562 N | 2.71 | F 562 N | |
|  |  |  | 3.02 | F 519 N |  |  |  |  | |
| M 1144 O | 3.15 | S 574 |  |  |  |  |  |  | |
| T 1145 OG1 | 3.06 | N 573 N | 2.99 | N 516 N | 3.31 | F 557 O | 3.14 | N 559 N | |
| T 1146 N | 2.98 | L 571 O | 3.10 | F 514 O |  |  |  |  | |
| T 1146 O | 3.01 | L 571 N |  |  |  |  |  |  | |
| T 1146 OG1 |  |  | 3.00 | F 514 N |  |  |  |  | |
| Y 1149 OH |  |  |  |  | 2.87 | N 559 OD1 | 2.59 | N 559 OD1 | |
| R 1156 N |  |  |  |  |  |  | 2.70 | F 562 0 | |
| R 1156 O |  |  |  |  |  |  | 2.71 | F 562 N | |
| R 1156 NH1 |  |  |  |  | 3.09 | D 543 OD2 |  |  | |
| R 1294 NH1 |  |  | 2.97 | E 476 OE2 |  |  | 2.88 | D 539 O | |
| R 1294 NH2 | 3.34 | E 533 OE2 | 3.07 | E 476 OE2 | 2.83 | D 539 O | 2.91 | D 539 O | |
| R 1294 NE |  |  |  |  | 2.73 | D 539 O |  |  | |

Table S2. Hydrogen bonds between BoNT/A and SV2s.

| Residue Index | | Sequence | Binding site probability | | | Residue Index | Sequence | Binding site probability | | |
| --- | --- | --- | --- | --- | --- | --- | --- | --- | --- | --- |
|  |  | | SMTT | FLTT | VLTS |  |  | SMTT | FLTT | VLTS |
| 1 | M | | 0.215 | 0.209 | 0.209 | 653 | V | 0.026 | 0.033 | 0.033 |
| 2 | P | | 0.294 | 0.309 | 0.309 | 654 | G | 0.058 | 0.057 | 0.057 |
| 3 | F | | 0.062 | 0.07 | 0.07 | 655 | A | 0.063 | 0.08 | 0.08 |
| 4 | V | | 0.029 | 0.033 | 0.032 | 656 | L | 0.014 | 0.013 | 0.013 |
| 5 | N | | 0.095 | 0.123 | 0.123 | 657 | I | 0.057 | 0.047 | 0.047 |
| 6 | K | | 0.125 | 0.154 | 0.154 | 658 | F | 0.1 | 0.078 | 0.078 |
| 7 | Q | | 0.075 | 0.092 | 0.092 | 659 | S | 0.092 | 0.073 | 0.073 |
| 8 | F | | 0.06 | 0.083 | 0.083 | 660 | G | 0.021 | 0.019 | 0.019 |
| 9 | N | | 0.061 | 0.067 | 0.067 | 661 | A | 0.005 | 0.004 | 0.004 |
| 10 | Y | | 0.061 | 0.061 | 0.06 | 662 | V | 0.107 | 0.11 | 0.11 |
| 11 | K | | 0.144 | 0.143 | 0.143 | 663 | I | 0.032 | 0.042 | 0.042 |
| 12 | D | | 0.147 | 0.138 | 0.138 | 664 | L | 0.007 | 0.009 | 0.009 |
| 13 | P | | 0.326 | 0.303 | 0.304 | 665 | L | 0.013 | 0.011 | 0.011 |
| 14 | V | | 0.445 | 0.387 | 0.387 | 666 | E | 0.054 | 0.036 | 0.036 |
| 15 | N | | 0.224 | 0.224 | 0.224 | 667 | F | 0.047 | 0.032 | 0.032 |
| 16 | G | | 0.048 | 0.028 | 0.028 | 668 | I | 0.048 | 0.042 | 0.043 |
| 17 | V | | 0.109 | 0.089 | 0.089 | 669 | P | 0.021 | 0.019 | 0.019 |
| 18 | D | | 0.18 | 0.174 | 0.174 | 670 | E | 0.09 | 0.104 | 0.105 |
| 19 | I | | 0.019 | 0.019 | 0.019 | 671 | I | 0.04 | 0.024 | 0.024 |
| 20 | A | | 0.012 | 0.01 | 0.01 | 672 | A | 0.055 | 0.061 | 0.061 |
| 21 | Y | | 0.278 | 0.191 | 0.19 | 673 | I | 0.031 | 0.024 | 0.024 |
| 22 | I | | 0.005 | 0.004 | 0.004 | 674 | P | 0.047 | 0.044 | 0.044 |
| 23 | K | | 0.058 | 0.051 | 0.051 | 675 | V | 0.112 | 0.136 | 0.136 |
| 24 | I | | 0.035 | 0.028 | 0.028 | 676 | L | 0.031 | 0.063 | 0.063 |
| 25 | P | | 0.016 | 0.012 | 0.012 | 677 | G | 0.047 | 0.066 | 0.066 |
| 26 | N | | 0.04 | 0.069 | 0.069 | 678 | T | 0.066 | 0.091 | 0.091 |
| 27 | A | | 0.297 | 0.322 | 0.323 | 679 | F | 0.016 | 0.019 | 0.019 |
| 28 | G | | 0.139 | 0.131 | 0.132 | 680 | A | 0.093 | 0.14 | 0.14 |
| 29 | Q | | 0.118 | 0.105 | 0.106 | 681 | L | 0.048 | 0.078 | 0.078 |
| 30 | M | | 0.059 | 0.061 | 0.062 | 682 | V | 0.134 | 0.197 | 0.197 |
| 31 | Q | | 0.246 | 0.314 | 0.314 | 683 | S | 0.255 | 0.312 | 0.312 |
| 32 | P | | 0.18 | 0.176 | 0.176 | 684 | Y | 0.168 | 0.238 | 0.238 |
| 33 | V | | 0.029 | 0.032 | 0.032 | 685 | I | 0.499 | 0.53 | 0.53 |
| 34 | K | | 0.044 | 0.038 | 0.038 | 686 | A | 0.414 | 0.385 | 0.386 |
| 35 | A | | 0 | 0 | 0 | 687 | N | 0.142 | 0.193 | 0.193 |
| 36 | F | | 0.001 | 0.001 | 0.001 | 688 | K | 0.28 | 0.248 | 0.248 |
| 37 | K | | 0.021 | 0.028 | 0.028 | 689 | V | 0.139 | 0.127 | 0.128 |
| 38 | I | | 0.005 | 0.006 | 0.006 | 690 | L | 0.095 | 0.077 | 0.077 |
| 39 | H | | 0.029 | 0.035 | 0.035 | 691 | T | 0.015 | 0.016 | 0.016 |
| 40 | N | | 0.029 | 0.032 | 0.032 | 692 | V | 0.085 | 0.085 | 0.085 |
| 41 | K | | 0.006 | 0.007 | 0.007 | 693 | Q | 0.06 | 0.058 | 0.059 |
| 42 | I | | 0.001 | 0.001 | 0.001 | 694 | T | 0.008 | 0.008 | 0.008 |
| 43 | W | | 0.001 | 0.004 | 0.004 | 695 | I | 0.038 | 0.037 | 0.037 |
| 44 | V | | 0 | 0 | 0 | 696 | D | 0.082 | 0.087 | 0.087 |
| 45 | I | | 0 | 0 | 0 | 697 | N | 0.015 | 0.016 | 0.016 |
| 46 | P | | 0 | 0 | 0 | 698 | A | 0.007 | 0.008 | 0.008 |
| 47 | E | | 0.058 | 0.093 | 0.092 | 699 | L | 0.029 | 0.038 | 0.038 |
| 48 | R | | 0.024 | 0.037 | 0.037 | 700 | S | 0.044 | 0.054 | 0.054 |
| 49 | D | | 0.014 | 0.009 | 0.009 | 701 | K | 0.014 | 0.015 | 0.015 |
| 50 | T | | 0.031 | 0.036 | 0.036 | 702 | R | 0.036 | 0.049 | 0.05 |
| 51 | F | | 0.053 | 0.077 | 0.077 | 703 | N | 0.099 | 0.121 | 0.121 |
| 52 | T | | 0.01 | 0.01 | 0.01 | 704 | E | 0.049 | 0.053 | 0.053 |
| 53 | N | | 0.05 | 0.063 | 0.063 | 705 | K | 0.009 | 0.011 | 0.011 |
| 54 | P | | 0.148 | 0.121 | 0.121 | 706 | W | 0.067 | 0.11 | 0.11 |
| 55 | E | | 0.35 | 0.349 | 0.35 | 707 | D | 0.051 | 0.059 | 0.059 |
| 56 | E | | 0.128 | 0.047 | 0.047 | 708 | E | 0.036 | 0.039 | 0.039 |
| 57 | G | | 0.031 | 0.037 | 0.037 | 709 | V | 0.003 | 0.003 | 0.003 |
| 58 | D | | 0.031 | 0.043 | 0.043 | 710 | Y | 0.037 | 0.041 | 0.042 |
| 59 | L | | 0.039 | 0.029 | 0.029 | 711 | K | 0.044 | 0.041 | 0.041 |
| 60 | N | | 0.028 | 0.035 | 0.035 | 712 | Y | 0.025 | 0.02 | 0.02 |
| 61 | P | | 0.045 | 0.059 | 0.059 | 713 | I | 0.004 | 0.003 | 0.003 |
| 62 | P | | 0.028 | 0.044 | 0.044 | 714 | V | 0.01 | 0.008 | 0.008 |
| 63 | P | | 0.048 | 0.076 | 0.076 | 715 | T | 0.026 | 0.022 | 0.022 |
| 64 | E | | 0.051 | 0.07 | 0.07 | 716 | N | 0.006 | 0.005 | 0.005 |
| 65 | A | | 0.039 | 0.044 | 0.044 | 717 | W | 0.005 | 0.004 | 0.004 |
| 66 | K | | 0.013 | 0.016 | 0.016 | 718 | L | 0.006 | 0.006 | 0.006 |
| 67 | Q | | 0.026 | 0.032 | 0.032 | 719 | A | 0.005 | 0.005 | 0.005 |
| 68 | V | | 0.018 | 0.018 | 0.018 | 720 | K | 0.004 | 0.004 | 0.004 |
| 69 | P | | 0.025 | 0.031 | 0.031 | 721 | V | 0.002 | 0.002 | 0.002 |
| 70 | V | | 0.064 | 0.042 | 0.042 | 722 | N | 0.004 | 0.005 | 0.005 |
| 71 | S | | 0.003 | 0.003 | 0.003 | 723 | T | 0.002 | 0.002 | 0.002 |
| 72 | Y | | 0.114 | 0.102 | 0.102 | 724 | Q | 0.001 | 0.001 | 0.001 |
| 73 | Y | | 0.038 | 0.033 | 0.033 | 725 | I | 0.001 | 0.001 | 0.001 |
| 74 | D | | 0.046 | 0.031 | 0.031 | 726 | D | 0.006 | 0.006 | 0.006 |
| 75 | S | | 0.076 | 0.077 | 0.077 | 727 | L | 0.004 | 0.005 | 0.005 |
| 76 | T | | 0.086 | 0.085 | 0.085 | 728 | I | 0.001 | 0 | 0 |
| 77 | Y | | 0.107 | 0.072 | 0.072 | 729 | R | 0.001 | 0.001 | 0.001 |
| 78 | L | | 0.007 | 0.02 | 0.02 | 730 | K | 0.003 | 0.004 | 0.004 |
| 79 | S | | 0.087 | 0.096 | 0.095 | 731 | K | 0.003 | 0.003 | 0.003 |
| 80 | T | | 0.068 | 0.066 | 0.066 | 732 | M | 0 | 0 | 0 |
| 81 | D | | 0.037 | 0.044 | 0.044 | 733 | K | 0.002 | 0.002 | 0.002 |
| 82 | N | | 0.092 | 0.09 | 0.09 | 734 | E | 0.004 | 0.003 | 0.003 |
| 83 | E | | 0.048 | 0.047 | 0.047 | 735 | A | 0.006 | 0.004 | 0.004 |
| 84 | K | | 0.017 | 0.029 | 0.029 | 736 | L | 0.001 | 0.001 | 0.001 |
| 85 | D | | 0.018 | 0.024 | 0.024 | 737 | E | 0.002 | 0.002 | 0.002 |
| 86 | N | | 0.047 | 0.043 | 0.043 | 738 | N | 0.007 | 0.007 | 0.007 |
| 87 | Y | | 0.004 | 0.002 | 0.002 | 739 | Q | 0.005 | 0.007 | 0.007 |
| 88 | L | | 0 | 0.001 | 0.001 | 740 | A | 0.004 | 0.005 | 0.005 |
| 89 | K | | 0.045 | 0.046 | 0.046 | 741 | E | 0.008 | 0.01 | 0.01 |
| 90 | G | | 0.008 | 0.004 | 0.004 | 742 | A | 0.018 | 0.02 | 0.02 |
| 91 | V | | 0.002 | 0 | 0 | 743 | T | 0.004 | 0.004 | 0.004 |
| 92 | T | | 0.014 | 0.017 | 0.017 | 744 | K | 0.008 | 0.011 | 0.011 |
| 93 | K | | 0.095 | 0.089 | 0.088 | 745 | A | 0.011 | 0.017 | 0.017 |
| 94 | L | | 0.003 | 0.002 | 0.002 | 746 | I | 0.058 | 0.064 | 0.064 |
| 95 | F | | 0.006 | 0.005 | 0.005 | 747 | I | 0.006 | 0.009 | 0.009 |
| 96 | E | | 0.076 | 0.08 | 0.08 | 748 | N | 0.019 | 0.03 | 0.03 |
| 97 | R | | 0.045 | 0.04 | 0.04 | 749 | Y | 0.087 | 0.101 | 0.102 |
| 98 | I | | 0.007 | 0.004 | 0.004 | 750 | Q | 0.128 | 0.157 | 0.157 |
| 99 | Y | | 0.066 | 0.065 | 0.065 | 751 | Y | 0.042 | 0.06 | 0.06 |
| 100 | S | | 0.186 | 0.17 | 0.17 | 752 | N | 0.092 | 0.119 | 0.119 |
| 101 | T | | 0.016 | 0.013 | 0.013 | 753 | Q | 0.142 | 0.257 | 0.258 |
| 102 | D | | 0.169 | 0.136 | 0.136 | 754 | Y | 0.214 | 0.261 | 0.262 |
| 103 | L | | 0.003 | 0.006 | 0.006 | 755 | T | 0.215 | 0.229 | 0.229 |
| 104 | G | | 0.001 | 0.001 | 0.001 | 756 | E | 0.144 | 0.154 | 0.154 |
| 105 | R | | 0.073 | 0.065 | 0.065 | 757 | E | 0.15 | 0.158 | 0.159 |
| 106 | M | | 0.01 | 0.013 | 0.013 | 758 | E | 0.082 | 0.098 | 0.098 |
| 107 | L | | 0.001 | 0.002 | 0.002 | 759 | K | 0.095 | 0.107 | 0.107 |
| 108 | L | | 0.004 | 0.004 | 0.004 | 760 | N | 0.122 | 0.133 | 0.133 |
| 109 | T | | 0.019 | 0.023 | 0.023 | 761 | N | 0.149 | 0.163 | 0.163 |
| 110 | S | | 0.008 | 0.009 | 0.009 | 762 | I | 0.117 | 0.144 | 0.145 |
| 111 | I | | 0.002 | 0.002 | 0.002 | 763 | N | 0.172 | 0.19 | 0.19 |
| 112 | V | | 0.006 | 0.009 | 0.009 | 764 | F | 0.07 | 0.093 | 0.094 |
| 113 | R | | 0.018 | 0.024 | 0.024 | 765 | N | 0.086 | 0.106 | 0.106 |
| 114 | G | | 0.001 | 0.001 | 0.001 | 766 | I | 0.023 | 0.031 | 0.031 |
| 115 | I | | 0.003 | 0.005 | 0.005 | 767 | D | 0.103 | 0.126 | 0.126 |
| 116 | P | | 0.001 | 0.001 | 0.001 | 768 | D | 0.081 | 0.091 | 0.092 |
| 117 | F | | 0.011 | 0.011 | 0.011 | 769 | L | 0.013 | 0.013 | 0.013 |
| 118 | W | | 0.007 | 0.013 | 0.013 | 770 | S | 0.029 | 0.039 | 0.039 |
| 119 | G | | 0.136 | 0.075 | 0.075 | 771 | S | 0.155 | 0.165 | 0.165 |
| 120 | G | | 0.294 | 0.196 | 0.196 | 772 | K | 0.151 | 0.146 | 0.146 |
| 121 | S | | 0.225 | 0.202 | 0.203 | 773 | L | 0.011 | 0.016 | 0.016 |
| 122 | T | | 0.428 | 0.446 | 0.447 | 774 | N | 0.04 | 0.04 | 0.04 |
| 123 | I | | 0.294 | 0.312 | 0.313 | 775 | E | 0.172 | 0.161 | 0.161 |
| 124 | D | | 0.158 | 0.257 | 0.257 | 776 | S | 0.072 | 0.09 | 0.09 |
| 125 | T | | 0.088 | 0.087 | 0.087 | 777 | I | 0.005 | 0.004 | 0.004 |
| 126 | E | | 0.086 | 0.119 | 0.119 | 778 | N | 0.049 | 0.053 | 0.053 |
| 127 | L | | 0.013 | 0.025 | 0.025 | 779 | K | 0.377 | 0.413 | 0.412 |
| 128 | K | | 0.172 | 0.182 | 0.182 | 780 | A | 0.002 | 0.002 | 0.002 |
| 129 | V | | 0.04 | 0.04 | 0.04 | 781 | M | 0.001 | 0.001 | 0.001 |
| 130 | I | | 0.043 | 0.026 | 0.026 | 782 | I | 0.089 | 0.091 | 0.091 |
| 131 | D | | 0.196 | 0.149 | 0.15 | 783 | N | 0.193 | 0.177 | 0.177 |
| 132 | T | | 0.024 | 0.013 | 0.013 | 784 | I | 0 | 0 | 0 |
| 133 | N | | 0.002 | 0.002 | 0.002 | 785 | N | 0.002 | 0.003 | 0.003 |
| 134 | C | | 0.004 | 0.004 | 0.004 | 786 | K | 0.036 | 0.035 | 0.035 |
| 135 | I | | 0.007 | 0.008 | 0.008 | 787 | F | 0.001 | 0.001 | 0.001 |
| 136 | N | | 0.018 | 0.02 | 0.02 | 788 | L | 0 | 0 | 0 |
| 137 | V | | 0.01 | 0.004 | 0.004 | 789 | N | 0.014 | 0.014 | 0.014 |
| 138 | I | | 0.145 | 0.15 | 0.15 | 790 | Q | 0.02 | 0.013 | 0.013 |
| 139 | Q | | 0.106 | 0.081 | 0.081 | 791 | C | 0.002 | 0.002 | 0.002 |
| 140 | P | | 0.265 | 0.213 | 0.213 | 792 | S | 0.001 | 0.001 | 0.001 |
| 141 | D | | 0.142 | 0.132 | 0.132 | 793 | V | 0.009 | 0.009 | 0.009 |
| 142 | G | | 0.082 | 0.09 | 0.09 | 794 | S | 0.002 | 0.002 | 0.002 |
| 143 | S | | 0.123 | 0.104 | 0.104 | 795 | Y | 0.019 | 0.018 | 0.018 |
| 144 | Y | | 0.133 | 0.168 | 0.169 | 796 | L | 0.006 | 0.006 | 0.006 |
| 145 | R | | 0.112 | 0.088 | 0.088 | 797 | M | 0.009 | 0.007 | 0.007 |
| 146 | S | | 0.026 | 0.025 | 0.025 | 798 | N | 0.015 | 0.012 | 0.012 |
| 147 | E | | 0.023 | 0.018 | 0.018 | 799 | S | 0.053 | 0.041 | 0.041 |
| 148 | E | | 0.01 | 0.011 | 0.011 | 800 | M | 0.016 | 0.014 | 0.014 |
| 149 | L | | 0.003 | 0.004 | 0.004 | 801 | I | 0.013 | 0.026 | 0.026 |
| 150 | N | | 0.001 | 0.001 | 0.001 | 802 | P | 0.008 | 0.037 | 0.037 |
| 151 | L | | 0.001 | 0 | 0 | 803 | Y | 0.082 | 0.097 | 0.098 |
| 152 | V | | 0 | 0 | 0 | 804 | G | 0.004 | 0.003 | 0.003 |
| 153 | I | | 0 | 0 | 0 | 805 | V | 0.013 | 0.03 | 0.03 |
| 154 | I | | 0.001 | 0.001 | 0.001 | 806 | K | 0.074 | 0.115 | 0.116 |
| 155 | G | | 0 | 0 | 0 | 807 | R | 0.123 | 0.15 | 0.15 |
| 156 | P | | 0.009 | 0.005 | 0.005 | 808 | L | 0.014 | 0.02 | 0.02 |
| 157 | S | | 0.006 | 0.008 | 0.008 | 809 | E | 0.013 | 0.014 | 0.014 |
| 158 | A | | 0.004 | 0.005 | 0.005 | 810 | D | 0.059 | 0.077 | 0.077 |
| 159 | D | | 0.002 | 0.001 | 0.001 | 811 | F | 0.014 | 0.02 | 0.02 |
| 160 | I | | 0.007 | 0.005 | 0.005 | 812 | D | 0.011 | 0.015 | 0.015 |
| 161 | I | | 0.007 | 0.005 | 0.005 | 813 | A | 0.029 | 0.027 | 0.027 |
| 162 | Q | | 0.006 | 0.003 | 0.003 | 814 | S | 0.054 | 0.07 | 0.071 |
| 163 | F | | 0.038 | 0.008 | 0.008 | 815 | L | 0.019 | 0.024 | 0.024 |
| 164 | E | | 0.057 | 0.033 | 0.033 | 816 | K | 0.021 | 0.031 | 0.031 |
| 165 | C | | 0.022 | 0.007 | 0.007 | 817 | D | 0.092 | 0.124 | 0.124 |
| 166 | K | | 0.114 | 0.049 | 0.049 | 818 | A | 0.021 | 0.027 | 0.027 |
| 167 | S | | 0.003 | 0.002 | 0.002 | 819 | L | 0.008 | 0.009 | 0.009 |
| 168 | F | | 0.034 | 0.025 | 0.025 | 820 | L | 0.082 | 0.109 | 0.109 |
| 169 | G | | 0.126 | 0.107 | 0.107 | 821 | K | 0.115 | 0.152 | 0.152 |
| 170 | H | | 0.396 | 0.384 | 0.384 | 822 | Y | 0.03 | 0.063 | 0.063 |
| 171 | E | | 0.583 | 0.634 | 0.635 | 823 | I | 0.11 | 0.129 | 0.13 |
| 172 | V | | 0.496 | 0.523 | 0.524 | 824 | Y | 0.401 | 0.471 | 0.472 |
| 173 | L | | 0.218 | 0.173 | 0.173 | 825 | D | 0.125 | 0.17 | 0.17 |
| 174 | N | | 0.274 | 0.268 | 0.269 | 826 | N | 0.051 | 0.064 | 0.064 |
| 175 | L | | 0.022 | 0.012 | 0.012 | 827 | R | 0.641 | 0.646 | 0.646 |
| 176 | T | | 0.011 | 0.007 | 0.007 | 828 | G | 0.545 | 0.579 | 0.579 |
| 177 | R | | 0.065 | 0.089 | 0.089 | 829 | T | 0.243 | 0.236 | 0.236 |
| 178 | N | | 0.082 | 0.07 | 0.07 | 830 | L | 0.086 | 0.091 | 0.091 |
| 179 | G | | 0.008 | 0.008 | 0.008 | 831 | I | 0.5 | 0.53 | 0.53 |
| 180 | Y | | 0.035 | 0.017 | 0.017 | 832 | G | 0.23 | 0.25 | 0.251 |
| 181 | G | | 0 | 0 | 0 | 833 | Q | 0.122 | 0.109 | 0.109 |
| 182 | S | | 0.001 | 0.001 | 0.001 | 834 | V | 0.124 | 0.151 | 0.151 |
| 183 | T | | 0 | 0.001 | 0.001 | 835 | D | 0.044 | 0.049 | 0.049 |
| 184 | Q | | 0.003 | 0.001 | 0.001 | 836 | R | 0.05 | 0.049 | 0.049 |
| 185 | Y | | 0.001 | 0.001 | 0.001 | 837 | L | 0.045 | 0.048 | 0.048 |
| 186 | I | | 0.002 | 0.001 | 0.001 | 838 | K | 0.071 | 0.09 | 0.09 |
| 187 | R | | 0.003 | 0.001 | 0.001 | 839 | D | 0.032 | 0.043 | 0.043 |
| 188 | F | | 0.013 | 0.001 | 0.001 | 840 | K | 0.044 | 0.049 | 0.049 |
| 189 | S | | 0.002 | 0.001 | 0.001 | 841 | V | 0.031 | 0.04 | 0.04 |
| 190 | P | | 0.002 | 0.001 | 0.001 | 842 | N | 0.029 | 0.038 | 0.038 |
| 191 | D | | 0.011 | 0.007 | 0.007 | 843 | N | 0.027 | 0.036 | 0.036 |
| 192 | F | | 0.085 | 0.035 | 0.035 | 844 | T | 0.049 | 0.062 | 0.062 |
| 193 | T | | 0.009 | 0.005 | 0.005 | 845 | L | 0.03 | 0.045 | 0.045 |
| 194 | F | | 0.024 | 0.021 | 0.021 | 846 | S | 0.015 | 0.026 | 0.026 |
| 195 | G | | 0.007 | 0.007 | 0.007 | 847 | T | 0.066 | 0.104 | 0.104 |
| 196 | F | | 0.004 | 0.003 | 0.003 | 848 | D | 0.055 | 0.066 | 0.066 |
| 197 | E | | 0.012 | 0.012 | 0.012 | 849 | I | 0.093 | 0.111 | 0.111 |
| 198 | E | | 0.005 | 0.004 | 0.004 | 850 | P | 0.163 | 0.162 | 0.162 |
| 199 | S | | 0.001 | 0.001 | 0.001 | 851 | F | 0.061 | 0.062 | 0.062 |
| 200 | L | | 0.011 | 0.008 | 0.008 | 852 | Q | 0.197 | 0.188 | 0.188 |
| 201 | E | | 0.028 | 0.022 | 0.022 | 853 | L | 0.022 | 0.022 | 0.022 |
| 202 | V | | 0.004 | 0.004 | 0.004 | 854 | S | 0.126 | 0.116 | 0.117 |
| 203 | D | | 0.061 | 0.054 | 0.054 | 855 | K | 0.117 | 0.122 | 0.122 |
| 204 | T | | 0.066 | 0.055 | 0.055 | 856 | Y | 0.04 | 0.041 | 0.041 |
| 205 | N | | 0.05 | 0.049 | 0.049 | 857 | V | 0.01 | 0.01 | 0.01 |
| 206 | P | | 0.06 | 0.119 | 0.119 | 858 | D | 0.074 | 0.061 | 0.061 |
| 207 | L | | 0.097 | 0.085 | 0.085 | 859 | N | 0.033 | 0.03 | 0.03 |
| 208 | L | | 0.085 | 0.07 | 0.07 | 860 | Q | 0.193 | 0.192 | 0.192 |
| 209 | G | | 0.04 | 0.046 | 0.046 | 861 | R | 0.023 | 0.019 | 0.019 |
| 210 | A | | 0.007 | 0.008 | 0.008 | 862 | L | 0.004 | 0.004 | 0.004 |
| 211 | G | | 0.005 | 0.006 | 0.006 | 863 | L | 0.135 | 0.123 | 0.123 |
| 212 | K | | 0.027 | 0.026 | 0.026 | 864 | S | 0.1 | 0.087 | 0.087 |
| 213 | F | | 0.007 | 0.01 | 0.01 | 865 | T | 0.008 | 0.007 | 0.007 |
| 214 | A | | 0.001 | 0.001 | 0.001 | 866 | F | 0.04 | 0.041 | 0.041 |
| 215 | T | | 0.003 | 0.003 | 0.003 | 867 | T | 0.134 | 0.12 | 0.12 |
| 216 | D | | 0.001 | 0.001 | 0.001 | 868 | E | 0.067 | 0.048 | 0.048 |
| 217 | P | | 0.005 | 0.002 | 0.002 | 869 | Y | 0.012 | 0.01 | 0.01 |
| 218 | A | | 0.001 | 0.001 | 0.001 | 870 | I | 0.116 | 0.086 | 0.086 |
| 219 | V | | 0.003 | 0.003 | 0.003 | 871 | K | 0.21 | 0.169 | 0.169 |
| 220 | T | | 0.024 | 0.01 | 0.01 | 872 | N | 0.013 | 0.007 | 0.007 |
| 221 | L | | 0.004 | 0.001 | 0.001 | 873 | I | 0.047 | 0.02 | 0.02 |
| 222 | A | | 0.001 | 0.001 | 0.001 | 874 | I | 0.015 | 0.006 | 0.006 |
| 223 | H | | 0.013 | 0.012 | 0.012 | 875 | N | 0.009 | 0.005 | 0.005 |
| 224 | E | | 0.026 | 0.008 | 0.008 | 876 | T | 0.04 | 0.011 | 0.011 |
| 225 | L | | 0.001 | 0.001 | 0.001 | 877 | S | 0.015 | 0.007 | 0.007 |
| 226 | I | | 0.002 | 0.003 | 0.003 | 878 | I | 0.02 | 0.009 | 0.009 |
| 227 | H | | 0.051 | 0.033 | 0.033 | 879 | L | 0.009 | 0.003 | 0.003 |
| 228 | A | | 0.002 | 0.001 | 0.001 | 880 | N | 0.004 | 0.002 | 0.002 |
| 229 | G | | 0.001 | 0.001 | 0.001 | 881 | L | 0.011 | 0.004 | 0.004 |
| 230 | H | | 0.005 | 0.01 | 0.01 | 882 | R | 0.023 | 0.012 | 0.012 |
| 231 | R | | 0.032 | 0.006 | 0.006 | 883 | Y | 0.071 | 0.057 | 0.057 |
| 232 | L | | 0.001 | 0 | 0 | 884 | E | 0.058 | 0.057 | 0.057 |
| 233 | Y | | 0.015 | 0.02 | 0.02 | 885 | S | 0.093 | 0.083 | 0.084 |
| 234 | G | | 0.011 | 0.011 | 0.011 | 886 | N | 0.096 | 0.079 | 0.079 |
| 235 | I | | 0.03 | 0.054 | 0.054 | 887 | H | 0.386 | 0.255 | 0.256 |
| 236 | A | | 0.007 | 0.008 | 0.008 | 888 | L | 0.121 | 0.063 | 0.063 |
| 237 | I | | 0.024 | 0.044 | 0.044 | 889 | I | 0.09 | 0.051 | 0.051 |
| 238 | N | | 0.091 | 0.124 | 0.124 | 890 | D | 0.059 | 0.024 | 0.024 |
| 239 | P | | 0.107 | 0.114 | 0.114 | 891 | L | 0.018 | 0.012 | 0.012 |
| 240 | N | | 0.103 | 0.154 | 0.154 | 892 | S | 0.006 | 0.004 | 0.004 |
| 241 | R | | 0.095 | 0.146 | 0.146 | 893 | R | 0.036 | 0.033 | 0.033 |
| 242 | V | | 0.072 | 0.091 | 0.091 | 894 | Y | 0.119 | 0.125 | 0.125 |
| 243 | F | | 0.017 | 0.023 | 0.023 | 895 | A | 0.156 | 0.134 | 0.134 |
| 244 | K | | 0.102 | 0.068 | 0.068 | 896 | S | 0.416 | 0.39 | 0.39 |
| 245 | V | | 0.035 | 0.027 | 0.027 | 897 | K | 0.459 | 0.404 | 0.405 |
| 246 | N | | 0.099 | 0.071 | 0.072 | 898 | I | 0.156 | 0.133 | 0.133 |
| 247 | T | | 0.092 | 0.053 | 0.053 | 899 | N | 0.335 | 0.289 | 0.29 |
| 248 | N | | 0.239 | 0.163 | 0.163 | 900 | I | 0.346 | 0.231 | 0.232 |
| 249 | A | | 0.079 | 0.051 | 0.051 | 901 | G | 0.236 | 0.271 | 0.271 |
| 250 | Y | | 0.067 | 0.049 | 0.049 | 902 | S | 0.314 | 0.377 | 0.378 |
| 251 | Y | | 0.04 | 0.022 | 0.022 | 903 | K | 0.46 | 0.591 | 0.591 |
| 252 | E | | 0.018 | 0.013 | 0.013 | 904 | V | 0.374 | 0.496 | 0.497 |
| 253 | M | | 0.011 | 0.01 | 0.01 | 905 | N | 0.323 | 0.436 | 0.435 |
| 254 | S | | 0.033 | 0.024 | 0.024 | 906 | F | 0.201 | 0.232 | 0.232 |
| 255 | G | | 0.026 | 0.021 | 0.021 | 907 | D | 0.061 | 0.056 | 0.055 |
| 256 | L | | 0.022 | 0.021 | 0.021 | 908 | P | 0.062 | 0.062 | 0.062 |
| 257 | E | | 0.094 | 0.104 | 0.104 | 909 | I | 0.053 | 0.053 | 0.053 |
| 258 | V | | 0.015 | 0.026 | 0.026 | 910 | D | 0.054 | 0.067 | 0.067 |
| 259 | S | | 0.02 | 0.028 | 0.028 | 911 | K | 0.103 | 0.094 | 0.094 |
| 260 | F | | 0.018 | 0.031 | 0.031 | 912 | N | 0.033 | 0.027 | 0.027 |
| 261 | E | | 0.028 | 0.035 | 0.035 | 913 | Q | 0.049 | 0.047 | 0.047 |
| 262 | E | | 0.032 | 0.037 | 0.037 | 914 | I | 0.033 | 0.029 | 0.029 |
| 263 | L | | 0.008 | 0.01 | 0.01 | 915 | Q | 0.088 | 0.097 | 0.097 |
| 264 | R | | 0.027 | 0.035 | 0.035 | 916 | L | 0.002 | 0.005 | 0.005 |
| 265 | T | | 0.008 | 0.012 | 0.012 | 917 | F | 0.373 | 0.506 | 0.506 |
| 266 | F | | 0.019 | 0.021 | 0.021 | 918 | N | 0.185 | 0.208 | 0.208 |
| 267 | G | | 0.016 | 0.012 | 0.012 | 919 | L | 0.326 | 0.46 | 0.46 |
| 268 | G | | 0.012 | 0.01 | 0.01 | 920 | E | 0.207 | 0.248 | 0.249 |
| 269 | H | | 0.017 | 0.016 | 0.016 | 921 | S | 0.129 | 0.129 | 0.129 |
| 270 | D | | 0.012 | 0.012 | 0.012 | 922 | S | 0.039 | 0.055 | 0.055 |
| 271 | A | | 0.036 | 0.027 | 0.027 | 923 | K | 0.066 | 0.044 | 0.044 |
| 272 | K | | 0.057 | 0.058 | 0.058 | 924 | I | 0.029 | 0.015 | 0.015 |
| 273 | F | | 0.013 | 0.013 | 0.013 | 925 | E | 0.097 | 0.067 | 0.067 |
| 274 | I | | 0.036 | 0.037 | 0.037 | 926 | V | 0.008 | 0.003 | 0.003 |
| 275 | D | | 0.027 | 0.028 | 0.028 | 927 | I | 0.29 | 0.341 | 0.341 |
| 276 | S | | 0.102 | 0.094 | 0.094 | 928 | L | 0.058 | 0.044 | 0.044 |
| 277 | L | | 0.101 | 0.112 | 0.112 | 929 | K | 0.375 | 0.276 | 0.276 |
| 278 | Q | | 0.072 | 0.092 | 0.092 | 930 | N | 0.371 | 0.402 | 0.403 |
| 279 | E | | 0.29 | 0.292 | 0.292 | 931 | A | 0.347 | 0.269 | 0.27 |
| 280 | N | | 0.333 | 0.337 | 0.337 | 932 | I | 0.082 | 0.076 | 0.076 |
| 281 | E | | 0.29 | 0.353 | 0.354 | 933 | V | 0.139 | 0.227 | 0.228 |
| 282 | F | | 0.155 | 0.195 | 0.196 | 934 | Y | 0.049 | 0.044 | 0.044 |
| 283 | R | | 0.36 | 0.412 | 0.413 | 935 | N | 0.057 | 0.057 | 0.057 |
| 284 | L | | 0.362 | 0.348 | 0.348 | 936 | S | 0.017 | 0.029 | 0.029 |
| 285 | Y | | 0.28 | 0.292 | 0.292 | 937 | M | 0.029 | 0.022 | 0.022 |
| 286 | Y | | 0.154 | 0.192 | 0.192 | 938 | Y | 0.013 | 0.011 | 0.011 |
| 287 | Y | | 0.206 | 0.199 | 0.199 | 939 | E | 0.014 | 0.022 | 0.022 |
| 288 | N | | 0.199 | 0.181 | 0.181 | 940 | N | 0.01 | 0.009 | 0.009 |
| 289 | K | | 0.07 | 0.059 | 0.059 | 941 | F | 0.002 | 0.002 | 0.002 |
| 290 | F | | 0.024 | 0.033 | 0.033 | 942 | S | 0.005 | 0.002 | 0.002 |
| 291 | K | | 0.09 | 0.086 | 0.086 | 943 | T | 0.001 | 0 | 0 |
| 292 | D | | 0.074 | 0.063 | 0.063 | 944 | S | 0.01 | 0.003 | 0.003 |
| 293 | I | | 0.014 | 0.015 | 0.015 | 945 | F | 0.006 | 0.005 | 0.005 |
| 294 | A | | 0.011 | 0.011 | 0.011 | 946 | W | 0.045 | 0.032 | 0.032 |
| 295 | S | | 0.015 | 0.015 | 0.015 | 947 | I | 0.002 | 0.002 | 0.002 |
| 296 | T | | 0.014 | 0.015 | 0.015 | 948 | R | 0.017 | 0.021 | 0.021 |
| 297 | L | | 0.009 | 0.012 | 0.012 | 949 | I | 0.003 | 0.005 | 0.005 |
| 298 | N | | 0.024 | 0.022 | 0.022 | 950 | P | 0.007 | 0.005 | 0.005 |
| 299 | K | | 0.032 | 0.049 | 0.049 | 951 | K | 0.008 | 0.011 | 0.011 |
| 300 | A | | 0.022 | 0.02 | 0.02 | 952 | Y | 0.018 | 0.016 | 0.017 |
| 301 | K | | 0.07 | 0.079 | 0.079 | 953 | F | 0.061 | 0.07 | 0.078 |
| 302 | S | | 0.116 | 0.114 | 0.114 | 954 | N | 0.183 | 0.143 | 0.135 |
| 303 | I | | 0.043 | 0.059 | 0.059 | 955 | S | 0.131 | 0.312 | 0.266 |
| 304 | V | | 0.222 | 0.228 | 0.229 | 956 | I | 0.214 | 0.18 | 0.188 |
| 305 | G | | 0.11 | 0.131 | 0.131 | 957 | S | 0.017 | 0.017 | 0.017 |
| 306 | T | | 0.055 | 0.069 | 0.069 | 958 | L | 0.087 | 0.124 | 0.106 |
| 307 | T | | 0.173 | 0.186 | 0.186 | 959 | N | 0.21 | 0.226 | 0.228 |
| 308 | A | | 0.114 | 0.128 | 0.128 | 960 | N | 0.164 | 0.129 | 0.129 |
| 309 | S | | 0.116 | 0.14 | 0.14 | 961 | E | 0.13 | 0.128 | 0.127 |
| 310 | L | | 0.021 | 0.031 | 0.031 | 962 | Y | 0.016 | 0.017 | 0.017 |
| 311 | Q | | 0.102 | 0.127 | 0.127 | 963 | T | 0.025 | 0.024 | 0.024 |
| 312 | Y | | 0.141 | 0.179 | 0.179 | 964 | I | 0.003 | 0.003 | 0.003 |
| 313 | M | | 0.01 | 0.017 | 0.017 | 965 | I | 0 | 0.001 | 0.001 |
| 314 | K | | 0.023 | 0.027 | 0.027 | 966 | N | 0.019 | 0.014 | 0.014 |
| 315 | N | | 0.124 | 0.147 | 0.147 | 967 | C | 0.006 | 0.004 | 0.004 |
| 316 | V | | 0.05 | 0.084 | 0.084 | 968 | M | 0.021 | 0.02 | 0.02 |
| 317 | F | | 0.006 | 0.011 | 0.011 | 969 | E | 0.124 | 0.079 | 0.079 |
| 318 | K | | 0.041 | 0.038 | 0.038 | 970 | N | 0.127 | 0.087 | 0.086 |
| 319 | E | | 0.131 | 0.162 | 0.162 | 971 | N | 0.099 | 0.065 | 0.065 |
| 320 | K | | 0.014 | 0.019 | 0.019 | 972 | S | 0.032 | 0.031 | 0.031 |
| 321 | Y | | 0.008 | 0.014 | 0.014 | 973 | G | 0.007 | 0.006 | 0.006 |
| 322 | L | | 0.065 | 0.103 | 0.103 | 974 | W | 0.003 | 0.002 | 0.002 |
| 323 | L | | 0.025 | 0.034 | 0.034 | 975 | K | 0.018 | 0.019 | 0.019 |
| 324 | S | | 0.065 | 0.067 | 0.067 | 976 | V | 0 | 0.001 | 0.001 |
| 325 | E | | 0.156 | 0.159 | 0.158 | 977 | S | 0.007 | 0.013 | 0.013 |
| 326 | D | | 0.113 | 0.128 | 0.128 | 978 | L | 0.003 | 0.003 | 0.003 |
| 327 | T | | 0.183 | 0.184 | 0.184 | 979 | N | 0.041 | 0.034 | 0.034 |
| 328 | S | | 0.096 | 0.099 | 0.099 | 980 | Y | 0.029 | 0.026 | 0.025 |
| 329 | G | | 0.055 | 0.051 | 0.051 | 981 | G | 0.009 | 0.007 | 0.007 |
| 330 | K | | 0.052 | 0.047 | 0.047 | 982 | E | 0.016 | 0.019 | 0.019 |
| 331 | F | | 0.05 | 0.047 | 0.047 | 983 | I | 0.001 | 0.004 | 0.004 |
| 332 | S | | 0.079 | 0.082 | 0.082 | 984 | I | 0.003 | 0.005 | 0.005 |
| 333 | V | | 0.04 | 0.047 | 0.047 | 985 | W | 0.001 | 0.001 | 0.001 |
| 334 | D | | 0.097 | 0.115 | 0.115 | 986 | T | 0.002 | 0.003 | 0.003 |
| 335 | K | | 0.311 | 0.305 | 0.305 | 987 | L | 0.002 | 0.001 | 0.001 |
| 336 | L | | 0.168 | 0.184 | 0.184 | 988 | Q | 0.029 | 0.027 | 0.027 |
| 337 | K | | 0.124 | 0.162 | 0.162 | 989 | D | 0.022 | 0.024 | 0.024 |
| 338 | F | | 0.048 | 0.049 | 0.049 | 990 | T | 0.079 | 0.067 | 0.067 |
| 339 | D | | 0.323 | 0.349 | 0.349 | 991 | Q | 0.052 | 0.05 | 0.05 |
| 340 | K | | 0.184 | 0.186 | 0.186 | 992 | E | 0.042 | 0.051 | 0.05 |
| 341 | L | | 0.077 | 0.11 | 0.11 | 993 | I | 0.034 | 0.037 | 0.037 |
| 342 | Y | | 0.204 | 0.221 | 0.221 | 994 | K | 0.028 | 0.022 | 0.022 |
| 343 | K | | 0.182 | 0.175 | 0.175 | 995 | Q | 0.012 | 0.008 | 0.008 |
| 344 | M | | 0.05 | 0.054 | 0.054 | 996 | R | 0.009 | 0.007 | 0.007 |
| 345 | L | | 0.03 | 0.041 | 0.041 | 997 | V | 0.008 | 0.004 | 0.004 |
| 346 | T | | 0.077 | 0.098 | 0.098 | 998 | V | 0.01 | 0.007 | 0.007 |
| 347 | E | | 0.11 | 0.133 | 0.133 | 999 | F | 0.035 | 0.029 | 0.029 |
| 348 | I | | 0.022 | 0.029 | 0.029 | 1000 | K | 0.069 | 0.053 | 0.054 |
| 349 | Y | | 0.006 | 0.009 | 0.009 | 1001 | Y | 0.255 | 0.258 | 0.268 |
| 350 | T | | 0.01 | 0.011 | 0.011 | 1002 | S | 0.214 | 0.277 | 0.374 |
| 351 | E | | 0.004 | 0.006 | 0.006 | 1003 | Q | 0.023 | 0.017 | 0.015 |
| 352 | D | | 0.01 | 0.009 | 0.009 | 1004 | M | 0.403 | 0.613 | 0.792 |
| 353 | N | | 0.01 | 0.01 | 0.01 | 1005 | I | 0.549 | 0.653 | 0.754 |
| 354 | F | | 0.002 | 0.002 | 0.002 | 1006 | N | 0.61 | 0.694 | 0.808 |
| 355 | V | | 0.004 | 0.002 | 0.002 | 1007 | I | 0.49 | 0.596 | 0.667 |
| 356 | K | | 0.03 | 0.031 | 0.031 | 1008 | S | 0.043 | 0.08 | 0.075 |
| 357 | F | | 0.025 | 0.028 | 0.028 | 1009 | D | 0.593 | 0.669 | 0.701 |
| 358 | F | | 0.006 | 0.005 | 0.005 | 1010 | Y | 0.462 | 0.509 | 0.533 |
| 359 | K | | 0.018 | 0.034 | 0.034 | 1011 | I | 0.03 | 0.041 | 0.042 |
| 360 | V | | 0.003 | 0.003 | 0.003 | 1012 | N | 0.028 | 0.034 | 0.035 |
| 361 | L | | 0.011 | 0.009 | 0.009 | 1013 | R | 0.113 | 0.127 | 0.132 |
| 362 | N | | 0.008 | 0.006 | 0.006 | 1014 | W | 0.148 | 0.179 | 0.18 |
| 363 | R | | 0.018 | 0.017 | 0.017 | 1015 | I | 0.033 | 0.047 | 0.047 |
| 364 | K | | 0.017 | 0.015 | 0.015 | 1016 | F | 0.057 | 0.032 | 0.032 |
| 365 | T | | 0.007 | 0.008 | 0.008 | 1017 | V | 0.001 | 0.001 | 0.001 |
| 366 | Y | | 0.012 | 0.017 | 0.017 | 1018 | T | 0.001 | 0.001 | 0.001 |
| 367 | L | | 0.008 | 0.009 | 0.009 | 1019 | I | 0 | 0 | 0 |
| 368 | N | | 0.005 | 0.005 | 0.005 | 1020 | T | 0.014 | 0.007 | 0.007 |
| 369 | F | | 0.017 | 0.019 | 0.019 | 1021 | N | 0 | 0 | 0 |
| 370 | D | | 0.019 | 0.026 | 0.026 | 1022 | N | 0.011 | 0.011 | 0.011 |
| 371 | K | | 0.309 | 0.281 | 0.281 | 1023 | R | 0.005 | 0.005 | 0.005 |
| 372 | A | | 0.071 | 0.06 | 0.06 | 1024 | L | 0.008 | 0.009 | 0.009 |
| 373 | V | | 0.005 | 0.004 | 0.004 | 1025 | N | 0.017 | 0.011 | 0.011 |
| 374 | F | | 0.019 | 0.018 | 0.018 | 1026 | N | 0.017 | 0.017 | 0.017 |
| 375 | K | | 0.089 | 0.055 | 0.056 | 1027 | S | 0.003 | 0.001 | 0.001 |
| 376 | I | | 0.049 | 0.036 | 0.036 | 1028 | K | 0.084 | 0.018 | 0.018 |
| 377 | N | | 0.08 | 0.071 | 0.071 | 1029 | I | 0.003 | 0.003 | 0.003 |
| 378 | I | | 0.015 | 0.012 | 0.012 | 1030 | Y | 0.41 | 0.263 | 0.263 |
| 379 | V | | 0.087 | 0.085 | 0.086 | 1031 | I | 0.027 | 0.017 | 0.017 |
| 380 | P | | 0.172 | 0.161 | 0.161 | 1032 | N | 0.462 | 0.336 | 0.337 |
| 381 | K | | 0.23 | 0.222 | 0.223 | 1033 | G | 0.099 | 0.053 | 0.053 |
| 382 | V | | 0.284 | 0.269 | 0.27 | 1034 | R | 0.453 | 0.155 | 0.154 |
| 383 | N | | 0.344 | 0.303 | 0.303 | 1035 | L | 0.367 | 0.122 | 0.122 |
| 384 | Y | | 0.13 | 0.11 | 0.11 | 1036 | I | 0.093 | 0.044 | 0.044 |
| 385 | T | | 0.186 | 0.155 | 0.155 | 1037 | D | 0.03 | 0.021 | 0.021 |
| 386 | I | | 0.156 | 0.158 | 0.157 | 1038 | Q | 0.112 | 0.034 | 0.034 |
| 387 | Y | | 0.256 | 0.241 | 0.241 | 1039 | K | 0.078 | 0.059 | 0.059 |
| 388 | D | | 0.077 | 0.075 | 0.075 | 1040 | P | 0.017 | 0.014 | 0.014 |
| 389 | G | | 0.009 | 0.006 | 0.006 | 1041 | I | 0.003 | 0.002 | 0.002 |
| 390 | F | | 0.079 | 0.061 | 0.061 | 1042 | S | 0.01 | 0.011 | 0.011 |
| 391 | N | | 0.033 | 0.034 | 0.034 | 1043 | N | 0.02 | 0.018 | 0.018 |
| 392 | L | | 0.182 | 0.186 | 0.186 | 1044 | L | 0.018 | 0.023 | 0.023 |
| 393 | R | | 0.172 | 0.173 | 0.173 | 1045 | G | 0.029 | 0.034 | 0.034 |
| 394 | N | | 0.141 | 0.143 | 0.143 | 1046 | N | 0.045 | 0.047 | 0.047 |
| 395 | T | | 0.236 | 0.242 | 0.242 | 1047 | I | 0.017 | 0.019 | 0.019 |
| 396 | N | | 0.174 | 0.177 | 0.177 | 1048 | H | 0.106 | 0.078 | 0.078 |
| 397 | L | | 0.228 | 0.236 | 0.236 | 1049 | A | 0.067 | 0.053 | 0.053 |
| 398 | A | | 0.072 | 0.076 | 0.076 | 1050 | S | 0.07 | 0.034 | 0.034 |
| 399 | A | | 0.129 | 0.111 | 0.111 | 1051 | N | 0.236 | 0.233 | 0.233 |
| 400 | N | | 0.119 | 0.095 | 0.095 | 1052 | N | 0.095 | 0.08 | 0.08 |
| 401 | F | | 0.051 | 0.055 | 0.055 | 1053 | I | 0.005 | 0.003 | 0.003 |
| 402 | N | | 0.121 | 0.142 | 0.143 | 1054 | M | 0.027 | 0.028 | 0.028 |
| 403 | G | | 0.024 | 0.021 | 0.021 | 1055 | F | 0.003 | 0.006 | 0.006 |
| 404 | Q | | 0.002 | 0.003 | 0.003 | 1056 | K | 0.015 | 0.018 | 0.018 |
| 405 | N | | 0.009 | 0.012 | 0.012 | 1057 | L | 0.03 | 0.025 | 0.025 |
| 406 | T | | 0.013 | 0.022 | 0.022 | 1058 | D | 0.053 | 0.054 | 0.054 |
| 407 | E | | 0.061 | 0.08 | 0.08 | 1059 | G | 0.105 | 0.095 | 0.095 |
| 408 | I | | 0.223 | 0.191 | 0.191 | 1060 | C | 0.277 | 0.254 | 0.254 |
| 409 | N | | 0.176 | 0.19 | 0.19 | 1061 | R | 0.377 | 0.375 | 0.387 |
| 410 | N | | 0.109 | 0.114 | 0.114 | 1062 | D | 0.212 | 0.241 | 0.244 |
| 411 | M | | 0.306 | 0.285 | 0.286 | 1063 | T | 0.381 | 0.457 | 0.46 |
| 412 | N | | 0.265 | 0.257 | 0.257 | 1064 | H | 0.438 | 0.564 | 0.566 |
| 413 | F | | 0.162 | 0.129 | 0.129 | 1065 | R | 0.057 | 0.03 | 0.03 |
| 414 | T | | 0.147 | 0.106 | 0.107 | 1066 | Y | 0.215 | 0.206 | 0.206 |
| 415 | K | | 0.064 | 0.047 | 0.047 | 1067 | I | 0.006 | 0.007 | 0.007 |
| 416 | L | | 0.106 | 0.067 | 0.067 | 1068 | W | 0.097 | 0.11 | 0.11 |
| 417 | K | | 0.212 | 0.203 | 0.203 | 1069 | I | 0.007 | 0.011 | 0.011 |
| 418 | N | | 0.276 | 0.23 | 0.23 | 1070 | K | 0.034 | 0.028 | 0.028 |
| 419 | F | | 0.279 | 0.203 | 0.204 | 1071 | Y | 0.008 | 0.005 | 0.005 |
| 420 | T | | 0.026 | 0.02 | 0.02 | 1072 | F | 0.004 | 0.003 | 0.003 |
| 421 | G | | 0.224 | 0.214 | 0.215 | 1073 | N | 0.007 | 0.002 | 0.002 |
| 422 | L | | 0.475 | 0.449 | 0.449 | 1074 | L | 0.003 | 0.001 | 0.001 |
| 423 | F | | 0.46 | 0.384 | 0.384 | 1075 | F | 0.012 | 0.004 | 0.004 |
| 424 | E | | 0.183 | 0.19 | 0.191 | 1076 | D | 0.045 | 0.027 | 0.027 |
| 425 | F | | 0.057 | 0.054 | 0.054 | 1077 | K | 0.062 | 0.057 | 0.057 |
| 426 | Y | | 0.163 | 0.144 | 0.144 | 1078 | E | 0.009 | 0.011 | 0.011 |
| 427 | K | | 0.019 | 0.013 | 0.013 | 1079 | L | 0.042 | 0.018 | 0.018 |
| 428 | L | | 0.099 | 0.259 | 0.259 | 1080 | N | 0.119 | 0.045 | 0.045 |
| 429 | L | | 0.016 | 0.014 | 0.014 | 1081 | E | 0.176 | 0.086 | 0.086 |
| 430 | C | | 0.171 | 0.214 | 0.214 | 1082 | K | 0.057 | 0.027 | 0.027 |
| 431 | V | | 0.049 | 0.045 | 0.045 | 1083 | E | 0.043 | 0.02 | 0.019 |
| 432 | R | | 0.423 | 0.473 | 0.473 | 1084 | I | 0.045 | 0.054 | 0.054 |
| 433 | G | | 0.127 | 0.13 | 0.13 | 1085 | K | 0.174 | 0.087 | 0.086 |
| 451 | N | | 0.325 | 0.333 | 0.333 | 1086 | D | 0.06 | 0.029 | 0.029 |
| 452 | D | | 0.377 | 0.389 | 0.389 | 1087 | L | 0.027 | 0.013 | 0.013 |
| 453 | L | | 0.163 | 0.146 | 0.146 | 1088 | Y | 0.381 | 0.282 | 0.282 |
| 454 | C | | 0.379 | 0.395 | 0.395 | 1089 | D | 0.073 | 0.064 | 0.064 |
| 455 | I | | 0.181 | 0.147 | 0.147 | 1090 | N | 0.026 | 0.023 | 0.023 |
| 456 | K | | 0.171 | 0.156 | 0.156 | 1091 | Q | 0.035 | 0.033 | 0.033 |
| 457 | V | | 0.012 | 0.008 | 0.008 | 1092 | S | 0.064 | 0.061 | 0.061 |
| 458 | N | | 0.099 | 0.087 | 0.087 | 1093 | N | 0.072 | 0.085 | 0.085 |
| 459 | N | | 0.021 | 0.012 | 0.012 | 1094 | S | 0.038 | 0.041 | 0.04 |
| 460 | W | | 0.045 | 0.033 | 0.033 | 1095 | G | 0.068 | 0.063 | 0.063 |
| 461 | D | | 0.075 | 0.049 | 0.049 | 1096 | I | 0.075 | 0.083 | 0.083 |
| 462 | L | | 0.008 | 0.006 | 0.006 | 1097 | L | 0.004 | 0.006 | 0.006 |
| 463 | F | | 0.016 | 0.011 | 0.011 | 1098 | K | 0.034 | 0.04 | 0.04 |
| 464 | F | | 0.004 | 0.004 | 0.004 | 1099 | D | 0.012 | 0.017 | 0.017 |
| 465 | S | | 0.013 | 0.009 | 0.009 | 1100 | F | 0.057 | 0.034 | 0.032 |
| 466 | P | | 0.007 | 0.007 | 0.007 | 1101 | W | 0.009 | 0.011 | 0.011 |
| 467 | S | | 0.01 | 0.009 | 0.009 | 1102 | G | 0.018 | 0.018 | 0.018 |
| 468 | E | | 0.077 | 0.056 | 0.056 | 1103 | D | 0.049 | 0.062 | 0.062 |
| 469 | D | | 0.028 | 0.024 | 0.025 | 1104 | Y | 0.094 | 0.094 | 0.094 |
| 470 | N | | 0.014 | 0.018 | 0.018 | 1105 | L | 0.016 | 0.018 | 0.018 |
| 471 | F | | 0.022 | 0.02 | 0.02 | 1106 | Q | 0.047 | 0.054 | 0.054 |
| 472 | T | | 0.089 | 0.1 | 0.099 | 1107 | Y | 0.049 | 0.047 | 0.047 |
| 473 | N | | 0.057 | 0.048 | 0.049 | 1108 | D | 0.043 | 0.055 | 0.055 |
| 474 | D | | 0.088 | 0.084 | 0.084 | 1109 | K | 0.042 | 0.053 | 0.052 |
| 475 | L | | 0.014 | 0.016 | 0.016 | 1110 | P | 0.043 | 0.045 | 0.045 |
| 476 | N | | 0.134 | 0.152 | 0.152 | 1111 | Y | 0.013 | 0.017 | 0.016 |
| 477 | K | | 0.049 | 0.052 | 0.052 | 1112 | Y | 0.028 | 0.02 | 0.019 |
| 478 | G | | 0.063 | 0.09 | 0.09 | 1113 | M | 0.005 | 0.006 | 0.006 |
| 479 | E | | 0.02 | 0.025 | 0.025 | 1114 | L | 0.1 | 0.13 | 0.136 |
| 480 | E | | 0.105 | 0.149 | 0.15 | 1115 | N | 0.113 | 0.192 | 0.201 |
| 481 | I | | 0.013 | 0.017 | 0.017 | 1116 | L | 0.528 | 0.577 | 0.574 |
| 482 | T | | 0.047 | 0.056 | 0.057 | 1117 | Y | 0.652 | 0.499 | 0.505 |
| 483 | S | | 0.089 | 0.089 | 0.089 | 1118 | D | 0.161 | 0.199 | 0.198 |
| 484 | D | | 0.092 | 0.108 | 0.108 | 1119 | P | 0.523 | 0.611 | 0.607 |
| 485 | T | | 0.04 | 0.055 | 0.055 | 1120 | N | 0.311 | 0.343 | 0.389 |
| 486 | N | | 0.048 | 0.052 | 0.052 | 1121 | K | 0.063 | 0.044 | 0.041 |
| 493 | N | | 0.08 | 0.099 | 0.099 | 1122 | Y | 0.112 | 0.07 | 0.066 |
| 494 | I | | 0.098 | 0.084 | 0.084 | 1123 | V | 0.008 | 0.006 | 0.006 |
| 495 | S | | 0.075 | 0.064 | 0.064 | 1124 | D | 0.084 | 0.043 | 0.036 |
| 496 | L | | 0.075 | 0.06 | 0.06 | 1125 | V | 0.054 | 0.042 | 0.026 |
| 497 | D | | 0.369 | 0.3 | 0.301 | 1126 | N | 0.111 | 0.06 | 0.05 |
| 498 | L | | 0.093 | 0.084 | 0.084 | 1127 | N | 0.133 | 0.091 | 0.057 |
| 499 | I | | 0.008 | 0.01 | 0.01 | 1128 | V | 0.085 | 0.062 | 0.037 |
| 500 | Q | | 0.469 | 0.408 | 0.409 | 1129 | G | 0.06 | 0.054 | 0.046 |
| 501 | Q | | 0.502 | 0.382 | 0.383 | 1130 | I | 0.201 | 0.12 | 0.098 |
| 502 | Y | | 0.168 | 0.144 | 0.144 | 1131 | R | 0.252 | 0.157 | 0.155 |
| 503 | Y | | 0.313 | 0.26 | 0.26 | 1132 | G | 0.056 | 0.038 | 0.022 |
| 504 | L | | 0.538 | 0.451 | 0.451 | 1133 | Y | 0.16 | 0.087 | 0.067 |
| 505 | T | | 0.346 | 0.318 | 0.318 | 1134 | M | 0.013 | 0.016 | 0.008 |
| 506 | F | | 0.131 | 0.135 | 0.135 | 1135 | Y | 0.214 | 0.088 | 0.078 |
| 507 | N | | 0.247 | 0.247 | 0.247 | 1136 | L | 0.035 | 0.032 | 0.032 |
| 508 | F | | 0.137 | 0.132 | 0.132 | 1137 | K | 0.142 | 0.101 | 0.092 |
| 509 | D | | 0.27 | 0.257 | 0.256 | 1138 | G | 0.087 | 0.042 | 0.057 |
| 510 | N | | 0.158 | 0.177 | 0.177 | 1139 | P | 0.109 | 0.089 | 0.147 |
| 511 | E | | 0.064 | 0.078 | 0.078 | 1140 | R | 0.217 | 0.1 | 0.152 |
| 512 | P | | 0.013 | 0.021 | 0.021 | 1141 | G | 0.058 | 0.162 | 0.239 |
| 513 | E | | 0.076 | 0.09 | 0.09 | 1142 | S | 0.39 | 0.566 | 0.797 |
| 514 | N | | 0.035 | 0.03 | 0.03 | 1143 | V | 0.027 | 0.028 | 0.034 |
| 515 | I | | 0.078 | 0.085 | 0.085 | 1144 | M | 0.218 | 0.294 | 0.399 |
| 516 | S | | 0.132 | 0.146 | 0.145 | 1145 | T | 0.038 | 0.038 | 0.03 |
| 517 | I | | 0.042 | 0.058 | 0.058 | 1146 | T | 0.221 | 0.237 | 0.219 |
| 518 | E | | 0.162 | 0.203 | 0.203 | 1147 | N | 0.179 | 0.402 | 0.408 |
| 519 | N | | 0.191 | 0.238 | 0.238 | 1148 | I | 0.07 | 0.13 | 0.157 |
| 520 | L | | 0.18 | 0.227 | 0.228 | 1149 | Y | 0.049 | 0.101 | 0.158 |
| 521 | S | | 0.255 | 0.332 | 0.333 | 1150 | L | 0.294 | 0.575 | 0.768 |
| 522 | S | | 0.203 | 0.253 | 0.254 | 1151 | N | 0.076 | 0.083 | 0.061 |
| 523 | D | | 0.161 | 0.185 | 0.185 | 1152 | S | 0.18 | 0.128 | 0.262 |
| 524 | I | | 0.099 | 0.094 | 0.095 | 1153 | S | 0.036 | 0.067 | 0.074 |
| 525 | I | | 0.507 | 0.54 | 0.542 | 1154 | L | 0.053 | 0.035 | 0.029 |
| 526 | G | | 0.174 | 0.136 | 0.136 | 1155 | Y | 0.062 | 0.062 | 0.057 |
| 527 | Q | | 0.554 | 0.573 | 0.574 | 1156 | R | 0.109 | 0.086 | 0.075 |
| 528 | L | | 0.204 | 0.21 | 0.211 | 1157 | G | 0.03 | 0.027 | 0.022 |
| 529 | E | | 0.307 | 0.305 | 0.306 | 1158 | T | 0.011 | 0.008 | 0.006 |
| 530 | L | | 0.472 | 0.51 | 0.51 | 1159 | K | 0.023 | 0.025 | 0.024 |
| 531 | M | | 0.307 | 0.311 | 0.312 | 1160 | F | 0.008 | 0.011 | 0.01 |
| 532 | P | | 0.222 | 0.246 | 0.247 | 1161 | I | 0.022 | 0.032 | 0.032 |
| 533 | N | | 0.428 | 0.45 | 0.451 | 1162 | I | 0.005 | 0.006 | 0.006 |
| 534 | I | | 0.19 | 0.206 | 0.207 | 1163 | K | 0.052 | 0.065 | 0.065 |
| 535 | E | | 0.271 | 0.262 | 0.263 | 1164 | K | 0.044 | 0.051 | 0.051 |
| 536 | R | | 0.1 | 0.101 | 0.101 | 1165 | Y | 0.07 | 0.083 | 0.083 |
| 537 | F | | 0.041 | 0.032 | 0.032 | 1166 | A | 0.086 | 0.067 | 0.067 |
| 538 | P | | 0.125 | 0.082 | 0.081 | 1169 | N | 0.059 | 0.056 | 0.056 |
| 539 | N | | 0.054 | 0.043 | 0.043 | 1170 | K | 0.088 | 0.096 | 0.096 |
| 540 | G | | 0.134 | 0.137 | 0.137 | 1171 | D | 0.047 | 0.051 | 0.051 |
| 541 | K | | 0.154 | 0.124 | 0.124 | 1172 | N | 0.047 | 0.049 | 0.049 |
| 542 | K | | 0.42 | 0.452 | 0.452 | 1173 | I | 0.079 | 0.079 | 0.079 |
| 543 | Y | | 0.18 | 0.158 | 0.157 | 1174 | V | 0.021 | 0.02 | 0.02 |
| 544 | E | | 0.469 | 0.457 | 0.456 | 1175 | R | 0.06 | 0.043 | 0.043 |
| 545 | L | | 0.13 | 0.128 | 0.128 | 1176 | N | 0.039 | 0.037 | 0.037 |
| 546 | D | | 0.34 | 0.347 | 0.347 | 1177 | N | 0.08 | 0.079 | 0.078 |
| 547 | K | | 0.124 | 0.116 | 0.116 | 1178 | D | 0.042 | 0.03 | 0.03 |
| 548 | Y | | 0.18 | 0.154 | 0.154 | 1179 | R | 0.083 | 0.077 | 0.077 |
| 549 | T | | 0.026 | 0.023 | 0.023 | 1180 | V | 0.007 | 0.01 | 0.01 |
| 550 | M | | 0.005 | 0.004 | 0.004 | 1181 | Y | 0.07 | 0.084 | 0.084 |
| 551 | F | | 0.003 | 0.003 | 0.003 | 1182 | I | 0.005 | 0.004 | 0.004 |
| 552 | H | | 0.068 | 0.053 | 0.053 | 1183 | N | 0.018 | 0.029 | 0.028 |
| 553 | Y | | 0.029 | 0.044 | 0.044 | 1184 | V | 0.002 | 0.002 | 0.002 |
| 554 | L | | 0.009 | 0.006 | 0.006 | 1185 | V | 0.014 | 0.022 | 0.018 |
| 555 | R | | 0.042 | 0.031 | 0.031 | 1186 | V | 0.012 | 0.013 | 0.009 |
| 556 | A | | 0.012 | 0.014 | 0.014 | 1187 | K | 0.053 | 0.045 | 0.032 |
| 557 | Q | | 0.022 | 0.022 | 0.022 | 1188 | N | 0.047 | 0.057 | 0.047 |
| 558 | E | | 0.035 | 0.028 | 0.028 | 1189 | K | 0.064 | 0.062 | 0.046 |
| 559 | F | | 0.041 | 0.058 | 0.058 | 1190 | E | 0.066 | 0.092 | 0.084 |
| 560 | E | | 0.05 | 0.059 | 0.059 | 1191 | Y | 0.038 | 0.047 | 0.035 |
| 561 | H | | 0.014 | 0.025 | 0.025 | 1192 | R | 0.062 | 0.096 | 0.098 |
| 562 | G | | 0.081 | 0.108 | 0.108 | 1193 | L | 0.003 | 0.003 | 0.003 |
| 563 | K | | 0.11 | 0.137 | 0.137 | 1194 | A | 0.004 | 0.007 | 0.007 |
| 564 | S | | 0.096 | 0.112 | 0.112 | 1195 | T | 0.04 | 0.03 | 0.039 |
| 565 | R | | 0.092 | 0.078 | 0.078 | 1196 | N | 0.035 | 0.027 | 0.029 |
| 566 | I | | 0.044 | 0.061 | 0.061 | 1197 | A | 0.218 | 0.136 | 0.12 |
| 567 | A | | 0.075 | 0.069 | 0.069 | 1198 | S | 0.149 | 0.128 | 0.128 |
| 568 | L | | 0.064 | 0.063 | 0.063 | 1199 | Q | 0.08 | 0.067 | 0.066 |
| 569 | T | | 0.073 | 0.055 | 0.055 | 1200 | A | 0.448 | 0.346 | 0.34 |
| 570 | N | | 0.25 | 0.178 | 0.179 | 1201 | G | 0.52 | 0.365 | 0.365 |
| 571 | S | | 0.272 | 0.231 | 0.231 | 1202 | V | 0.589 | 0.52 | 0.518 |
| 572 | V | | 0.11 | 0.074 | 0.074 | 1203 | E | 0.373 | 0.268 | 0.268 |
| 573 | N | | 0.101 | 0.106 | 0.106 | 1204 | K | 0.029 | 0.038 | 0.038 |
| 574 | E | | 0.062 | 0.06 | 0.06 | 1205 | I | 0.313 | 0.103 | 0.101 |
| 575 | A | | 0.015 | 0.016 | 0.016 | 1206 | L | 0.004 | 0.013 | 0.012 |
| 576 | L | | 0.119 | 0.09 | 0.091 | 1207 | S | 0.017 | 0.014 | 0.015 |
| 577 | L | | 0.094 | 0.075 | 0.075 | 1208 | A | 0.008 | 0.007 | 0.005 |
| 578 | N | | 0.093 | 0.096 | 0.096 | 1209 | L | 0.027 | 0.031 | 0.029 |
| 579 | P | | 0.072 | 0.069 | 0.069 | 1210 | E | 0.068 | 0.055 | 0.038 |
| 580 | S | | 0.05 | 0.053 | 0.054 | 1211 | I | 0.143 | 0.144 | 0.112 |
| 581 | R | | 0.08 | 0.071 | 0.071 | 1212 | P | 0.105 | 0.119 | 0.121 |
| 582 | V | | 0.025 | 0.04 | 0.04 | 1213 | D | 0.134 | 0.117 | 0.1 |
| 583 | Y | | 0.038 | 0.049 | 0.049 | 1214 | V | 0.041 | 0.055 | 0.056 |
| 584 | T | | 0.012 | 0.012 | 0.012 | 1215 | G | 0.112 | 0.123 | 0.132 |
| 585 | F | | 0.042 | 0.03 | 0.03 | 1216 | N | 0.088 | 0.075 | 0.085 |
| 586 | F | | 0.031 | 0.025 | 0.025 | 1217 | L | 0.054 | 0.047 | 0.052 |
| 587 | S | | 0.066 | 0.056 | 0.056 | 1218 | S | 0.013 | 0.018 | 0.018 |
| 588 | S | | 0.165 | 0.117 | 0.117 | 1219 | Q | 0.041 | 0.042 | 0.043 |
| 589 | D | | 0.091 | 0.092 | 0.092 | 1220 | V | 0.039 | 0.043 | 0.043 |
| 590 | Y | | 0.009 | 0.009 | 0.009 | 1221 | V | 0.002 | 0.003 | 0.003 |
| 591 | V | | 0.091 | 0.075 | 0.075 | 1222 | V | 0.026 | 0.029 | 0.029 |
| 592 | K | | 0.121 | 0.1 | 0.1 | 1223 | M | 0.001 | 0.001 | 0.001 |
| 593 | K | | 0.031 | 0.032 | 0.033 | 1224 | K | 0.035 | 0.029 | 0.029 |
| 594 | V | | 0.006 | 0.006 | 0.006 | 1225 | S | 0.013 | 0.013 | 0.013 |
| 595 | N | | 0.201 | 0.18 | 0.181 | 1226 | K | 0.034 | 0.025 | 0.025 |
| 596 | K | | 0.088 | 0.087 | 0.087 | 1227 | N | 0.056 | 0.033 | 0.033 |
| 597 | A | | 0.136 | 0.133 | 0.133 | 1228 | D | 0.081 | 0.068 | 0.068 |
| 598 | T | | 0.086 | 0.102 | 0.103 | 1234 | K | 0.157 | 0.155 | 0.155 |
| 599 | E | | 0.225 | 0.221 | 0.222 | 1235 | C | 0.002 | 0.004 | 0.004 |
| 600 | A | | 0.1 | 0.099 | 0.099 | 1236 | K | 0.019 | 0.019 | 0.019 |
| 601 | A | | 0.257 | 0.27 | 0.27 | 1237 | M | 0.002 | 0.004 | 0.004 |
| 602 | M | | 0.133 | 0.148 | 0.148 | 1238 | N | 0.015 | 0.017 | 0.017 |
| 603 | F | | 0.019 | 0.016 | 0.016 | 1239 | L | 0.003 | 0.004 | 0.004 |
| 604 | L | | 0.192 | 0.212 | 0.212 | 1240 | Q | 0.032 | 0.05 | 0.05 |
| 605 | G | | 0.178 | 0.212 | 0.213 | 1241 | D | 0.031 | 0.044 | 0.044 |
| 606 | W | | 0.041 | 0.059 | 0.06 | 1242 | N | 0.094 | 0.096 | 0.096 |
| 607 | V | | 0.015 | 0.013 | 0.013 | 1243 | N | 0.065 | 0.06 | 0.06 |
| 608 | E | | 0.19 | 0.209 | 0.21 | 1244 | G | 0.046 | 0.044 | 0.044 |
| 609 | Q | | 0.077 | 0.091 | 0.091 | 1245 | N | 0.051 | 0.055 | 0.055 |
| 610 | L | | 0.002 | 0.001 | 0.001 | 1246 | D | 0.031 | 0.04 | 0.04 |
| 611 | V | | 0.086 | 0.09 | 0.09 | 1247 | I | 0.024 | 0.052 | 0.052 |
| 612 | Y | | 0.474 | 0.517 | 0.516 | 1248 | G | 0.021 | 0.01 | 0.01 |
| 613 | D | | 0.028 | 0.029 | 0.029 | 1249 | F | 0.059 | 0.17 | 0.17 |
| 614 | F | | 0.007 | 0.007 | 0.007 | 1250 | I | 0.002 | 0.017 | 0.017 |
| 615 | T | | 0.299 | 0.317 | 0.317 | 1251 | G | 0.023 | 0.036 | 0.037 |
| 616 | D | | 0.27 | 0.294 | 0.293 | 1252 | F | 0.162 | 0.488 | 0.486 |
| 617 | E | | 0.021 | 0.024 | 0.024 | 1253 | H | 0.089 | 0.064 | 0.065 |
| 618 | T | | 0.021 | 0.022 | 0.022 | 1254 | Q | 0.638 | 0.531 | 0.533 |
| 619 | S | | 0.293 | 0.332 | 0.332 | 1255 | F | 0.649 | 0.584 | 0.589 |
| 620 | E | | 0.076 | 0.085 | 0.085 | 1256 | N | 0.498 | 0.454 | 0.464 |
| 621 | V | | 0.249 | 0.3 | 0.3 | 1257 | N | 0.542 | 0.47 | 0.486 |
| 622 | S | | 0.204 | 0.234 | 0.234 | 1258 | I | 0.182 | 0.158 | 0.153 |
| 623 | T | | 0.127 | 0.177 | 0.177 | 1259 | A | 0.071 | 0.07 | 0.063 |
| 624 | T | | 0.272 | 0.274 | 0.274 | 1260 | K | 0.316 | 0.248 | 0.248 |
| 625 | D | | 0.1 | 0.126 | 0.126 | 1261 | L | 0.007 | 0.027 | 0.025 |
| 626 | K | | 0.259 | 0.328 | 0.329 | 1262 | V | 0.065 | 0.009 | 0.009 |
| 627 | I | | 0.048 | 0.042 | 0.042 | 1263 | A | 0.003 | 0.004 | 0.004 |
| 628 | A | | 0.036 | 0.027 | 0.027 | 1264 | S | 0.02 | 0.03 | 0.031 |
| 629 | D | | 0.014 | 0.01 | 0.01 | 1265 | N | 0.109 | 0.13 | 0.13 |
| 630 | I | | 0.009 | 0.007 | 0.007 | 1266 | W | 0.728 | 0.651 | 0.652 |
| 631 | T | | 0.105 | 0.107 | 0.107 | 1267 | Y | 0.644 | 0.533 | 0.533 |
| 632 | I | | 0.13 | 0.154 | 0.154 | 1268 | N | 0.065 | 0.09 | 0.091 |
| 633 | I | | 0.021 | 0.024 | 0.024 | 1269 | R | 0.447 | 0.409 | 0.409 |
| 634 | I | | 0.005 | 0.007 | 0.007 | 1277 | T | 0.673 | 0.549 | 0.549 |
| 635 | P | | 0.011 | 0.017 | 0.017 | 1278 | L | 0.657 | 0.551 | 0.552 |
| 636 | Y | | 0.011 | 0.006 | 0.006 | 1279 | G | 0.008 | 0.014 | 0.014 |
| 637 | I | | 0 | 0.001 | 0.001 | 1280 | C | 0.096 | 0.085 | 0.085 |
| 638 | G | | 0.002 | 0.007 | 0.007 | 1281 | S | 0.026 | 0.037 | 0.037 |
| 639 | P | | 0.019 | 0.021 | 0.021 | 1282 | W | 0.021 | 0.03 | 0.031 |
| 640 | A | | 0.001 | 0 | 0 | 1283 | E | 0.012 | 0.012 | 0.011 |
| 641 | L | | 0.001 | 0.001 | 0.001 | 1284 | F | 0.005 | 0.006 | 0.006 |
| 642 | N | | 0.014 | 0.026 | 0.026 | 1285 | I | 0.002 | 0.002 | 0.002 |
| 643 | I | | 0.009 | 0.009 | 0.009 | 1286 | P | 0.019 | 0.019 | 0.018 |
| 644 | G | | 0.047 | 0.065 | 0.065 | 1287 | V | 0.034 | 0.03 | 0.03 |
| 645 | N | | 0.099 | 0.123 | 0.123 | 1288 | D | 0.026 | 0.026 | 0.025 |
| 646 | M | | 0.055 | 0.086 | 0.087 | 1289 | D | 0.073 | 0.237 | 0.235 |
| 647 | L | | 0.12 | 0.138 | 0.138 | 1290 | G | 0.009 | 0.021 | 0.021 |
| 648 | Y | | 0.208 | 0.227 | 0.227 | 1291 | W | 0.005 | 0.007 | 0.006 |
| 649 | K | | 0.084 | 0.074 | 0.074 | 1292 | G | 0.024 | 0.013 | 0.01 |
| 650 | D | | 0.092 | 0.104 | 0.105 | 1293 | E | 0.021 | 0.076 | 0.068 |
| 651 | D | | 0.096 | 0.131 | 0.131 | 1294 | R | 0.036 | 0.199 | 0.189 |
| 652 | F | | 0.006 | 0.012 | 0.012 |  |  |  |  |  |

Table S3. ScanNet predicts the binding probability of each residue in BoNT/A with SV2C-L4. The BoNT/A residues within 4 Å of SV2C-L4 are rendered in red.

| **Receptor** | **Ligand** | | **K_on_ (M^-1^s^-1^)** (× 10^5^) | **K_off_ (s^-1^)** (× 10^-1^) | **KD (M)** (× 10^-8^) | **Rmax (RU)** |
| --- | --- | --- | --- | --- | --- | --- |
| GST-SV2C-L4 | Hc/A | wt | 12.54 ± 6.46 | 1.20±0.70 | 9.34 ± 0.82 | 62.27 ± 24.10 |
|  |  | wt (pH5.5) | 1.99 | 1.07 | 53.86 | 86.64 |
|  |  | HWTT | 10.09 | 0.55 | 5.42 | 47.40 |
|  |  | VLTT | 7.17 | 0.11 | 1.58 | 63.62 |
|  |  | VLTS | 10.96 ± 1.49 | 0.17±0.03 | 1.56 ± 0.17 | 82.83 ± 29.59 |
|  |  | FLTT | 11.24 ± 1.13 | 0.16±0.02 | 1.42 ± 0.06 | 77.11 ± 30.33 |
|  |  | FLTS | 6.49 | 0.15 | 2.30 | 46.45 |
|  |  | VVTT | 6.08 | 0.16 | 2.60 | 37.08 |
|  | Full length BoNT/A | wt | 22.88 ± 17.77 | 2.48 ± 2.26 | 10.11 ± 1.45 | 21.60 ± 8.37 |
|  |  | VLTS | 12.34 ± 3.47 | 0.42 ± 0.11 | 3.42 ± 0.09 | 31.84 ± 1.03 |

Table S4. Interactions between GST-SV2C-L4 and Hc/A or BoNT/A mutations measured by SPR. Binding parameters were calculated using the Biacore T200 analysis software. Shown is mean ± SD of n = 3 independent replicates for Hc/A wt, VLTS. FLTT, full length BoNT/A wt and VLTS.
